# Hierarchical statistical techniques are necessary to draw reliable conclusions from analysis of isolated cardiomyocyte studies

**DOI:** 10.1093/cvr/cvx151

**Published:** 2017-08-30

**Authors:** Markus B Sikkel, Darrel P Francis, James Howard, Fabiana Gordon, Christina Rowlands, Nicholas S Peters, Alexander R Lyon, Sian E Harding, Kenneth T MacLeod

**Affiliations:** 1 Myocardial Function Section, Fourth Floor, Imperial Centre for Translational and Experimental Medicine, National Heart and Lung Institute, Imperial College London, Hammersmith Campus, Du Cane Road, London, UK; 2 Department of Electrophysiology, Imperial College Healthcare NHS Trust, Hammersmith Hospital, London, UK; 3 Statistics Advisory Service, Imperial College London, London, UK; 4 Department of Cardiology, Royal Brompton Hospital, London, UK

**Keywords:** Hierarchical statistics, Cardiomyocyte, Ca^2+^ transient, Ca^2+^ spark

## Abstract

**Aims:**

It is generally accepted that post-MI heart failure (HF) changes a variety of aspects of sarcoplasmic reticular Ca^2+^ fluxes but for some aspects there is disagreement over whether there is an increase or decrease. The commonest statistical approach is to treat data collected from each cell as independent, even though they are really clustered with multiple likely similar cells from each heart. In this study, we test whether this statistical assumption of independence can lead the investigator to draw conclusions that would be considered erroneous if the analysis handled clustering with specific statistical techniques (hierarchical tests).

**Methods and results:**

Ca^2+^ transients were recorded in cells loaded with Fura-2AM and sparks were recorded in cells loaded with Fluo-4AM. Data were analysed twice, once with the common statistical approach (assumption of independence) and once with hierarchical statistical methodologies designed to allow for any clustering. The statistical tests found that there was significant hierarchical clustering. This caused the common statistical approach to underestimate the standard error and report artificially small *P* values. For example, this would have led to the erroneous conclusion that time to 50% peak transient amplitude was significantly prolonged in HF.

Spark analysis showed clustering, both within each cell and also within each rat, for morphological variables. This means that a three-level hierarchical model is sometimes required for such measures. Standard statistical methodologies, if used instead, erroneously suggest that spark amplitude is significantly greater in HF and spark duration is reduced in HF.

**Conclusion:**

Ca^2+^ fluxes in isolated cardiomyocytes show so much clustering that the common statistical approach that assumes independence of each data point will frequently give the false appearance of statistically significant changes. Hierarchical statistical methodologies need a little more effort, but are necessary for reliable conclusions. We present cost-free simple tools for performing these analyses.

## 1. Introduction

Changes in cellular Ca^2+^ handling are agreed to play a key role in the pathophysiology of heart failure (HF).^1^ The standard experimental model, established for half a century is left anterior descending coronary artery ligation in the rat.^2^ At the whole organ level, there is broad agreement between experimenters on the pattern of haemodynamic and echocardiographic variables.^34–8^

At the cellular level, however, there is more controversy. Changes in Ca^2+^ transient amplitude and kinetics have been widely reported to contribute to organ dysfunction.^1^^,^^9^ However, the 18 studies that report on changes in Ca^2+^ transient kinetics show considerable conflict (*Table 1*). On even smaller scales, Ca^2+^ spark measurements also show convincing changes, again in conflicting directions (see Supplementary material online, *Table S1*). This is not a peculiarity of the rat: mouse studies too show the same conflicts (see Supplementary material online, *TablesS2* and *S3*).

**Table 1:** Summary of changes in electrically evoked Ca^2+^ transients and SR load assessment in studies of rats with post-MI HF. Arrows refer to whether variables (evoked Ca^2+^ transient amplitude and decay time, SR Ca^2+^ content and diastolic [Ca^2+^]_*i*_) were found to be significantly reduced (↓), the same (↔), or increased (↑) in myocytes isolated from post-MI animals compared with control animals in the post-MI rat HF model

Publication	Wks post-MI	Ca^2+^ transient amplitude			Transient decay time			SR Ca^2+^ content			Diastolic [Ca^2+^]_*i*_		
Cheung *et al.*^4^^a^	3					↔							
Huang *et al.*^6^	3						↑						
Zhang *et al.*^7^	3		↔			↔						↔	
Anand^8^	6		↔			↔							
Sande *et al.*^29^	6					↔							
Holt *et al.*^9^	6	↓					↑						↑
Soppa *et al.*^5^	6			↑			↑			↑			↑
Lee *et al.*^30^	7		↔				↑		↔			↔	
Maczewski and Mackiewicz^31^	8		↔				↑		↔				
Kaprielian *et al.*^32^	8			↑					↔			↔	
Loennechen^33^	8			↑			↑						↑
Yoshida *et al.*^34^	8		↔			↔						↔	
Cheng *et al.*^11^	8			↑			↑						
Saraiva *et al.*^35^	9	↓				↔							
Loennechen *et al.*^36^	13			↑			↑						↑
Lyon *et al.*^3^	16						↑						
Lyon *et al.*^37^	16						↑	↓					
Ait Mou *et al.*^38^	18	↓					↑						
Total		3	5	5	0	6	11	1	3	1	0	4	4

a At physiological Ca^2+^ (increased decay time at supraphysiological Ca^2+^ of 5 mM).

The accepted paradigm is that reduced Ca^2+^ transient amplitude contributes to reduced myocardial contractility.^10^ However, the cellular data do not consistently fit this. For example, Ca^2+^ transient amplitudes in HF cells are reported as significantly increased in more studies than decreased (*Table 1*). Moreover, the two studies that reported changes in SR Ca^2+^ content indicated opposite directions of change (*Table 1*). In other variables, studies conflict between showing convincing changes and convincing absence of change (*Table 1*).

There is also no apparent time-dependent shift in variables following MI as would be expected for variables that change from a ‘compensated’ to ‘de-compensated’ cellular phenotype.

Authors have proposed explanations including a compensated phase following MI,^5^ or differences in other elements of the myocyte (e.g. myofilaments) overriding the effects of enhanced Ca^2+^ transients. When one study found enhanced Ca^2+^ amplitudes simultaneously with reduced myocyte contractility, the proposed explanation was a reduction in myofilament sensitivity to Ca^2^^+^.^11^

Rarely considered is the possibility that our statistical methods may have left us open to changes appearing falsely statistically significant. The studies in the field generally used standard statistical tests designed to be valid for independent data points, because these tests are widely available and straightforward to implement.

**Figure 1:**
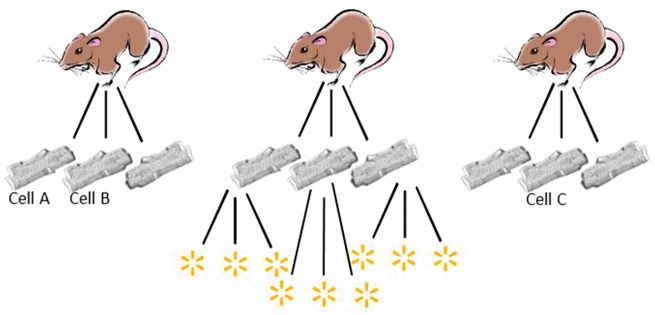
Hierarchical structure of data attained from studies of isolated cardiomyocytes. Multiple cardiomyocytes originate from each isolation. Differences in the animals from which the myocytes originate, as well as slight variations in quality of isolation or experimental conditions on any one day, may result in measurements taken from the myocyte from one rat being more closely related to each other than to measurements from a different isolations. That is, measurements in cell A are more likely to be similar to those in cell B vs. those in cell C in the diagram. A fundamental condition of common statistical tests (e.g. *t*-tests), that of independence of data points, is therefore contravened. An example of a further level of hierarchy is shown for the middle rat with multiple individual sparks recorded from each cell.

The challenge we face is that when we take *n* cells from each of *m* animals we do not truly have *n × m* independent data points.^12^ We have *m* clusters, each containing *n* data points. The data points in each cluster (i.e. the cells from a single animal) will tend to be more similar to each other than they are to points in the other clusters (*Figure 1*). There are well-established statistical techniques for handling data that shows such clustering. Whether using cluster based analysis produces different results from our field’s standard statistical tests, has never been tested.

When analysing such a set of *n × m* data points, investigators tend to take one of two approaches. The more conservative approach is to calculate a mean for each of the m animals and treat these *m* mean values as the only data points. This approach is not popular because it treats the sample size as only *m* and therefore, reduces the ability to detect changes.

The far more popular approach is to ignore the clustering and treat all *n × m* data points as though they are independent. The attraction of this approach is that when the standard statistical tests are performed, the large number of data points lead to the standard error being very small and therefore differences appear to be more statistically significant.^13^ In fact the term ‘pseudoreplication’ has been coined for this.^14^ Studies rarely specify which of these two approaches have been taken but the sample sizes reported generally appear consistent with the latter approach.

A third approach, rarely used, is to recognize the clustered (hierarchical) structure of the data. And use statistical methods designed for this situation.^15^ Hierarchical statistical methods test for clustering and, if present, correct for this when performing significance testing.

In this study we test the application of a hierarchical statistical approach to data from myocytes from the post-MI rat HF model. We examine whether (i) it makes a difference to the conclusions drawn, (ii) whether it is necessary, and (iii) whether it can be implemented conveniently by experimenters.

## 2. Methods

### 2.1 Rat HF model and myocyte imaging

The chronic rat HF model was produced surgically via LAD ligation^3^ with an 8 week delay before echocardiography, culling and biometric measurement as described previously.^16^ Briefly, rats were anesthetized with 2% isoflurane, intubated, and ventilated after preoperative buprenorphine (0.03 mg/kg SC) injection. Loss of righting reflex and pain responses confirmed dequate analgesia and anaesthesia before operating and tying off the LAD with 6-0 prolene. All animal surgical procedures and peri-operative management were carried out in accordance with the Guide for the Care and Use of Laboratory Animals published by the US National Institutes of Health (NIH Publication, 8th Edition, 2011) under assurance number A5634-01. Imperial College Ethical Review Committee authorized the project licence.

Echocardiography and biometric measurements showed a reduced ejection fraction compared with control animals (40 ± 2.2% vs. 83 ± 2.1%, *P* < 0.0001), as well as a significant increase in heart weight: body weight ratio (3.28 ± 0.1 vs. 2.79 ± 0.1, *P* < 0.0001) confirming established HF (see Supplementary material online, *Figure S1*).

Rats were culled by cervical dislocation and cells were isolated 8 weeks following ligation. Cells were loaded with Fura2-AM and imaged using ratiometric techniques as described.^17^ Ca^2+^ transients were recorded at 1 Hz in control (76 cells from 10 isolations) and HF cells (79 cells from 10 isolations) using the ratiometric dye Fura2-AM. The ratio of the isosbestic point for Fura2 (360 nm) to the unbound form (380 nm) was used as a measure of [Ca^2+^]_*i*_. Several variables were assessed using automated transient analysis in IonWizard (Ionoptix Inc.) including diastolic ratio, peak systolic ratio, transient amplitude (peak ratio/diastolic ratio), time to 50% peak, time to 50% decay (TD50), and tau of decay.

In a separate set of experiments isolated cardiomyocytes were loaded with fluo4-AM as described previously^17^ and spontaneous Ca^2+^ sparks were assessed using confocal microscopy. All experiments were performed at 37 °C in NT solution containing 2 mM Ca^2+^. The control group consisted of myocytes isolated from age-matched controls. Data were viewed using Ionwizard and ImageJ (NIH) and analysed with a combination of custom-built macros and Sparkmaster. Amplitude and morphology of five Ca^2+^ transients per cell were averaged to give final result for that cell. Sparks were treated as separate data points.

### 2.2 Statistical analysis

Data were analysed using IBM SPSS Statistics in two ways. Firstly, using our field’s common statistical approach of treating all data points as though they were independent. For each variable, an independent samples *t*-test was performed comparing all the cells from HF hearts against all the cells from control hearts.

The second analysis used hierarchical statistical techniques. These techniques are designed for situations where values may be clustered, with a possibility of similarity within each cluster.^13^ We have designed cost-free methods which we have made available for the reader to use for similar analyses (see Discussion section).

#### 2.2.1 Quantifying clustering

**Figure 2:**
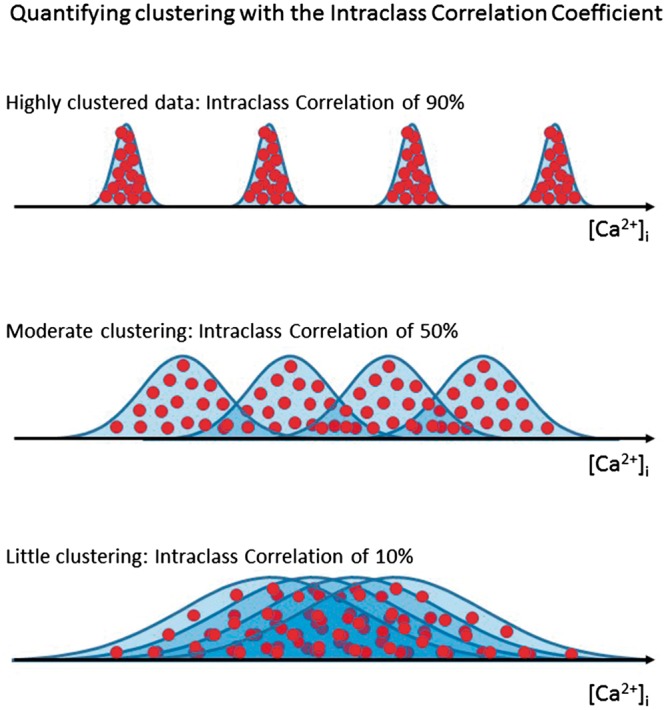
Intraclass correlation in cardiomyocyte studies. Examples of data with high, moderate, or low intraclass correlations (ICCs). Intracellular calcium concentration ([Ca^2+^]_*i*_) from individual cells (red dots) is clustered into histograms making up the data distributions in individual rat hearts. In the top panel, there is a high ICC with data clustered tightly in each heart. In the middle panel, there is an intermediate ICC and in the bottom panel there is a low ICC with a high proportion of the overall variability coming from each individual heart. A larger correction to common statistical techniques is required in data with a high ICC.

The amount of clustering can be quantified by the intraclass correlation coefficient (ICC). This varies between 0% for data showing no clustering, and 100% for intensely clustered data (i.e. each cluster contains multiple identical values, but different clusters have different values). This measure represents the proportion of total variability in an outcome measure that is attributable to the isolation of origin. Illustrative datasets with high, moderate, and low values are shown in *Figure 2*. The method of calculation is shown in Supplementary material online.

#### 2.2.2 Correction of the analysis based on ICC

**Figure 3:**
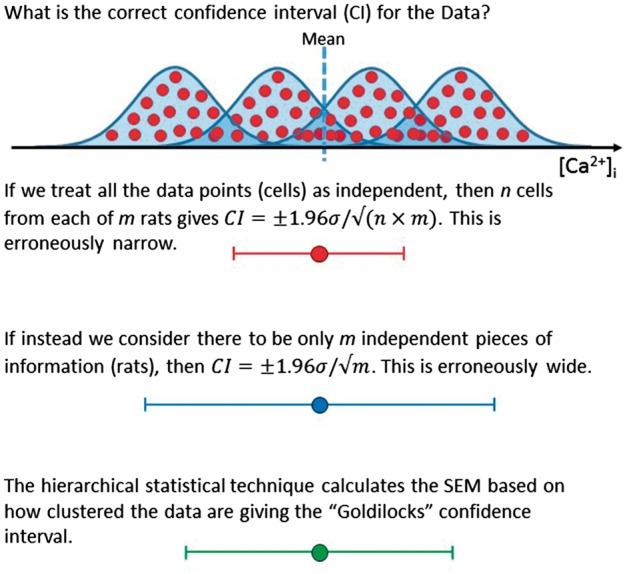
Correction for clustering produced by hierarchical tests. Description of correction to the confidence interval for the difference between control and HF for clustered data that is produced using hierarchical tests.

If the data show no clustering, then the hierarchical model works effectively identically to the commonly used statistical test, treating all the data points as independent. If, on the other hand, the data are tightly grouped within each cluster, with relatively large separation between clusters (as might happen when each day’s isolation was internally homogeneous but differed from other days) then a relatively large correction would need to be applied. The hierarchical test quantifies the amount of clustering and applies the appropriate correction to the statistical significance test (*Figure 3*), further explanation of hierarchical testing is shown in Supplementary material online (see Supplementary methods and *Figure S2*).

#### 2.2.3 Effective sample size

The hierarchical approach also provides a simple method of augmenting conventional sample size calculation to explain the fact that with *m* cells from each of *n* rats the true sample size is less than *n × m* but more than *n*. The correction required depends on the degree of clustering as quantified by the ICC. With low levels of clustering, where each cell’s behaviour is independent of the animal it has come from, effective sample size approaches *n × m*. With high degrees of clustering, where all cells from a single animal behave identically, effective sample size approaches *n*. The calculation of effective sample size is as follows:

For *n* animals, each with *m* cells

$$Effective\;Sample\;Size\;=\frac{n\;\times \;m}{1+(m-1)\times ICC}$$

For example, with 10 animals and 20 cells from each, with an ICC of 50%, effective sample size is neither 200 nor 10, and is calculated as follows:

$$Effective\;Sample\;Size\;=\frac{200}{1+(19)\times 0.5}\approx 19$$

This has important implications for the interpretation of conventional power calculations in the context of hierarchical data. Power calculations are used to work out the appropriate sample size for experiments given a working knowledge of likely effect size and variance obtained from pilot data. In the context of a hierarchical data structure, the sample size given by such power calculations should be considered to be the *Effective Sample Size*. A rearrangement of the above equation would give the true sample size required:

n × m = Effective Sample Size × (1+(m-1)×ICC)

#### 2.2.4 Testing whether the hierarchical statistical model significantly improved the fit

We tested whether the hierarchical statistical model produced a significantly better fit to the data than the commonly used statistical approach, using the *χ*^2^ test of the change in -2 Log Likelihood (*χ*^2^-2LL). This is the recommended method for comparisons of this kind.^18–20^

#### 2.2.5 Transforming non-normal distributions

Initial exploration of data showed that whilst variables pertaining to Ca^2+^ transients were symetrically distributed, spark morphological variables were skewed as has been shown previously.^21^ The logarithmic transformations of morphological characteristics were therefore used to improve this lack of symmetry (see Supplementary material online, *Figure S3*). These transformed variables are referred to as LogAmp, LogFWHM, LogFDHM for the logarithm of spark amplitude, full width at half maximum (FWHM), full duration at half maximum (FDHM).

#### 2.2.6 Running the two analysis approaches

Ca^2+^ transient and spark measures were the dependent variables in the statistical models. The only independent variable was the presence or absence of HF. Each model used was assessed for validity by ensuring predicted values closely corresponded to those observed. Residuals were assessed for normality and symmetry. Estimated marginal means were used to assess significance between control and HF groups.

## 3. Results

Ca^2+^ transient variables were measured from 76 cells from 10 control rats and 79 cells from 10 HF rats. Ca^2+^ spark variables were measured in 344 sparks from 17 cells from 7 control rats and 352 sparks from 22 cells from 5 HF rats.

### 3.1 Multiple cells from each of several rats: Ca2+ transient morphology

For all Ca^2+^ transient morphology variables, the hierarchical model produced a statistically significantly better fit than the commonly used analysis method (*Table 2*). The magnitude of the intraclass correlation (degree of clustering) varied between 12 and 47% (*Table 2*, ICC column).

**Table 2:** Analysis of Ca^2+^ transient morphology variables using standard and hierarchical statistical tests

	Clustering of data (ICC) (%)	Common test of HF vs. control		Hierarchical test of HF vs. control		Comparison of goodness of fit (common vs. hierarchical)
		Std error of difference	*P*-value	Std error of difference	*P*-value	
Diastolic ratio	27	0.0120	0.248	0.0203	0.623	<0.001***
Peak systolic ratio	23	0.0396	0.004**	0.0653	0.046*	0.002**
Transient amplitude (*F*/*F*_0_)	21	0.0306	<0.001***	0.0493	0.010*	0.006**
Time to 50% peak (ms)	12	0.520	0.018*	0.716	0.109	0.021*
Time to 50% decay (ms)	44	4.86	0.444	98.2	0.400	<0.001***
Tau (ms)	47	8.55	0.535	17.7	0.424	<0.001***

Elements of the analysis of each variable are shown. The independent-samples *t*-test is shown as the common test used to compare cellular data. The clustering of data measured by calculating the intraclass correlation (ICC) is shown for each variable. The hierarchical technique is more appropriate with each variable as indicated by better goodness of fit (as measured by *χ*^2^-2LL test). When using the more appropriate hierarchical test the standard error increases and the *P* values also increase making significant differences less likely. For the time to 50% peak this results in a change from a significant test to a non-significant test. Note that the change in standard error is greatest where the ICC is larger.

* *P* < 0.05.

** *P* < 0.01.

*** *P* < 0.001. There were 76 cells from 10 control rats and 79 cells from 10 HF rats.

For all the comparisons between HF and control, the commonly used analysis method reported a more statistically significant difference than the better-fitting hierarchical analysis. The mechanism of this was that the commonly used statistical method produced a smaller value for the standard error of the difference between HF and control (*Table 2*).

**Figure 4:**
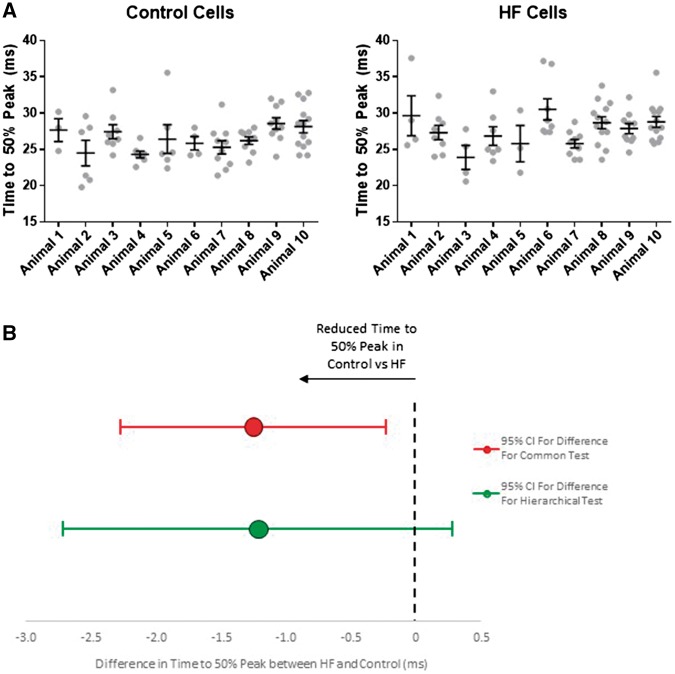
Clustering of time to 50% peak transient amplitude. (*A*) Clustering of time to peak is shown with the time to 50% peak for transient in each cell (grey dots) shown for each rat in HF and control. The mean and standard error bars are shown for each rat. By eye the data appear clustered and this is confirmed by the statistical testing in *Table 2*. (*B*) The mean difference and confidence interval for the difference are shown which indicates why the difference between HF and control becomes non-significant with the corrected confidence intervals of the hierarchical test (green bars) compared with the uncorrected confidence intervals of the common test (red bars). There were 76 cells from 10 control rats and 79 cells from 10 HF rats.

*Figure 4* shows the 95% confidence interval calculated, by the two approaches, for the difference in time to peak between HF and control. The commonly used analysis method gives a different conclusions because it has an artificially small 95% confidence interval, causing the data to meet the criteria for statistical significance.

### 3.2 Multiple sparks from each of multiple cells from multiple rats

For sparks there is potential for an additional level of clustering, because the multiple sparks from each cell could be more similar to each other than the sparks from other cells of the same rat.

The hierarchical analysis was carried out twice (*Table 3*): once allowing only for clustering at the animal level and once allowing for clustering at both the cell and the animal levels. We tested whether the incorporation of the additional (cell) level improved the fit as defined by the *χ*^2^-2LL. Where the fit was improved significantly, the additional level was incorporated into the analysis (methods shown in Supplementary appendix).

**Table 3:** Spark data analysed by hierarchical vs. standard statistical tests

	Clus-tering of data (ICC) (%)	Common method		Two-level hierarchy		Three-level hierarchy		Comparison of goodness of fit (common vs. hierarchical)
		Standard error	*P*-value	Standard error	*P*-value	Standard error	*P*-value	
Variables that have a single value per *cell*, and have clustering within *animal*.								
Spark freq (sp/100 µm/s)	24	0.537	0.886	0.664	0.540	N/A	N/A	0.048*
Variables that have a single value per *spark*, and show clustering within *cell* and within *animal*.								
LogAmp (Δ*F*/*F*_0_)	58	9.00 × 10^−3^	<0.001***	0.0320	0.001**	0.0641	0.239	<0.001***
Variables that have a single value per *spark*, and show clustering within *cell*. There is minimal additional variability between rats such that analysis at cell level hierarchy is most appropriate. Here a comparison of goodness of fit between common and two-level test is significant (*P* < 0.05) but the same comparison between the two-level hierarchy and three-level hierarchy is not significant (*P* > 0.05).								
LogFDHM (ms)	8	0.0168	0.023*	0.0289	0.778	N/A	N/A	<0.001***
LogFWHM (µm)	7	0.0113	0.381	0.0185	0.782	N/A	N/A	<0.001***

Analysis using the common test vs. hierarchical methods is shown. Analysis using a rat-level hierarchy is most appropriate for spark frequency as only a single value is available for each cell. For variables describing spark morphology either an analysis that accounts for both cell-level and rat-level hierarchy is appropriate (as for spark amplitude) or where there is little additional variability per rat, and the goodness of fit is not further improved by a three-level hierarchy, analysis with a cell-level hierarchy is most appropriate (as for FDHM and FWHM). The hierarchical test out-performs the common test for each variable.

* *P* < 0.05.

** *P* < 0.01.

*** *P* < 0.001. There were 344 sparks from 17 cells from 7 control rats and 352 sparks from 22 cells from 5 HF rats.

The *P*-values were substantially different between the commonly used method and the hierarchical analyses. Again, the hierarchical analyses fitted better for each variable. The cause of the difference in *P* values was that the hierarchical models calculated larger values for the standard error.

Even minor clustering made a substantial difference to the *P* value. For example, for logFWHM, even with only 8.5% clustering (as quantified by the ICC), the conventional approach which assumes no clustering reports a *P* value of 0.02, whereas the hierarchical model reports a *P* value of 0.78.

**Figure 5:**
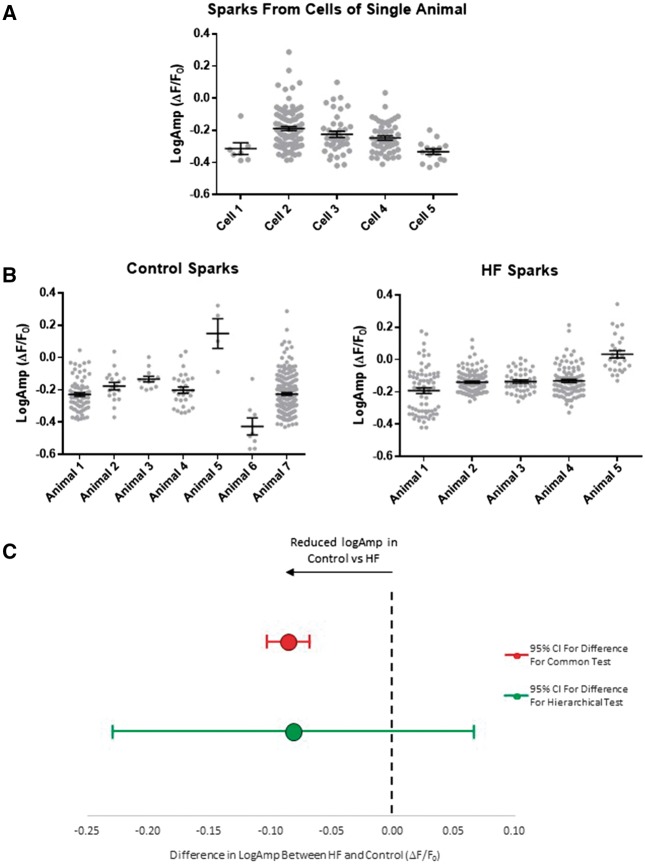
Multi-level clustering of spark logAmp. (*A*) Clustering of spark LogAmp is shown within each of five cells tested from a single rat heart. Each individual spark’s logAmp is shown as a grey dot. The mean and standard error bars are shown for each cell. By eye the data appear clustered at this level and this is confirmed in *Table 3*. (*B*) Spark data may also be further clustered within individual rats. Here sparks are shown grouped by rat. As confirmed in *Table 3* there is also clustering at the level of the rat. (*C*) With two levels of clustering to correct for with a large ICC (58%) a large correction to confidence intervals is required. With the common test there is a highly significant difference comparing logAmp in HF and control (red bars). With the appropriate correction to confidence interval (green bars) it is clear there is no significant difference. There were 344 sparks from 17 cells from 7 control rats and 352 sparks from 22 cells from 5 HF rats.

LogAmp showed significant clustering both within each cell and within rats (*Table 3*, *Figure 5A, B*). *Figure 5C* shows the 95% confidence interval calculated, by the two approaches, for the difference in LogAmp between HF and control. The artificially small confidence interval produced by the common test makes the difference between the groups appear highly significant, but when the hierarchical data structure is taken into account, there is no significant difference.

Looking at the best-fitting analysis method (*Table 3*, defined by *χ*^2^-2LL criterion), it emerges that none of the spark variables is in fact significantly different between HF and control. LogAmp and LogFDHM appeared to be different between HF and control using the commonly used analysis approach, but this was because of an artefactually small standard error through failing to account for clustering.

## 4. Discussion

Different studies of Ca^2+^ fluxes in HF cells have given opposite results for aspects of Ca^2+^handling in isolated cardiomyocytes. The usual interpretation of this is that the direction of the effect may vary with choice of HF model and species. Our present study, however, shown in *Table 1* and Supplementary material online, *TablesS1–3* that even within a single HF model, studies already show opposite direction of effects.

Our study suggests that that these contradictory directions of effect could be a manifestation of the subtle but important defect in our conventional statistical analysis, whereby we assume that all the data points are independent with no significant clustering. In reality for many biological variables, repeated measurements (e.g. of sparks) from the same cell are more similar than those of other cells, and measurements from multiple cells of the same animal are in turn more similar than measurements from different animals. The consequence is when we have multiple measurements we have less meaningful data than the simple number of data points would suggest. This problem, the ability of large sample numbers to deceive us when we do not recognize clustering, has been previously identified in the field of neuroscience.^12^^,^^14^

While this study focuses on the clustering of transient data from different myocytes isolated from the same rat, as well as the clustering of spark data within each cell, this should not be seen as a reason to limit the use of hierarchical statistics to these settings. We have found significant clustering in other aspects of isolated myocyte properties, such as cell size.^22^ We have also found that these techniques are essential for robust analysis of other models. For example in a recent study where multiple cardiac slices were taken from each dog, significant clustering of slice contractility data meant that data were most appropriately analysed using hierarchical techniques.^23^ It would be safest, therefore, for the investigator to assume that any data with a hierarchical structure should be analysed in this way unless the hierarchical tests show non-significant clustering. We have made such testing freely available (below).

### 4.1 Hierarchical analysis exposes false positive findings

The transients of an individual cell are known to be almost identical and therefore, it is already conventional to recognize this clustering by treating the entirety of transient data from one cell as a single data point by averaging data from several transients.

Sparks, on the other hand, vary a great deal from one to the next within the same cell. This stochastic behavior results from the subcellular physiology of ryanodine receptors, which are present in variable configurations with variable opening probabilities and dynamics. The convention in our field has therefore been to not average spark data into a single value per cell, but consider each spark as a separate data point.

Our study indicates that the spark data show strong clustering at the cell level, which means that it is wrong to count the individual sparks for statistical purposes as though they were independent.

Strikingly, although the degree of clustering (ICC) varied from one variable to another, it was always significant for all of the variables. Moreover, even small values of the clustering measure (ICC) turn out to be surprisingly important. For example, spark logFDHM, which has an ICC of just 8.5%, changed its result from being clearly significant with the common analysis (*P* = 0.023), to being not at all significant (*P* = 0.778) with the hierarchical analysis. Many variables had far higher degrees of clustering (*Tables 2 and 3*), which means they are correspondingly even more susceptible to false positive results from the common analysis.

We were surprised to find that, after recognizing the clustering using hierarchical analysis, the only significant difference between HF and control is a higher Ca^2+^ transient amplitude. This is due to increased systolic Ca^2+^ without increase in diastolic Ca^2+^. If we had used standard *t*-tests alone the conclusion of this study would have been that Ca^2+^ transients in HF exhibited both higher amplitude and a longer time to peak and that these changes might result from sparks (the elemental subunits of transients) which were more prolonged and of greater amplitude, giving a mechanistic explanation for our finding of higher transient amplitude which is not supported by the data.

### 4.2 Cruder alternatives to hierarchical statistics—aggregation and disaggregation

Because hierarchical statistics are sometimes considered difficult to implement, some workers have tried the two alternative approaches.

Aggregation is the approach of lumping multiple data points into one, typically by averaging. This is already done routinely for Ca^2+^ transients at the cell level but not the rat level. The disadvantage is that applying this approach universally is excessively conservative, and would require very large studies to test hypotheses that could be tested more frugally if hierarchical statistics were used. For example, the 696 sparks in our study would have been collapsed into just 12 averages, one for each rat, if we had indiscriminately aggregated.

Results of aggregated statistical analysis in this study are shown in Supplementary material online, *TablesS4*, *S5* and are similar to the outcomes of the hierarchical tests but with overestimation of *P*-values and standard errors.

Disaggregation is the opposite approach, splitting the data so that each data point is considered to be independent. This is the commonest approach used in our field but has the scientific disadvantage that the statistics conducted assuming this independence give a falsely small standard error, falsely small confidence interval and therefore falsely small *P* value for the difference between groups.

An example of the catastrophic effect of disaggregation is that in our data the hierarchical model shows only 2 of 10 comparisons between HF and control to be statistically significant; in contrast the commonly-used approach (disaggregation of data) shows five comparisons to be statistically significant: a 150% overstatement.

A further challenge is that in the cellular laboratory there can be ‘good isolation days’ and ‘bad isolation days’. On good days large numbers of healthy cells are available for experimentation but on bad days smaller numbers of less healthy cells are available. If we aggregate, we give excessive weight to the likely unhealthier cells from the bad days. If we disaggregate, we give excessive weight to the likely healthier cells from the good days because they are more numerous.

The social sciences have long known of this problem and developed standardized approaches to avoid wasting resources pursuing false leads.^13^^,^^24^^,^^25^ We describe a similar problem in studies of isolated cardiac myocytes but we also describe a solution and provide cost-free simple tools which can be used to produce robust statistical results for comparisons involving clustered data.

### 4.3 Hierarchical tests are not merely a method of *P*-value adjustment

There is a general focus on the *P*-value as the only important outcome of significance testing in the biological literature.^26^ This approach leads to an overreliance on the apparent binary outcome of *P* < 0.05 vs. *P* > 0.05. In addition, the *P*-value gives the reader no indication of the magnitude of the effect and therefore its biological (rather than statistical) significance. An emphasis on point estimates and their precision (e.g. mean and 95% confidence interval) can prevent these issues.

If the hierarchical data structure is not considered, the 95% confidence intervals of an estimated mean are excessively narrow. Appropriate corrections can be made when performing hierarchical analysis. The magnitude of the required correction, as with other aspects of hierarchical testing, depends on the ICC. The estimated mean and 95% confidence intervals for each variable tested in this study, with and without hierarchical correction, are shown in Supplementary material online, *Tables S6* and *S7*.

### 4.4 A simple, cost-free, open source solution for statistical testing in cardiomyocyte studies

There are commercial software packages such as IBM SPSS Statistics, SAS (SAS Institute), STATA (StataCorp) which can do these hierarchical statistics very well. Readers can download the scripts which we show in Supplementary material online, Supplementary methods section. However, some readers may not have a particular commercial software package available or may be working with collaborators who do not.

We therefore present simple steps that any researcher can use without cost to perform these hierarchical statistics.

Step 1. Download the relevant files from Supplementary material online into a single working directory. These should include: *ratonly.Rmd, ratandcell.Rmd*, *Hierarchical Transient analysis with Rat-Level Clustering.xlsx*, and *Hierarchical Spark analysis Cell & Rat-Level Clustering.xlsx*.

Step 2. Modify the relevant Microsoft Excel file. If the data have the potential for clustering only at one level, for example, at the rat level, use the layout in Supplementary material online, *Hierarchical Transient analysis with Rat-Level Clustering.xlsx*. For data such as spark values, which has potential for an additional (e.g. cellular) level of clustering, use the layout in *Hierarchical Spark analysis Cell & Rat-Level Clustering.xlsx*.

Cells should be numbered sequentially even if they come from different rats, that is, if cells 1–10 come from rat 1, then cells from rat 2 should be numbered from 11 upwards and so on.

Column titles can be changed to reflect what your relevant hierarchical structure is—for example, dogs and heart slices or cell line and culture number.

Step 3. Download and install R for Windows, Mac, or Linux from https://www.r-project.org/ (14 August 2017, date last accessed).

Step 4. Download and install RStudio from https://www.rstudio.com/products/rstudio/download/ (14 August 2017, date last accessed)—this is a more user-friendly interface for R. Choose *RStudio Desktop—Open Source License*.

Step 5. Run RStudio and install the two extra packages we will need (‘lmerTest’ for the statistical tests and ‘readxl’ for reading in the Excel spreadsheets). This can be done using the ‘Tools → Install packages’ option in RStudio, and typing ‘lmerTest, readxl’ (first letter of lmer is lower case ‘L’) in the ‘Packages’ field, or by typing the following commands in the ‘Console’ window (R Console) and pressing Enter after each command:install.packages(‘lmerTest’)

install.packages(‘readxl’)

Step 6. Open the code corresponding to the Excel file you are working with in RStudio by clicking *File* menu and *Open File*. Open ratonly.Rmd if working with clustering at one level and ratandcell.Rmd if working with clustering at two levels.

Step 7. In the top right hand corner of the window in which the code has opened there is a *Run* command. Click this and then select *Run All*.

Step 8. Once the code has run, which may take several minutes, scroll down to the bottom of the same window where three tables (for ratonly.Rmd) or five tables (for ratandcell.Rmd). These tables show the following:

1. The first table shows the outcomes of the common test (*P* value and standard error for the difference), the degree of clustering (ICC), the equivalent outcomes for the hierarchical test, and whether the hierarchical test is a better fit for the data under the column ‘Superior fit’. If the *P* value in this column is < 0.05, there is sufficient clustering to make hierarchical techniques necessary. For ratandcell.Rmd, both cell-level hierarchical test (labelled group-level) and rat-and-cell level hierarchical test (labelled Parentgroup-group level) results are shown.
2. The second table shows the point estimate, that is, the estimated group mean when taking into account the hierarchical data structure, as well as the standard error and the lower and upper confidence intervals for each variable for each condition (HF and control).
3. The third table shows the pairwise comparisons. If there are only two conditions (e.g. HF and control) the *P*-values in this table are the same as the first table. This table becomes particularly relevant if there are >2 conditions since comparisons each pair of groups (A vs. B; B vs. C; A vs. C) may be desired. As with an ANOVA, these group-wise comparisons are only relevant if the overall test shown in the first table is significant.
4. The fourth and fifth tables are only output by ratandcell.Rmd and are the equivalent of the second and third tables but for the rat-and-cell level hierarchical test.

The scripts we provide are sufficient to perform the analyses in this paper. Once readers are comfortable doing this, they can extend the R script to allow more levels of clustering or to include other variables such as gender, animal weight, or cell size.

Moreover, these scripts are equally suited outside the cardiomyocyte arena and even to clinical data.

For users who do not have R or RStudio installed, but who wish to view the source code and sample output, we have supplied two supplementary HTML (ratonly.nb.html and ratandcell.nb.html) files which can be opened in any browser and serve as manuals to the analyses.

### 4.5 Limitations

Even though the hierarchical test describes the data much better (as shown by the *χ*^2^-2LL criterion), we should not assume that they are the ideal solution. It is merely a significant improvement for which we provide a straightforward method for implementation. Ideally researchers would engage with an expert statistician at an early phase of study preparation. Alternative models for performing hierarchical statistics are available including Generalized Estimating Equations (GEE), although we favour Mixed Models (with random coefficients) as this gives greater flexibility in the modelling process and is better at handling missing data.

Data transformations are frequently necessary, such as log transformation for the spark data which have a marked positive skew. In addition, even following valid transformation the meaning of the original data must be questioned. For example, some of the skewed distribution relating to variables describing spark morphology relates to the confocal line-scanning technique which is likely to miss the center of each spark, thus artefactually increasing the number of smaller sparks.^21^^,^^27^^,^^28^

## 5. Conclusions

The conventional approach used in our field to analyse cardiomyocyte data has a marked tendency to overstate the statistical significance of differences. This could be why some studies shows significant results in one direction and other studies show equally convincing significant results in the other direction.

We present simple steps that any researcher can implement to identify and allow for clustering, at the rat and cell level if need be, permitting them to use all their data points and yet obtain statistically valid results.

## Supplementary material

Supplementary material is available at *Cardiovascular Research* online.

## Supplementary Material

Supplementary Data

## Notes

Time for primary review: 44 days
