# Supplementary material for: Hierarchical statistical techniques are necessary to draw reliable conclusions from analysis of isolated cardiomyocyte studies
Source: Cardiovasc Res. 2017 Aug 30;113(14):1743–52. doi: 10.1093/cvr/cvx151 (PMC5852514; doi:10.1093/cvr/cvx151)
Supplement: Supplementary Data [file cvx151_suppl_data.zip › ratandcell.nb.html]

R Notebook - Rat and Cell-level hierarchical analysis


Code 

- Show All Code
- Hide All Code
- Download Rmd

# R Notebook - Rat and Cell-level hierarchical analysis

This is an R Markdown Notebook,

The source code has been separated into ‘chunks’ which can be run step-wise

---

How to use this script (Note green text indicates a comment rather than code)

1. Ensure the lmerTest package is installed; if unsure, type install.packages(‘lmerTest’); if using excel input files, you will also need to run install.packages(‘readxl’)
2. Set your working directory in your R program to the location of your input file (which does not necessarily need to be the location of this script) - In R studio this is done by going into ‘Session -> Set Working Directory’
3. Either ensure your input file is named “Hierarchical Transient analysis with Rat-Level Clustering.xlsx”, or replace the code on line 50 with the appropriate filename
4. Run this file using ‘Run -> Run all’

If readers would like other pragmatic examples in using mixed effect models for Hierarchical statistics, we would recommend https://www.jaredknowles.com/journal/2013/11/25/getting-started-with-mixed-effect-models-in-r

This is a PATCHED version of the original source code as of 07/08/2018 which works on the latest version of R and lmerTest.

Please contact james@jph.am for any questions

---

THE PROGRAM STARTS HERE

---

Here we load the packages we require for the analysis:


```
library(lmerTest) #If told 'there is no package called 'lmerTest', run 'install.packages("lmerTest")'
require(readxl) #If told 'there is no package called 'readxl', run 'install.packages("readxl")'
```


This prevents scientific notation for p values unless they are very small.


```
options(scipen=999)
```


Now we load the excel spreadsheet into the variable ‘input data’.


```
input_data <- read_excel("Hierarchical Spark analysis with Cell and Rat-Level Clustering.xlsx")
```


Here we rename the column of our condition (e.g. heart failure or not) to ‘Condition’, and the groups (e.g. cell and rat) to ‘Group’ and ‘ParentGroup’.

We then ensure that these are treated as categorical variables (factors) rather than continuous numerical values.

Finally, we create an empty results table.


```
names(input_data)[1] <- "Condition"
names(input_data)[2] <- "Group"
names(input_data)[3] <- "ParentGroup"
input_data[1] <- as.factor(unlist(input_data[1])) #Ensure the Condition group is treated as a categorical variable (distrinct groups) rather than continuous numerical values
input_data$ParentGroup <- factor(input_data$ParentGroup) #Ensure 'Parentgroup' (Rat) is treated as a factor
input_data$Group <- factor(input_data$Group) #Ensure 'Group' (Cell) is treated as a factor
df_output <- data.frame() #Create an empty results table
df_output_lsmeans <- data.frame() #Create an empty results table for least squared means (grouo-level analysis)
df_output_pairwise <- data.frame() #Create an empty results table for the pairway comparisons
df_output_lsmeans_2 <- data.frame() #Create an empty results table for least squared means (group- and parent-group level analysis)
df_output_pairwise_2 <- data.frame() #Create an empty results table for the pairway comparisons
```


This is the main ‘loop’ of the code, which runs once for each dependent variable in our spreadsheet.


```
for(dependent_variable in names(input_data[,4:ncol(input_data)])) { #This for loop ensures the indented code between the curly braces runs once for each column (dependent variable) from the 4th column onwards
  # STEP 1. Fit a NON-Hierarchical model (equivalent to a t-test)
  #Fit a model, calculate the standard error, p value, and -2 log Likelihood
  NON_hierarchical_model <- glm(get(dependent_variable) ~ Condition, data=input_data) #This fits a generlised linear model for each dependent variable and column 1 (HF_or_control; the presence or not of heart failure) and stores it in the variable named NON_hierarchical_model
  se_NON_hierarchical_model <- summary(NON_hierarchical_model)$coefficients[, 2][2] #This extracts the standard errors from the model and stores it in a new variable named se_NON_hierarchical_model
  p_NON_hierarchical_model <- summary(NON_hierarchical_model)$coefficients[, 4][2] #This extracts the standard errors from the model and stores it in a new variable named p_NON_hierarchical_model
  p_NON_hierarchical_model <- ifelse(p_NON_hierarchical_model<0.0001,"< 0.0001",round(p_NON_hierarchical_model,digits=4)) #If the p value calculated above is very small, we just replace it with "<0.0001"
  goodness_of_fit_NON_hierarchical_model <- logLik(NON_hierarchical_model)*-2 #We calculate a goodness of fit using the -2 logLikelihood of the model and save it in a new variable named goodness_of_fit_NON_hierarchical_model
  
  # STEP 2. Fit a Hierachical model at the CELL level
  # Step 2a. Fit a model, calculate the standard error, p value and -2 log Likelihood
  hierarchical_model <- lmer(get(dependent_variable) ~ Condition + (1|Group), REML=FALSE ,data=input_data) #We now fit a linear mixed effects (hierarchical) model and store that in the variable named hierarchical_model. We fit each dependent variable against column 1 ("HF_or_Control"), with 'Cell' as a group effect
  se_hierarchical_model <- summary(hierarchical_model)$coefficients[, 2][2] #As previously, extract the standard errors and store them in a variable
  p_hierarchical_model <- summary(hierarchical_model)$coefficients[ ,5][2] #As previously, extract the p values and store them in a variable
  p_hierarchical_model <- ifelse(p_hierarchical_model<0.0001,"< 0.0001",round(p_hierarchical_model,digits=4)) #If the p value calculated above is very small, we just replace it with "<0.0001"
  goodness_of_fit_hierarchical_model <- logLik(hierarchical_model)*-2 #As previously, calculate a goodness of fit and store it in a variable
  
  # STEP 2b. Calculate amount of clustering, defined as the intraclass correlation, a value between 0 and 1 
  table_of_variances <- as.data.frame(VarCorr(hierarchical_model)) #Create a dataframe (table) of the covariance parameters called 'table_of_variances'
  variance_of_means <- table_of_variances$vcov[1] #Extract the variance of the mean from the 'table_of_variances' and store it in a variable names 'variance_of_means'
  variance_of_individual_datapoints <- (table_of_variances$vcov[1] + table_of_variances$vcov[2]) #The total variance is also extracted, as defined as the variance of the cell-only model plus the residual variance
  icc <- variance_of_means / variance_of_individual_datapoints #The intra-class correlation (ICC) is calculated and defined by the variance of the model divided by the total variance
  
  # STEP 2c. Calculate goodness of fit and see if higher for hierarchical model
  improvement_in_goodness_of_fit <- goodness_of_fit_NON_hierarchical_model - goodness_of_fit_hierarchical_model #We calculate the difference between thepreviously-calculated goodness of fit for both the non-hierarchical and hierrachical models; this difference is stored in the variable improvement_in_goodness_of_fit
  betterfit <- 1-pchisq(improvement_in_goodness_of_fit[1],df=1) #The p value for whether the hierarchical model is a significant improvement is calculated using the Chi-squared distribution with 1 degree of freedom
  p_betterfit <- ifelse(betterfit<0.0001,"<0.0001",round(betterfit,digits=4)) #If the p value calculated above is very small, we just replace it with "<0.0001"
  superiorp <- ifelse(improvement_in_goodness_of_fit>0 & betterfit < 0.05,paste("Y (", p_betterfit ,")",sep=""),paste("N (", betterfit, ")",sep="")) #If there is an improvement in the goodness of fit and the p value is less than <0.05, we set the variable 'superiorp' to "Y", otherwise "N" followed by the value of the 'betterfit' variable, between brackets
  
  
  # STEP 3. Fit a Hierachical model at the CELL and RAT levels - this is merely a repeat of STEPS 2a, 2b and 2c, but with a different lmer() function in step 3a, where we supply both 'Rat' and 'Cell' columns as group-level variables. As Cells are 'nested' within Rats, we supply it in the formula + (1/Rat/Cell)
  # Step 3a. Fit a model, calculate the standard error, p value and -2 log Likelihood
  hierarchical_model_2 <- lmer(get(dependent_variable) ~ Condition + (1|ParentGroup/Group), REML=FALSE ,data=input_data) 
  se_hierarchical_model_2 <- summary(hierarchical_model_2)$coefficients[, 2][2]
  p_hierarchical_model_2 <- summary(hierarchical_model_2)$coefficients[ ,5][2]
  p_hierarchical_model_2 <- ifelse(p_hierarchical_model_2<0.0001,"< 0.0001",round(p_hierarchical_model_2,digits=4)) #If the p value calculated above is very small, we just replace it with "<0.0001"
  goodness_of_fit_hierarchical_model_2 <- logLik(hierarchical_model_2)*-2
  
  # STEP 3b. Calculate amount of clustering, defined as the intraclass correlation, a value between 0 and 1 
  table_of_variances_2 <- as.data.frame(VarCorr(hierarchical_model_2)) 
  variance_of_means_2 <- (table_of_variances_2$vcov[1] + table_of_variances_2$vcov[2])
  variance_of_individual_datapoints_2 <- (table_of_variances_2$vcov[1] + table_of_variances_2$vcov[2] + table_of_variances_2$vcov[3])
  icc_2 <- variance_of_means_2 / variance_of_individual_datapoints_2
  
  # STEP 3c. Calculate goodness of fit and see if higher for hierarchical model
  improvement_in_goodness_of_fit_2_vs_1 <- goodness_of_fit_hierarchical_model - goodness_of_fit_hierarchical_model_2
  betterfit_2_vs_1 <- 1-pchisq(improvement_in_goodness_of_fit_2_vs_1[1],df=1)
  p_betterfit_2 <- ifelse(betterfit_2_vs_1<0.0001,"<0.0001",round(betterfit_2_vs_1,digits=4))
  superiorp_2_vs_1 <- ifelse(improvement_in_goodness_of_fit>0 & betterfit_2_vs_1 < 0.05,paste("Y (", p_betterfit_2 ,")",sep=""),paste("N (", p_betterfit_2, ")",sep=""))
  
  # STEP 4a Calculate least squares means for each group of the outcome variables, and the pairwise comparisons - at group level only
  lsmean <- lsmeansLT(hierarchical_model) #We calculate the least squares means, standard errors and confidence intervals for the different outcomes (e.g. presence and absense of heart failure)
  row.names(lsmean) <- paste(dependent_variable,row.names(lsmean),sep=" - ") #Add the current variable in question to the row name
  difflsmean <- difflsmeans(hierarchical_model) #We test for significance between the different outcome levels
  row.names(difflsmean) <- paste(dependent_variable,row.names(difflsmean),sep=" - ") #Add the current variable in question to the row name
  
  # STEP 4b Do the same again, but with group and parentgroup-level analysis
  lsmean_2 <- lsmeansLT(hierarchical_model_2) #We calculate the least squares means, standard errors and confidence intervals for the different outcomes (e.g. presence and absense of heart failure)
  row.names(lsmean_2) <- paste(dependent_variable,row.names(lsmean_2),sep=" - ") #Add the current variable in question to the row name
  difflsmean_2 <- difflsmeans(hierarchical_model_2) #We test for significance between the different outcome levels
  row.names(difflsmean_2) <- paste(dependent_variable,row.names(difflsmean_2),sep=" - ") #Add the current variable in question to the row name
  
  df_output <- rbind( #We add the results to our results table in this function
    df_output,
    data.frame(
      CommonSE=round(se_NON_hierarchical_model,digits=3), #Add the standard error of the non-hierarchical model
      Commonp=ifelse(p_NON_hierarchical_model<0.0001,"< 0.0001",toString(round(p_NON_hierarchical_model,digits=4))), #Add the p value of the non-hierarchical model; if it's very small, replace it with "< 0.0001""
      ICC_cell=paste(round(icc*100,digits=1),"%",sep=""), #Add the intraclass correlation of the first hierarchical model (cell-level grouping)
      MixedSE_cell=round(se_hierarchical_model,digits=3), #Add the standard error of the first hierarchical model (cell-level grouping)
      Mixedp_cell=ifelse(p_hierarchical_model<0.0001,"< 0.0001",toString(round(p_hierarchical_model,digits=4))), #Add the p value of the hierarchical model; if it's very small, replace it with "< 0.0001""
      Superioryn=superiorp, #Add a column indicating if the first hierarchical model (cell-level grouping) is a statistically significantly better fit (Y or N) and the actual p value
      ICC_ratcell=paste(round(icc_2*100,digits=1),"%",sep=""), #Add the intraclass correlation of the second hierarchical model (rat and cell-level grouping)
      MixedSE_ratcell=round(se_hierarchical_model_2,digits=3), #Add the standard error of the second hierarchical model (rat and cell-level grouping)
      Mixedp_ratcell=ifelse(p_hierarchical_model_2<0.0001,"< 0.0001",toString(round(p_hierarchical_model_2,digits=4))), #Add the p value of the hierarchical model; if it's very small, replace it with "< 0.0001""
      Superioryn_2=superiorp_2_vs_1 #Add a column indicating if the second hierarchical model (rat and cell-level grouping) is a statistically significantly better fit (Y or N) and the actual p value
    )
  )
  
  df_output_lsmeans <- rbind( #We add the results to our ls squares results table in this function
    df_output_lsmeans,
    lsmean
  )
  
  df_output_pairwise <- rbind( #We add the results to our pairwise comparisons results table in this function
    df_output_pairwise,
    difflsmean
  )
  
  df_output_lsmeans_2 <- rbind( #We add the results to our ls squares results table in this function
    df_output_lsmeans_2,
    lsmean_2
  )
  
  df_output_pairwise_2 <- rbind( #We add the results to our pairwise comparisons results table in this function
    df_output_pairwise_2,
    difflsmean_2
  )
  
  row.names(df_output)[nrow(df_output)] <- dependent_variable #Set the name of the row to the dependent variable in questions
}
```


Finally, we specify the column headings here and print the table. You may need to scroll right in the table (using the arrow in the top right of the table) to visualise all 10 columns


```
names(df_output) = c("Common test (SE)","(p)","Group-level clustering (ICC)","Group-level (SE)","(p)","Superior fit (p)","Parentgroup-Group clustering (ICC)","Parentground-group (SE)","(p)","Superior fit vs. grou-level clustering (p)") #Set the column headings
print(df_output) #Output the summary table
```


Here we output a table containing the least squares means and confidence intervals for each outcome, grouped by each dependent variable, for the group-level analysis. You may need to scroll right in the table (using the arrow in the top right of the table) to visualise all 8 columns.


```
cols.dont.want <- c("DF", "t-value", "p-value")
df_output_lsmeans <- df_output_lsmeans[, ! names(df_output_lsmeans) %in% cols.dont.want, drop = F]
print(df_output_lsmeans) #Output the results table for LS means (group level analysis only)
```


```
                      Estimate Std. Error   df t value     lower     upper              Pr(>|t|)    
LogAmp - Condition0  -0.219245   0.023867 33.2 -9.1863 -0.267790 -0.170701       0.0000000001218 ***
LogAmp - Condition1  -0.104356   0.021373 34.8 -4.8827 -0.147752 -0.060960       0.0000230850611 ***
LogFWHM - Condition0  0.407808   0.013607 32.6 29.9696  0.380111  0.435506 < 0.00000000000000022 ***
LogFWHM - Condition1  0.412964   0.012477 34.7 33.0993  0.387628  0.438300 < 0.00000000000000022 ***
LogFDHM - Condition0  1.345823   0.021304 30.7 63.1724  1.302357  1.389289 < 0.00000000000000022 ***
LogFDHM - Condition1  1.337614   0.019501 32.5 68.5923  1.297915  1.377312 < 0.00000000000000022 ***
---
Signif. codes:  0 *** 0.001 ** 0.01 * 0.05 . 0.1   1
```


Here we output the pairwise comparisons for the group-level analysis The results are grouped for each output variable (left column), with each combination of outcomes undergoing significance testing. You may need to scroll right in the table (using the arrow in the top right of the table) to visualise all 7 columns.


```
cols.dont.want <- c("DF", "t-value", "Lower CI","Upper CI")
df_output_pairwise <- df_output_pairwise[, ! names(df_output_pairwise) %in% cols.dont.want, drop = F] #Remove the unwanted headings from the table
df_output_pairwise$`p-value` <- df_output_pairwise$`Pr(>|t|)` * ( nrow(df_output_pairwise) / length(names(input_data[,4:ncol(input_data)])) ) #Multiply the p values by the number of comparisons per dependent variable
names(df_output_pairwise)[names(df_output_pairwise)=="p-value"] <- "Bonferroni p-value" #Change the column heading
df_output_pairwise[df_output_pairwise$`Bonferroni p-value` > 1,"Bonferroni p-value"] <- 1 #Change any p value above 1 to 1
print(df_output_pairwise) #Output the results table for the pairwise comparisons
```


```
                                    Estimate Std. Error   df t value      lower      upper  Pr(>|t|) Bonferroni p-value   
LogAmp - Condition0 - Condition1  -0.1148895  0.0320375 33.9 -3.5861 -0.1800026 -0.0497764 0.0010434           0.001043 **
LogFWHM - Condition0 - Condition1 -0.0051554  0.0184614 33.5 -0.2792 -0.0426925  0.0323817 0.7817678           0.781768   
LogFDHM - Condition0 - Condition1  0.0082091  0.0288816 31.5  0.2842 -0.0506567  0.0670749 0.7780916           0.778092   
---
Signif. codes:  0 *** 0.001 ** 0.01 * 0.05 . 0.1   1
```


Here we output a table containing the least squares means and confidence intervals for each outcome, grouped by each dependent variable, for the parentgroup-group-level analysis. You may need to scroll right in the table (using the arrow in the top right of the table) to visualise all 8 columns.


```
cols.dont.want <- c("DF", "t-value", "p-value")
df_output_lsmeans_2 <- df_output_lsmeans_2[, ! names(df_output_lsmeans_2) %in% cols.dont.want, drop = F]
print(df_output_lsmeans_2) #Output the results table for LS means (group level analysis only)
```


```
                       Estimate Std. Error   df t value      lower      upper              Pr(>|t|)    
LogAmp - Condition0  -0.1952993  0.0428872  9.1 -4.5538 -0.2921650 -0.0984336              0.001342 ** 
LogAmp - Condition1  -0.1137883  0.0476393  7.1 -2.3885 -0.2259750 -0.0016015              0.047566 *  
LogFWHM - Condition0  0.4078084  0.0136074 32.6 29.9696  0.3801108  0.4355060 < 0.00000000000000022 ***
LogFWHM - Condition1  0.4129638  0.0124765 34.7 33.0993  0.3876278  0.4382997 < 0.00000000000000022 ***
LogFDHM - Condition0  1.3389152  0.0259005  8.7 51.6946  1.2799794  1.3978510     0.000000000004253 ***
LogFDHM - Condition1  1.3328168  0.0245965  5.9 54.1873  1.2723322  1.3933015     0.000000003659326 ***
---
Signif. codes:  0 *** 0.001 ** 0.01 * 0.05 . 0.1   1
```


Here we output the pairwise comparisons for the parentgroup-group-level analysis The results are grouped for each output variable (left column), with each combination of outcomes undergoing significance testing. You may need to scroll right in the table (using the arrow in the top right of the table) to visualise all 7 columns.


```
cols.dont.want <- c("DF", "t-value", "Lower CI","Upper CI")
df_output_pairwise_2 <- df_output_pairwise_2[, ! names(df_output_pairwise_2) %in% cols.dont.want, drop = F] #Remove the unwanted headings from the table
df_output_pairwise_2$`p-value` <- df_output_pairwise_2$`Pr(>|t|)` * ( nrow(df_output_pairwise_2) / length(names(input_data[,4:ncol(input_data)])) ) #Multiply the p values by the number of comparisons per dependent variable
names(df_output_pairwise_2)[names(df_output_pairwise_2)=="p-value"] <- "Bonferroni p-value" #Change the column heading
df_output_pairwise_2[df_output_pairwise_2$`Bonferroni p-value` > 1,"Bonferroni p-value"] <- 1 #Change any p value above 1 to 1
print(df_output_pairwise_2) #Output the results table for the pairwise comparisons  (group level analysis only)
```


```
                                    Estimate Std. Error   df t value      lower      upper Pr(>|t|) Bonferroni p-value
LogAmp - Condition0 - Condition1  -0.0815110  0.0641000  7.9 -1.2716 -0.2295304  0.0665083  0.23950             0.2395
LogFWHM - Condition0 - Condition1 -0.0051554  0.0184614 33.5 -0.2792 -0.0426924  0.0323817  0.78177             0.7818
LogFDHM - Condition0 - Condition1  0.0060984  0.0357186  7.2  0.1707 -0.0779909  0.0901876  0.86916             0.8692
```


LS0tDQp0aXRsZTogIlIgTm90ZWJvb2sgLSBSYXQgYW5kIENlbGwtbGV2ZWwgaGllcmFyY2hpY2FsIGFuYWx5c2lzIg0Kb3V0cHV0OiBodG1sX25vdGVib29rDQotLS0NCg0KVGhpcyBpcyBhbiBbUiBNYXJrZG93bl0oaHR0cDovL3JtYXJrZG93bi5yc3R1ZGlvLmNvbSkgTm90ZWJvb2ssIA0KDQpUaGUgc291cmNlIGNvZGUgaGFzIGJlZW4gc2VwYXJhdGVkIGludG8gJ2NodW5rcycgd2hpY2ggY2FuIGJlIHJ1biBzdGVwLXdpc2UNCg0KLS0tDQoNCkhvdyB0byB1c2UgdGhpcyBzY3JpcHQgKE5vdGUgZ3JlZW4gdGV4dCBpbmRpY2F0ZXMgYSBjb21tZW50IHJhdGhlciB0aGFuIGNvZGUpDQogDQoxLiBFbnN1cmUgdGhlIGxtZXJUZXN0IHBhY2thZ2UgaXMgaW5zdGFsbGVkOyBpZiB1bnN1cmUsIHR5cGUgaW5zdGFsbC5wYWNrYWdlcygnbG1lclRlc3QnKTsgaWYgdXNpbmcgZXhjZWwgaW5wdXQgZmlsZXMsIHlvdSB3aWxsIGFsc28gbmVlZCB0byBydW4gaW5zdGFsbC5wYWNrYWdlcygncmVhZHhsJykNCjIuIFNldCB5b3VyIHdvcmtpbmcgZGlyZWN0b3J5IGluIHlvdXIgUiBwcm9ncmFtIHRvIHRoZSBsb2NhdGlvbiBvZiB5b3VyIGlucHV0IGZpbGUgKHdoaWNoIGRvZXMgbm90IG5lY2Vzc2FyaWx5IG5lZWQgdG8gYmUgdGhlIGxvY2F0aW9uIG9mIHRoaXMgc2NyaXB0KSAtIEluIFIgc3R1ZGlvIHRoaXMgaXMgZG9uZSBieSBnb2luZyBpbnRvICdTZXNzaW9uIC0+IFNldCBXb3JraW5nIERpcmVjdG9yeScNCjMuIEVpdGhlciBlbnN1cmUgeW91ciBpbnB1dCBmaWxlIGlzIG5hbWVkICJIaWVyYXJjaGljYWwgVHJhbnNpZW50IGFuYWx5c2lzIHdpdGggUmF0LUxldmVsIENsdXN0ZXJpbmcueGxzeCIsIG9yIHJlcGxhY2UgdGhlIGNvZGUgb24gbGluZSA1MCB3aXRoIHRoZSBhcHByb3ByaWF0ZSBmaWxlbmFtZQ0KNC4gUnVuIHRoaXMgZmlsZSB1c2luZyAnUnVuIC0+IFJ1biBhbGwnDQoNCklmIHJlYWRlcnMgd291bGQgbGlrZSBvdGhlciBwcmFnbWF0aWMgZXhhbXBsZXMgaW4gdXNpbmcgbWl4ZWQgZWZmZWN0IG1vZGVscyBmb3IgSGllcmFyY2hpY2FsIHN0YXRpc3RpY3MsIHdlIHdvdWxkIHJlY29tbWVuZCBodHRwczovL3d3dy5qYXJlZGtub3dsZXMuY29tL2pvdXJuYWwvMjAxMy8xMS8yNS9nZXR0aW5nLXN0YXJ0ZWQtd2l0aC1taXhlZC1lZmZlY3QtbW9kZWxzLWluLXINCg0KVGhpcyBpcyBhIFBBVENIRUQgdmVyc2lvbiBvZiB0aGUgb3JpZ2luYWwgc291cmNlIGNvZGUgYXMgb2YgMDcvMDgvMjAxOCB3aGljaCB3b3JrcyBvbiB0aGUgbGF0ZXN0IHZlcnNpb24gb2YgUiBhbmQgbG1lclRlc3QuDQogDQpQbGVhc2UgY29udGFjdCBqYW1lc0BqcGguYW0gZm9yIGFueSBxdWVzdGlvbnMNCg0KLS0tDQoNClRIRSBQUk9HUkFNIFNUQVJUUyBIRVJFDQoNCi0tLQ0KDQpIZXJlIHdlIGxvYWQgdGhlIHBhY2thZ2VzIHdlIHJlcXVpcmUgZm9yIHRoZSBhbmFseXNpczoNCg0KYGBge3J9DQpsaWJyYXJ5KGxtZXJUZXN0KSAjSWYgdG9sZCAndGhlcmUgaXMgbm8gcGFja2FnZSBjYWxsZWQgJ2xtZXJUZXN0JywgcnVuICdpbnN0YWxsLnBhY2thZ2VzKCJsbWVyVGVzdCIpJw0KcmVxdWlyZShyZWFkeGwpICNJZiB0b2xkICd0aGVyZSBpcyBubyBwYWNrYWdlIGNhbGxlZCAncmVhZHhsJywgcnVuICdpbnN0YWxsLnBhY2thZ2VzKCJyZWFkeGwiKScNCmBgYA0KDQoNCg0KVGhpcyBwcmV2ZW50cyBzY2llbnRpZmljIG5vdGF0aW9uIGZvciBwIHZhbHVlcyB1bmxlc3MgdGhleSBhcmUgdmVyeSBzbWFsbC4NCg0KYGBge3J9DQpvcHRpb25zKHNjaXBlbj05OTkpIA0KYGBgDQoNCk5vdyB3ZSBsb2FkIHRoZSBleGNlbCBzcHJlYWRzaGVldCBpbnRvIHRoZSB2YXJpYWJsZSAnaW5wdXQgZGF0YScuDQoNCmBgYHtyfQ0KaW5wdXRfZGF0YSA8LSByZWFkX2V4Y2VsKCJIaWVyYXJjaGljYWwgU3BhcmsgYW5hbHlzaXMgd2l0aCBDZWxsIGFuZCBSYXQtTGV2ZWwgQ2x1c3RlcmluZy54bHN4IikgDQpgYGANCg0KSGVyZSB3ZSByZW5hbWUgdGhlIGNvbHVtbiBvZiBvdXIgY29uZGl0aW9uIChlLmcuIGhlYXJ0IGZhaWx1cmUgb3Igbm90KSB0byAnQ29uZGl0aW9uJywgYW5kIHRoZSBncm91cHMgKGUuZy4gY2VsbCBhbmQgcmF0KSB0byAnR3JvdXAnIGFuZCAnUGFyZW50R3JvdXAnLg0KDQpXZSB0aGVuIGVuc3VyZSB0aGF0IHRoZXNlIGFyZSB0cmVhdGVkIGFzIGNhdGVnb3JpY2FsIHZhcmlhYmxlcyAoZmFjdG9ycykgcmF0aGVyIHRoYW4gY29udGludW91cyBudW1lcmljYWwgdmFsdWVzLg0KDQpGaW5hbGx5LCB3ZSBjcmVhdGUgYW4gZW1wdHkgcmVzdWx0cyB0YWJsZS4NCg0KYGBge3J9DQpuYW1lcyhpbnB1dF9kYXRhKVsxXSA8LSAiQ29uZGl0aW9uIg0KbmFtZXMoaW5wdXRfZGF0YSlbMl0gPC0gIkdyb3VwIg0KbmFtZXMoaW5wdXRfZGF0YSlbM10gPC0gIlBhcmVudEdyb3VwIg0KDQppbnB1dF9kYXRhWzFdIDwtIGFzLmZhY3Rvcih1bmxpc3QoaW5wdXRfZGF0YVsxXSkpICNFbnN1cmUgdGhlIENvbmRpdGlvbiBncm91cCBpcyB0cmVhdGVkIGFzIGEgY2F0ZWdvcmljYWwgdmFyaWFibGUgKGRpc3RyaW5jdCBncm91cHMpIHJhdGhlciB0aGFuIGNvbnRpbnVvdXMgbnVtZXJpY2FsIHZhbHVlcw0KaW5wdXRfZGF0YSRQYXJlbnRHcm91cCA8LSBmYWN0b3IoaW5wdXRfZGF0YSRQYXJlbnRHcm91cCkgI0Vuc3VyZSAnUGFyZW50Z3JvdXAnIChSYXQpIGlzIHRyZWF0ZWQgYXMgYSBmYWN0b3INCmlucHV0X2RhdGEkR3JvdXAgPC0gZmFjdG9yKGlucHV0X2RhdGEkR3JvdXApICNFbnN1cmUgJ0dyb3VwJyAoQ2VsbCkgaXMgdHJlYXRlZCBhcyBhIGZhY3Rvcg0KDQpkZl9vdXRwdXQgPC0gZGF0YS5mcmFtZSgpICNDcmVhdGUgYW4gZW1wdHkgcmVzdWx0cyB0YWJsZQ0KZGZfb3V0cHV0X2xzbWVhbnMgPC0gZGF0YS5mcmFtZSgpICNDcmVhdGUgYW4gZW1wdHkgcmVzdWx0cyB0YWJsZSBmb3IgbGVhc3Qgc3F1YXJlZCBtZWFucyAoZ3JvdW8tbGV2ZWwgYW5hbHlzaXMpDQpkZl9vdXRwdXRfcGFpcndpc2UgPC0gZGF0YS5mcmFtZSgpICNDcmVhdGUgYW4gZW1wdHkgcmVzdWx0cyB0YWJsZSBmb3IgdGhlIHBhaXJ3YXkgY29tcGFyaXNvbnMNCmRmX291dHB1dF9sc21lYW5zXzIgPC0gZGF0YS5mcmFtZSgpICNDcmVhdGUgYW4gZW1wdHkgcmVzdWx0cyB0YWJsZSBmb3IgbGVhc3Qgc3F1YXJlZCBtZWFucyAoZ3JvdXAtIGFuZCBwYXJlbnQtZ3JvdXAgbGV2ZWwgYW5hbHlzaXMpDQpkZl9vdXRwdXRfcGFpcndpc2VfMiA8LSBkYXRhLmZyYW1lKCkgI0NyZWF0ZSBhbiBlbXB0eSByZXN1bHRzIHRhYmxlIGZvciB0aGUgcGFpcndheSBjb21wYXJpc29ucw0KYGBgDQoNClRoaXMgaXMgdGhlIG1haW4gJ2xvb3AnIG9mIHRoZSBjb2RlLCB3aGljaCBydW5zIG9uY2UgZm9yIGVhY2ggZGVwZW5kZW50IHZhcmlhYmxlIGluIG91ciBzcHJlYWRzaGVldC4NCg0KYGBge3J9DQpmb3IoZGVwZW5kZW50X3ZhcmlhYmxlIGluIG5hbWVzKGlucHV0X2RhdGFbLDQ6bmNvbChpbnB1dF9kYXRhKV0pKSB7ICNUaGlzIGZvciBsb29wIGVuc3VyZXMgdGhlIGluZGVudGVkIGNvZGUgYmV0d2VlbiB0aGUgY3VybHkgYnJhY2VzIHJ1bnMgb25jZSBmb3IgZWFjaCBjb2x1bW4gKGRlcGVuZGVudCB2YXJpYWJsZSkgZnJvbSB0aGUgNHRoIGNvbHVtbiBvbndhcmRzDQogICMgU1RFUCAxLiBGaXQgYSBOT04tSGllcmFyY2hpY2FsIG1vZGVsIChlcXVpdmFsZW50IHRvIGEgdC10ZXN0KQ0KICAjRml0IGEgbW9kZWwsIGNhbGN1bGF0ZSB0aGUgc3RhbmRhcmQgZXJyb3IsIHAgdmFsdWUsIGFuZCAtMiBsb2cgTGlrZWxpaG9vZA0KICBOT05faGllcmFyY2hpY2FsX21vZGVsIDwtIGdsbShnZXQoZGVwZW5kZW50X3ZhcmlhYmxlKSB+IENvbmRpdGlvbiwgZGF0YT1pbnB1dF9kYXRhKSAjVGhpcyBmaXRzIGEgZ2VuZXJsaXNlZCBsaW5lYXIgbW9kZWwgZm9yIGVhY2ggZGVwZW5kZW50IHZhcmlhYmxlIGFuZCBjb2x1bW4gMSAoSEZfb3JfY29udHJvbDsgdGhlIHByZXNlbmNlIG9yIG5vdCBvZiBoZWFydCBmYWlsdXJlKSBhbmQgc3RvcmVzIGl0IGluIHRoZSB2YXJpYWJsZSBuYW1lZCBOT05faGllcmFyY2hpY2FsX21vZGVsDQogIHNlX05PTl9oaWVyYXJjaGljYWxfbW9kZWwgPC0gc3VtbWFyeShOT05faGllcmFyY2hpY2FsX21vZGVsKSRjb2VmZmljaWVudHNbLCAyXVsyXSAjVGhpcyBleHRyYWN0cyB0aGUgc3RhbmRhcmQgZXJyb3JzIGZyb20gdGhlIG1vZGVsIGFuZCBzdG9yZXMgaXQgaW4gYSBuZXcgdmFyaWFibGUgbmFtZWQgc2VfTk9OX2hpZXJhcmNoaWNhbF9tb2RlbA0KICBwX05PTl9oaWVyYXJjaGljYWxfbW9kZWwgPC0gc3VtbWFyeShOT05faGllcmFyY2hpY2FsX21vZGVsKSRjb2VmZmljaWVudHNbLCA0XVsyXSAjVGhpcyBleHRyYWN0cyB0aGUgc3RhbmRhcmQgZXJyb3JzIGZyb20gdGhlIG1vZGVsIGFuZCBzdG9yZXMgaXQgaW4gYSBuZXcgdmFyaWFibGUgbmFtZWQgcF9OT05faGllcmFyY2hpY2FsX21vZGVsDQogIHBfTk9OX2hpZXJhcmNoaWNhbF9tb2RlbCA8LSBpZmVsc2UocF9OT05faGllcmFyY2hpY2FsX21vZGVsPDAuMDAwMSwiPCAwLjAwMDEiLHJvdW5kKHBfTk9OX2hpZXJhcmNoaWNhbF9tb2RlbCxkaWdpdHM9NCkpICNJZiB0aGUgcCB2YWx1ZSBjYWxjdWxhdGVkIGFib3ZlIGlzIHZlcnkgc21hbGwsIHdlIGp1c3QgcmVwbGFjZSBpdCB3aXRoICI8MC4wMDAxIg0KICBnb29kbmVzc19vZl9maXRfTk9OX2hpZXJhcmNoaWNhbF9tb2RlbCA8LSBsb2dMaWsoTk9OX2hpZXJhcmNoaWNhbF9tb2RlbCkqLTIgI1dlIGNhbGN1bGF0ZSBhIGdvb2RuZXNzIG9mIGZpdCB1c2luZyB0aGUgLTIgbG9nTGlrZWxpaG9vZCBvZiB0aGUgbW9kZWwgYW5kIHNhdmUgaXQgaW4gYSBuZXcgdmFyaWFibGUgbmFtZWQgZ29vZG5lc3Nfb2ZfZml0X05PTl9oaWVyYXJjaGljYWxfbW9kZWwNCiAgDQogICMgU1RFUCAyLiBGaXQgYSBIaWVyYWNoaWNhbCBtb2RlbCBhdCB0aGUgQ0VMTCBsZXZlbA0KICAjIFN0ZXAgMmEuIEZpdCBhIG1vZGVsLCBjYWxjdWxhdGUgdGhlIHN0YW5kYXJkIGVycm9yLCBwIHZhbHVlIGFuZCAtMiBsb2cgTGlrZWxpaG9vZA0KICBoaWVyYXJjaGljYWxfbW9kZWwgPC0gbG1lcihnZXQoZGVwZW5kZW50X3ZhcmlhYmxlKSB+IENvbmRpdGlvbiArICgxfEdyb3VwKSwgUkVNTD1GQUxTRSAsZGF0YT1pbnB1dF9kYXRhKSAjV2Ugbm93IGZpdCBhIGxpbmVhciBtaXhlZCBlZmZlY3RzIChoaWVyYXJjaGljYWwpIG1vZGVsIGFuZCBzdG9yZSB0aGF0IGluIHRoZSB2YXJpYWJsZSBuYW1lZCBoaWVyYXJjaGljYWxfbW9kZWwuIFdlIGZpdCBlYWNoIGRlcGVuZGVudCB2YXJpYWJsZSBhZ2FpbnN0IGNvbHVtbiAxICgiSEZfb3JfQ29udHJvbCIpLCB3aXRoICdDZWxsJyBhcyBhIGdyb3VwIGVmZmVjdA0KICBzZV9oaWVyYXJjaGljYWxfbW9kZWwgPC0gc3VtbWFyeShoaWVyYXJjaGljYWxfbW9kZWwpJGNvZWZmaWNpZW50c1ssIDJdWzJdICNBcyBwcmV2aW91c2x5LCBleHRyYWN0IHRoZSBzdGFuZGFyZCBlcnJvcnMgYW5kIHN0b3JlIHRoZW0gaW4gYSB2YXJpYWJsZQ0KICBwX2hpZXJhcmNoaWNhbF9tb2RlbCA8LSBzdW1tYXJ5KGhpZXJhcmNoaWNhbF9tb2RlbCkkY29lZmZpY2llbnRzWyAsNV1bMl0gI0FzIHByZXZpb3VzbHksIGV4dHJhY3QgdGhlIHAgdmFsdWVzIGFuZCBzdG9yZSB0aGVtIGluIGEgdmFyaWFibGUNCiAgcF9oaWVyYXJjaGljYWxfbW9kZWwgPC0gaWZlbHNlKHBfaGllcmFyY2hpY2FsX21vZGVsPDAuMDAwMSwiPCAwLjAwMDEiLHJvdW5kKHBfaGllcmFyY2hpY2FsX21vZGVsLGRpZ2l0cz00KSkgI0lmIHRoZSBwIHZhbHVlIGNhbGN1bGF0ZWQgYWJvdmUgaXMgdmVyeSBzbWFsbCwgd2UganVzdCByZXBsYWNlIGl0IHdpdGggIjwwLjAwMDEiDQogIGdvb2RuZXNzX29mX2ZpdF9oaWVyYXJjaGljYWxfbW9kZWwgPC0gbG9nTGlrKGhpZXJhcmNoaWNhbF9tb2RlbCkqLTIgI0FzIHByZXZpb3VzbHksIGNhbGN1bGF0ZSBhIGdvb2RuZXNzIG9mIGZpdCBhbmQgc3RvcmUgaXQgaW4gYSB2YXJpYWJsZQ0KICANCiAgIyBTVEVQIDJiLiBDYWxjdWxhdGUgYW1vdW50IG9mIGNsdXN0ZXJpbmcsIGRlZmluZWQgYXMgdGhlIGludHJhY2xhc3MgY29ycmVsYXRpb24sIGEgdmFsdWUgYmV0d2VlbiAwIGFuZCAxIA0KICB0YWJsZV9vZl92YXJpYW5jZXMgPC0gYXMuZGF0YS5mcmFtZShWYXJDb3JyKGhpZXJhcmNoaWNhbF9tb2RlbCkpICNDcmVhdGUgYSBkYXRhZnJhbWUgKHRhYmxlKSBvZiB0aGUgY292YXJpYW5jZSBwYXJhbWV0ZXJzIGNhbGxlZCAndGFibGVfb2ZfdmFyaWFuY2VzJw0KICB2YXJpYW5jZV9vZl9tZWFucyA8LSB0YWJsZV9vZl92YXJpYW5jZXMkdmNvdlsxXSAjRXh0cmFjdCB0aGUgdmFyaWFuY2Ugb2YgdGhlIG1lYW4gZnJvbSB0aGUgJ3RhYmxlX29mX3ZhcmlhbmNlcycgYW5kIHN0b3JlIGl0IGluIGEgdmFyaWFibGUgbmFtZXMgJ3ZhcmlhbmNlX29mX21lYW5zJw0KICB2YXJpYW5jZV9vZl9pbmRpdmlkdWFsX2RhdGFwb2ludHMgPC0gKHRhYmxlX29mX3ZhcmlhbmNlcyR2Y292WzFdICsgdGFibGVfb2ZfdmFyaWFuY2VzJHZjb3ZbMl0pICNUaGUgdG90YWwgdmFyaWFuY2UgaXMgYWxzbyBleHRyYWN0ZWQsIGFzIGRlZmluZWQgYXMgdGhlIHZhcmlhbmNlIG9mIHRoZSBjZWxsLW9ubHkgbW9kZWwgcGx1cyB0aGUgcmVzaWR1YWwgdmFyaWFuY2UNCiAgaWNjIDwtIHZhcmlhbmNlX29mX21lYW5zIC8gdmFyaWFuY2Vfb2ZfaW5kaXZpZHVhbF9kYXRhcG9pbnRzICNUaGUgaW50cmEtY2xhc3MgY29ycmVsYXRpb24gKElDQykgaXMgY2FsY3VsYXRlZCBhbmQgZGVmaW5lZCBieSB0aGUgdmFyaWFuY2Ugb2YgdGhlIG1vZGVsIGRpdmlkZWQgYnkgdGhlIHRvdGFsIHZhcmlhbmNlDQogIA0KICAjIFNURVAgMmMuIENhbGN1bGF0ZSBnb29kbmVzcyBvZiBmaXQgYW5kIHNlZSBpZiBoaWdoZXIgZm9yIGhpZXJhcmNoaWNhbCBtb2RlbA0KICBpbXByb3ZlbWVudF9pbl9nb29kbmVzc19vZl9maXQgPC0gZ29vZG5lc3Nfb2ZfZml0X05PTl9oaWVyYXJjaGljYWxfbW9kZWwgLSBnb29kbmVzc19vZl9maXRfaGllcmFyY2hpY2FsX21vZGVsICNXZSBjYWxjdWxhdGUgdGhlIGRpZmZlcmVuY2UgYmV0d2VlbiB0aGVwcmV2aW91c2x5LWNhbGN1bGF0ZWQgZ29vZG5lc3Mgb2YgZml0IGZvciBib3RoIHRoZSBub24taGllcmFyY2hpY2FsIGFuZCBoaWVycmFjaGljYWwgbW9kZWxzOyB0aGlzIGRpZmZlcmVuY2UgaXMgc3RvcmVkIGluIHRoZSB2YXJpYWJsZSBpbXByb3ZlbWVudF9pbl9nb29kbmVzc19vZl9maXQNCiAgYmV0dGVyZml0IDwtIDEtcGNoaXNxKGltcHJvdmVtZW50X2luX2dvb2RuZXNzX29mX2ZpdFsxXSxkZj0xKSAjVGhlIHAgdmFsdWUgZm9yIHdoZXRoZXIgdGhlIGhpZXJhcmNoaWNhbCBtb2RlbCBpcyBhIHNpZ25pZmljYW50IGltcHJvdmVtZW50IGlzIGNhbGN1bGF0ZWQgdXNpbmcgdGhlIENoaS1zcXVhcmVkIGRpc3RyaWJ1dGlvbiB3aXRoIDEgZGVncmVlIG9mIGZyZWVkb20NCiAgcF9iZXR0ZXJmaXQgPC0gaWZlbHNlKGJldHRlcmZpdDwwLjAwMDEsIjwwLjAwMDEiLHJvdW5kKGJldHRlcmZpdCxkaWdpdHM9NCkpICNJZiB0aGUgcCB2YWx1ZSBjYWxjdWxhdGVkIGFib3ZlIGlzIHZlcnkgc21hbGwsIHdlIGp1c3QgcmVwbGFjZSBpdCB3aXRoICI8MC4wMDAxIg0KICBzdXBlcmlvcnAgPC0gaWZlbHNlKGltcHJvdmVtZW50X2luX2dvb2RuZXNzX29mX2ZpdD4wICYgYmV0dGVyZml0IDwgMC4wNSxwYXN0ZSgiWSAoIiwgcF9iZXR0ZXJmaXQgLCIpIixzZXA9IiIpLHBhc3RlKCJOICgiLCBiZXR0ZXJmaXQsICIpIixzZXA9IiIpKSAjSWYgdGhlcmUgaXMgYW4gaW1wcm92ZW1lbnQgaW4gdGhlIGdvb2RuZXNzIG9mIGZpdCBhbmQgdGhlIHAgdmFsdWUgaXMgbGVzcyB0aGFuIDwwLjA1LCB3ZSBzZXQgdGhlIHZhcmlhYmxlICdzdXBlcmlvcnAnIHRvICJZIiwgb3RoZXJ3aXNlICJOIiBmb2xsb3dlZCBieSB0aGUgdmFsdWUgb2YgdGhlICdiZXR0ZXJmaXQnIHZhcmlhYmxlLCBiZXR3ZWVuIGJyYWNrZXRzDQogIA0KICANCiAgIyBTVEVQIDMuIEZpdCBhIEhpZXJhY2hpY2FsIG1vZGVsIGF0IHRoZSBDRUxMIGFuZCBSQVQgbGV2ZWxzIC0gdGhpcyBpcyBtZXJlbHkgYSByZXBlYXQgb2YgU1RFUFMgMmEsIDJiIGFuZCAyYywgYnV0IHdpdGggYSBkaWZmZXJlbnQgbG1lcigpIGZ1bmN0aW9uIGluIHN0ZXAgM2EsIHdoZXJlIHdlIHN1cHBseSBib3RoICdSYXQnIGFuZCAnQ2VsbCcgY29sdW1ucyBhcyBncm91cC1sZXZlbCB2YXJpYWJsZXMuIEFzIENlbGxzIGFyZSAnbmVzdGVkJyB3aXRoaW4gUmF0cywgd2Ugc3VwcGx5IGl0IGluIHRoZSBmb3JtdWxhICsgKDEvUmF0L0NlbGwpDQogICMgU3RlcCAzYS4gRml0IGEgbW9kZWwsIGNhbGN1bGF0ZSB0aGUgc3RhbmRhcmQgZXJyb3IsIHAgdmFsdWUgYW5kIC0yIGxvZyBMaWtlbGlob29kDQogIGhpZXJhcmNoaWNhbF9tb2RlbF8yIDwtIGxtZXIoZ2V0KGRlcGVuZGVudF92YXJpYWJsZSkgfiBDb25kaXRpb24gKyAoMXxQYXJlbnRHcm91cC9Hcm91cCksIFJFTUw9RkFMU0UgLGRhdGE9aW5wdXRfZGF0YSkgDQogIHNlX2hpZXJhcmNoaWNhbF9tb2RlbF8yIDwtIHN1bW1hcnkoaGllcmFyY2hpY2FsX21vZGVsXzIpJGNvZWZmaWNpZW50c1ssIDJdWzJdDQogIHBfaGllcmFyY2hpY2FsX21vZGVsXzIgPC0gc3VtbWFyeShoaWVyYXJjaGljYWxfbW9kZWxfMikkY29lZmZpY2llbnRzWyAsNV1bMl0NCiAgcF9oaWVyYXJjaGljYWxfbW9kZWxfMiA8LSBpZmVsc2UocF9oaWVyYXJjaGljYWxfbW9kZWxfMjwwLjAwMDEsIjwgMC4wMDAxIixyb3VuZChwX2hpZXJhcmNoaWNhbF9tb2RlbF8yLGRpZ2l0cz00KSkgI0lmIHRoZSBwIHZhbHVlIGNhbGN1bGF0ZWQgYWJvdmUgaXMgdmVyeSBzbWFsbCwgd2UganVzdCByZXBsYWNlIGl0IHdpdGggIjwwLjAwMDEiDQogIGdvb2RuZXNzX29mX2ZpdF9oaWVyYXJjaGljYWxfbW9kZWxfMiA8LSBsb2dMaWsoaGllcmFyY2hpY2FsX21vZGVsXzIpKi0yDQogIA0KICAjIFNURVAgM2IuIENhbGN1bGF0ZSBhbW91bnQgb2YgY2x1c3RlcmluZywgZGVmaW5lZCBhcyB0aGUgaW50cmFjbGFzcyBjb3JyZWxhdGlvbiwgYSB2YWx1ZSBiZXR3ZWVuIDAgYW5kIDEgDQogIHRhYmxlX29mX3ZhcmlhbmNlc18yIDwtIGFzLmRhdGEuZnJhbWUoVmFyQ29ycihoaWVyYXJjaGljYWxfbW9kZWxfMikpIA0KICB2YXJpYW5jZV9vZl9tZWFuc18yIDwtICh0YWJsZV9vZl92YXJpYW5jZXNfMiR2Y292WzFdICsgdGFibGVfb2ZfdmFyaWFuY2VzXzIkdmNvdlsyXSkNCiAgdmFyaWFuY2Vfb2ZfaW5kaXZpZHVhbF9kYXRhcG9pbnRzXzIgPC0gKHRhYmxlX29mX3ZhcmlhbmNlc18yJHZjb3ZbMV0gKyB0YWJsZV9vZl92YXJpYW5jZXNfMiR2Y292WzJdICsgdGFibGVfb2ZfdmFyaWFuY2VzXzIkdmNvdlszXSkNCiAgaWNjXzIgPC0gdmFyaWFuY2Vfb2ZfbWVhbnNfMiAvIHZhcmlhbmNlX29mX2luZGl2aWR1YWxfZGF0YXBvaW50c18yDQogIA0KICAjIFNURVAgM2MuIENhbGN1bGF0ZSBnb29kbmVzcyBvZiBmaXQgYW5kIHNlZSBpZiBoaWdoZXIgZm9yIGhpZXJhcmNoaWNhbCBtb2RlbA0KICBpbXByb3ZlbWVudF9pbl9nb29kbmVzc19vZl9maXRfMl92c18xIDwtIGdvb2RuZXNzX29mX2ZpdF9oaWVyYXJjaGljYWxfbW9kZWwgLSBnb29kbmVzc19vZl9maXRfaGllcmFyY2hpY2FsX21vZGVsXzINCiAgYmV0dGVyZml0XzJfdnNfMSA8LSAxLXBjaGlzcShpbXByb3ZlbWVudF9pbl9nb29kbmVzc19vZl9maXRfMl92c18xWzFdLGRmPTEpDQogIHBfYmV0dGVyZml0XzIgPC0gaWZlbHNlKGJldHRlcmZpdF8yX3ZzXzE8MC4wMDAxLCI8MC4wMDAxIixyb3VuZChiZXR0ZXJmaXRfMl92c18xLGRpZ2l0cz00KSkNCiAgc3VwZXJpb3JwXzJfdnNfMSA8LSBpZmVsc2UoaW1wcm92ZW1lbnRfaW5fZ29vZG5lc3Nfb2ZfZml0PjAgJiBiZXR0ZXJmaXRfMl92c18xIDwgMC4wNSxwYXN0ZSgiWSAoIiwgcF9iZXR0ZXJmaXRfMiAsIikiLHNlcD0iIikscGFzdGUoIk4gKCIsIHBfYmV0dGVyZml0XzIsICIpIixzZXA9IiIpKQ0KICANCiAgIyBTVEVQIDRhIENhbGN1bGF0ZSBsZWFzdCBzcXVhcmVzIG1lYW5zIGZvciBlYWNoIGdyb3VwIG9mIHRoZSBvdXRjb21lIHZhcmlhYmxlcywgYW5kIHRoZSBwYWlyd2lzZSBjb21wYXJpc29ucyAtIGF0IGdyb3VwIGxldmVsIG9ubHkNCiAgbHNtZWFuIDwtIGxzbWVhbnNMVChoaWVyYXJjaGljYWxfbW9kZWwpICNXZSBjYWxjdWxhdGUgdGhlIGxlYXN0IHNxdWFyZXMgbWVhbnMsIHN0YW5kYXJkIGVycm9ycyBhbmQgY29uZmlkZW5jZSBpbnRlcnZhbHMgZm9yIHRoZSBkaWZmZXJlbnQgb3V0Y29tZXMgKGUuZy4gcHJlc2VuY2UgYW5kIGFic2Vuc2Ugb2YgaGVhcnQgZmFpbHVyZSkNCiAgcm93Lm5hbWVzKGxzbWVhbikgPC0gcGFzdGUoZGVwZW5kZW50X3ZhcmlhYmxlLHJvdy5uYW1lcyhsc21lYW4pLHNlcD0iIC0gIikgI0FkZCB0aGUgY3VycmVudCB2YXJpYWJsZSBpbiBxdWVzdGlvbiB0byB0aGUgcm93IG5hbWUNCiAgZGlmZmxzbWVhbiA8LSBkaWZmbHNtZWFucyhoaWVyYXJjaGljYWxfbW9kZWwpICNXZSB0ZXN0IGZvciBzaWduaWZpY2FuY2UgYmV0d2VlbiB0aGUgZGlmZmVyZW50IG91dGNvbWUgbGV2ZWxzDQogIHJvdy5uYW1lcyhkaWZmbHNtZWFuKSA8LSBwYXN0ZShkZXBlbmRlbnRfdmFyaWFibGUscm93Lm5hbWVzKGRpZmZsc21lYW4pLHNlcD0iIC0gIikgI0FkZCB0aGUgY3VycmVudCB2YXJpYWJsZSBpbiBxdWVzdGlvbiB0byB0aGUgcm93IG5hbWUNCiAgDQogICMgU1RFUCA0YiBEbyB0aGUgc2FtZSBhZ2FpbiwgYnV0IHdpdGggZ3JvdXAgYW5kIHBhcmVudGdyb3VwLWxldmVsIGFuYWx5c2lzDQogIGxzbWVhbl8yIDwtIGxzbWVhbnNMVChoaWVyYXJjaGljYWxfbW9kZWxfMikgI1dlIGNhbGN1bGF0ZSB0aGUgbGVhc3Qgc3F1YXJlcyBtZWFucywgc3RhbmRhcmQgZXJyb3JzIGFuZCBjb25maWRlbmNlIGludGVydmFscyBmb3IgdGhlIGRpZmZlcmVudCBvdXRjb21lcyAoZS5nLiBwcmVzZW5jZSBhbmQgYWJzZW5zZSBvZiBoZWFydCBmYWlsdXJlKQ0KICByb3cubmFtZXMobHNtZWFuXzIpIDwtIHBhc3RlKGRlcGVuZGVudF92YXJpYWJsZSxyb3cubmFtZXMobHNtZWFuXzIpLHNlcD0iIC0gIikgI0FkZCB0aGUgY3VycmVudCB2YXJpYWJsZSBpbiBxdWVzdGlvbiB0byB0aGUgcm93IG5hbWUNCiAgZGlmZmxzbWVhbl8yIDwtIGRpZmZsc21lYW5zKGhpZXJhcmNoaWNhbF9tb2RlbF8yKSAjV2UgdGVzdCBmb3Igc2lnbmlmaWNhbmNlIGJldHdlZW4gdGhlIGRpZmZlcmVudCBvdXRjb21lIGxldmVscw0KICByb3cubmFtZXMoZGlmZmxzbWVhbl8yKSA8LSBwYXN0ZShkZXBlbmRlbnRfdmFyaWFibGUscm93Lm5hbWVzKGRpZmZsc21lYW5fMiksc2VwPSIgLSAiKSAjQWRkIHRoZSBjdXJyZW50IHZhcmlhYmxlIGluIHF1ZXN0aW9uIHRvIHRoZSByb3cgbmFtZQ0KICANCiAgZGZfb3V0cHV0IDwtIHJiaW5kKCAjV2UgYWRkIHRoZSByZXN1bHRzIHRvIG91ciByZXN1bHRzIHRhYmxlIGluIHRoaXMgZnVuY3Rpb24NCiAgICBkZl9vdXRwdXQsDQogICAgZGF0YS5mcmFtZSgNCiAgICAgIENvbW1vblNFPXJvdW5kKHNlX05PTl9oaWVyYXJjaGljYWxfbW9kZWwsZGlnaXRzPTMpLCAjQWRkIHRoZSBzdGFuZGFyZCBlcnJvciBvZiB0aGUgbm9uLWhpZXJhcmNoaWNhbCBtb2RlbA0KICAgICAgQ29tbW9ucD1pZmVsc2UocF9OT05faGllcmFyY2hpY2FsX21vZGVsPDAuMDAwMSwiPCAwLjAwMDEiLHRvU3RyaW5nKHJvdW5kKHBfTk9OX2hpZXJhcmNoaWNhbF9tb2RlbCxkaWdpdHM9NCkpKSwgI0FkZCB0aGUgcCB2YWx1ZSBvZiB0aGUgbm9uLWhpZXJhcmNoaWNhbCBtb2RlbDsgaWYgaXQncyB2ZXJ5IHNtYWxsLCByZXBsYWNlIGl0IHdpdGggIjwgMC4wMDAxIiINCiAgICAgIElDQ19jZWxsPXBhc3RlKHJvdW5kKGljYyoxMDAsZGlnaXRzPTEpLCIlIixzZXA9IiIpLCAjQWRkIHRoZSBpbnRyYWNsYXNzIGNvcnJlbGF0aW9uIG9mIHRoZSBmaXJzdCBoaWVyYXJjaGljYWwgbW9kZWwgKGNlbGwtbGV2ZWwgZ3JvdXBpbmcpDQogICAgICBNaXhlZFNFX2NlbGw9cm91bmQoc2VfaGllcmFyY2hpY2FsX21vZGVsLGRpZ2l0cz0zKSwgI0FkZCB0aGUgc3RhbmRhcmQgZXJyb3Igb2YgdGhlIGZpcnN0IGhpZXJhcmNoaWNhbCBtb2RlbCAoY2VsbC1sZXZlbCBncm91cGluZykNCiAgICAgIE1peGVkcF9jZWxsPWlmZWxzZShwX2hpZXJhcmNoaWNhbF9tb2RlbDwwLjAwMDEsIjwgMC4wMDAxIix0b1N0cmluZyhyb3VuZChwX2hpZXJhcmNoaWNhbF9tb2RlbCxkaWdpdHM9NCkpKSwgI0FkZCB0aGUgcCB2YWx1ZSBvZiB0aGUgaGllcmFyY2hpY2FsIG1vZGVsOyBpZiBpdCdzIHZlcnkgc21hbGwsIHJlcGxhY2UgaXQgd2l0aCAiPCAwLjAwMDEiIg0KICAgICAgU3VwZXJpb3J5bj1zdXBlcmlvcnAsICNBZGQgYSBjb2x1bW4gaW5kaWNhdGluZyBpZiB0aGUgZmlyc3QgaGllcmFyY2hpY2FsIG1vZGVsIChjZWxsLWxldmVsIGdyb3VwaW5nKSBpcyBhIHN0YXRpc3RpY2FsbHkgc2lnbmlmaWNhbnRseSBiZXR0ZXIgZml0IChZIG9yIE4pIGFuZCB0aGUgYWN0dWFsIHAgdmFsdWUNCiAgICAgIElDQ19yYXRjZWxsPXBhc3RlKHJvdW5kKGljY18yKjEwMCxkaWdpdHM9MSksIiUiLHNlcD0iIiksICNBZGQgdGhlIGludHJhY2xhc3MgY29ycmVsYXRpb24gb2YgdGhlIHNlY29uZCBoaWVyYXJjaGljYWwgbW9kZWwgKHJhdCBhbmQgY2VsbC1sZXZlbCBncm91cGluZykNCiAgICAgIE1peGVkU0VfcmF0Y2VsbD1yb3VuZChzZV9oaWVyYXJjaGljYWxfbW9kZWxfMixkaWdpdHM9MyksICNBZGQgdGhlIHN0YW5kYXJkIGVycm9yIG9mIHRoZSBzZWNvbmQgaGllcmFyY2hpY2FsIG1vZGVsIChyYXQgYW5kIGNlbGwtbGV2ZWwgZ3JvdXBpbmcpDQogICAgICBNaXhlZHBfcmF0Y2VsbD1pZmVsc2UocF9oaWVyYXJjaGljYWxfbW9kZWxfMjwwLjAwMDEsIjwgMC4wMDAxIix0b1N0cmluZyhyb3VuZChwX2hpZXJhcmNoaWNhbF9tb2RlbF8yLGRpZ2l0cz00KSkpLCAjQWRkIHRoZSBwIHZhbHVlIG9mIHRoZSBoaWVyYXJjaGljYWwgbW9kZWw7IGlmIGl0J3MgdmVyeSBzbWFsbCwgcmVwbGFjZSBpdCB3aXRoICI8IDAuMDAwMSIiDQogICAgICBTdXBlcmlvcnluXzI9c3VwZXJpb3JwXzJfdnNfMSAjQWRkIGEgY29sdW1uIGluZGljYXRpbmcgaWYgdGhlIHNlY29uZCBoaWVyYXJjaGljYWwgbW9kZWwgKHJhdCBhbmQgY2VsbC1sZXZlbCBncm91cGluZykgaXMgYSBzdGF0aXN0aWNhbGx5IHNpZ25pZmljYW50bHkgYmV0dGVyIGZpdCAoWSBvciBOKSBhbmQgdGhlIGFjdHVhbCBwIHZhbHVlDQogICAgKQ0KICApDQogIA0KICBkZl9vdXRwdXRfbHNtZWFucyA8LSByYmluZCggI1dlIGFkZCB0aGUgcmVzdWx0cyB0byBvdXIgbHMgc3F1YXJlcyByZXN1bHRzIHRhYmxlIGluIHRoaXMgZnVuY3Rpb24NCiAgICBkZl9vdXRwdXRfbHNtZWFucywNCiAgICBsc21lYW4NCiAgKQ0KICANCiAgZGZfb3V0cHV0X3BhaXJ3aXNlIDwtIHJiaW5kKCAjV2UgYWRkIHRoZSByZXN1bHRzIHRvIG91ciBwYWlyd2lzZSBjb21wYXJpc29ucyByZXN1bHRzIHRhYmxlIGluIHRoaXMgZnVuY3Rpb24NCiAgICBkZl9vdXRwdXRfcGFpcndpc2UsDQogICAgZGlmZmxzbWVhbg0KICApDQogIA0KICBkZl9vdXRwdXRfbHNtZWFuc18yIDwtIHJiaW5kKCAjV2UgYWRkIHRoZSByZXN1bHRzIHRvIG91ciBscyBzcXVhcmVzIHJlc3VsdHMgdGFibGUgaW4gdGhpcyBmdW5jdGlvbg0KICAgIGRmX291dHB1dF9sc21lYW5zXzIsDQogICAgbHNtZWFuXzINCiAgKQ0KICANCiAgZGZfb3V0cHV0X3BhaXJ3aXNlXzIgPC0gcmJpbmQoICNXZSBhZGQgdGhlIHJlc3VsdHMgdG8gb3VyIHBhaXJ3aXNlIGNvbXBhcmlzb25zIHJlc3VsdHMgdGFibGUgaW4gdGhpcyBmdW5jdGlvbg0KICAgIGRmX291dHB1dF9wYWlyd2lzZV8yLA0KICAgIGRpZmZsc21lYW5fMg0KICApDQogIA0KICByb3cubmFtZXMoZGZfb3V0cHV0KVtucm93KGRmX291dHB1dCldIDwtIGRlcGVuZGVudF92YXJpYWJsZSAjU2V0IHRoZSBuYW1lIG9mIHRoZSByb3cgdG8gdGhlIGRlcGVuZGVudCB2YXJpYWJsZSBpbiBxdWVzdGlvbnMNCn0NCmBgYA0KDQpGaW5hbGx5LCB3ZSBzcGVjaWZ5IHRoZSBjb2x1bW4gaGVhZGluZ3MgaGVyZSBhbmQgcHJpbnQgdGhlIHRhYmxlLg0KWW91IG1heSBuZWVkIHRvIHNjcm9sbCByaWdodCBpbiB0aGUgdGFibGUgKHVzaW5nIHRoZSBhcnJvdyBpbiB0aGUgdG9wIHJpZ2h0IG9mIHRoZSB0YWJsZSkgdG8gdmlzdWFsaXNlIGFsbCAxMCBjb2x1bW5zDQoNCmBgYHtyfQ0KbmFtZXMoZGZfb3V0cHV0KSA9IGMoIkNvbW1vbiB0ZXN0IChTRSkiLCIocCkiLCJHcm91cC1sZXZlbCBjbHVzdGVyaW5nIChJQ0MpIiwiR3JvdXAtbGV2ZWwgKFNFKSIsIihwKSIsIlN1cGVyaW9yIGZpdCAocCkiLCJQYXJlbnRncm91cC1Hcm91cCBjbHVzdGVyaW5nIChJQ0MpIiwiUGFyZW50Z3JvdW5kLWdyb3VwIChTRSkiLCIocCkiLCJTdXBlcmlvciBmaXQgdnMuIGdyb3UtbGV2ZWwgY2x1c3RlcmluZyAocCkiKSAjU2V0IHRoZSBjb2x1bW4gaGVhZGluZ3MNCnByaW50KGRmX291dHB1dCkgI091dHB1dCB0aGUgc3VtbWFyeSB0YWJsZQ0KYGBgDQoNCkhlcmUgd2Ugb3V0cHV0IGEgdGFibGUgY29udGFpbmluZyB0aGUgbGVhc3Qgc3F1YXJlcyBtZWFucyBhbmQgY29uZmlkZW5jZSBpbnRlcnZhbHMgZm9yIGVhY2ggb3V0Y29tZSwgZ3JvdXBlZCBieSBlYWNoIGRlcGVuZGVudCB2YXJpYWJsZSwgZm9yIHRoZSBncm91cC1sZXZlbCBhbmFseXNpcy4NCllvdSBtYXkgbmVlZCB0byBzY3JvbGwgcmlnaHQgaW4gdGhlIHRhYmxlICh1c2luZyB0aGUgYXJyb3cgaW4gdGhlIHRvcCByaWdodCBvZiB0aGUgdGFibGUpIHRvIHZpc3VhbGlzZSBhbGwgOCBjb2x1bW5zLg0KDQpgYGB7cn0NCmNvbHMuZG9udC53YW50IDwtIGMoIkRGIiwgInQtdmFsdWUiLCAicC12YWx1ZSIpDQpkZl9vdXRwdXRfbHNtZWFucyA8LSBkZl9vdXRwdXRfbHNtZWFuc1ssICEgbmFtZXMoZGZfb3V0cHV0X2xzbWVhbnMpICVpbiUgY29scy5kb250LndhbnQsIGRyb3AgPSBGXQ0KcHJpbnQoZGZfb3V0cHV0X2xzbWVhbnMpICNPdXRwdXQgdGhlIHJlc3VsdHMgdGFibGUgZm9yIExTIG1lYW5zIChncm91cCBsZXZlbCBhbmFseXNpcyBvbmx5KQ0KYGBgDQoNCkhlcmUgd2Ugb3V0cHV0IHRoZSBwYWlyd2lzZSBjb21wYXJpc29ucyBmb3IgdGhlIGdyb3VwLWxldmVsIGFuYWx5c2lzDQpUaGUgcmVzdWx0cyBhcmUgZ3JvdXBlZCBmb3IgZWFjaCBvdXRwdXQgdmFyaWFibGUgKGxlZnQgY29sdW1uKSwgd2l0aCBlYWNoIGNvbWJpbmF0aW9uIG9mIG91dGNvbWVzIHVuZGVyZ29pbmcgc2lnbmlmaWNhbmNlIHRlc3RpbmcuDQpZb3UgbWF5IG5lZWQgdG8gc2Nyb2xsIHJpZ2h0IGluIHRoZSB0YWJsZSAodXNpbmcgdGhlIGFycm93IGluIHRoZSB0b3AgcmlnaHQgb2YgdGhlIHRhYmxlKSB0byB2aXN1YWxpc2UgYWxsIDcgY29sdW1ucy4NCg0KYGBge3J9DQpjb2xzLmRvbnQud2FudCA8LSBjKCJERiIsICJ0LXZhbHVlIiwgIkxvd2VyIENJIiwiVXBwZXIgQ0kiKQ0KZGZfb3V0cHV0X3BhaXJ3aXNlIDwtIGRmX291dHB1dF9wYWlyd2lzZVssICEgbmFtZXMoZGZfb3V0cHV0X3BhaXJ3aXNlKSAlaW4lIGNvbHMuZG9udC53YW50LCBkcm9wID0gRl0gI1JlbW92ZSB0aGUgdW53YW50ZWQgaGVhZGluZ3MgZnJvbSB0aGUgdGFibGUNCmRmX291dHB1dF9wYWlyd2lzZSRgcC12YWx1ZWAgPC0gZGZfb3V0cHV0X3BhaXJ3aXNlJGBQcig+fHR8KWAgKiAoIG5yb3coZGZfb3V0cHV0X3BhaXJ3aXNlKSAvIGxlbmd0aChuYW1lcyhpbnB1dF9kYXRhWyw0Om5jb2woaW5wdXRfZGF0YSldKSkgKSAjTXVsdGlwbHkgdGhlIHAgdmFsdWVzIGJ5IHRoZSBudW1iZXIgb2YgY29tcGFyaXNvbnMgcGVyIGRlcGVuZGVudCB2YXJpYWJsZQ0KbmFtZXMoZGZfb3V0cHV0X3BhaXJ3aXNlKVtuYW1lcyhkZl9vdXRwdXRfcGFpcndpc2UpPT0icC12YWx1ZSJdIDwtICJCb25mZXJyb25pIHAtdmFsdWUiICNDaGFuZ2UgdGhlIGNvbHVtbiBoZWFkaW5nDQpkZl9vdXRwdXRfcGFpcndpc2VbZGZfb3V0cHV0X3BhaXJ3aXNlJGBCb25mZXJyb25pIHAtdmFsdWVgID4gMSwiQm9uZmVycm9uaSBwLXZhbHVlIl0gPC0gMSAjQ2hhbmdlIGFueSBwIHZhbHVlIGFib3ZlIDEgdG8gMQ0KcHJpbnQoZGZfb3V0cHV0X3BhaXJ3aXNlKSAjT3V0cHV0IHRoZSByZXN1bHRzIHRhYmxlIGZvciB0aGUgcGFpcndpc2UgY29tcGFyaXNvbnMNCmBgYA0KDQpIZXJlIHdlIG91dHB1dCBhIHRhYmxlIGNvbnRhaW5pbmcgdGhlIGxlYXN0IHNxdWFyZXMgbWVhbnMgYW5kIGNvbmZpZGVuY2UgaW50ZXJ2YWxzIGZvciBlYWNoIG91dGNvbWUsIGdyb3VwZWQgYnkgZWFjaCBkZXBlbmRlbnQgdmFyaWFibGUsIGZvciB0aGUgcGFyZW50Z3JvdXAtZ3JvdXAtbGV2ZWwgYW5hbHlzaXMuDQpZb3UgbWF5IG5lZWQgdG8gc2Nyb2xsIHJpZ2h0IGluIHRoZSB0YWJsZSAodXNpbmcgdGhlIGFycm93IGluIHRoZSB0b3AgcmlnaHQgb2YgdGhlIHRhYmxlKSB0byB2aXN1YWxpc2UgYWxsIDggY29sdW1ucy4NCg0KYGBge3J9DQpjb2xzLmRvbnQud2FudCA8LSBjKCJERiIsICJ0LXZhbHVlIiwgInAtdmFsdWUiKQ0KZGZfb3V0cHV0X2xzbWVhbnNfMiA8LSBkZl9vdXRwdXRfbHNtZWFuc18yWywgISBuYW1lcyhkZl9vdXRwdXRfbHNtZWFuc18yKSAlaW4lIGNvbHMuZG9udC53YW50LCBkcm9wID0gRl0NCnByaW50KGRmX291dHB1dF9sc21lYW5zXzIpICNPdXRwdXQgdGhlIHJlc3VsdHMgdGFibGUgZm9yIExTIG1lYW5zIChncm91cCBsZXZlbCBhbmFseXNpcyBvbmx5KQ0KYGBgDQoNCkhlcmUgd2Ugb3V0cHV0IHRoZSBwYWlyd2lzZSBjb21wYXJpc29ucyBmb3IgdGhlIHBhcmVudGdyb3VwLWdyb3VwLWxldmVsIGFuYWx5c2lzDQpUaGUgcmVzdWx0cyBhcmUgZ3JvdXBlZCBmb3IgZWFjaCBvdXRwdXQgdmFyaWFibGUgKGxlZnQgY29sdW1uKSwgd2l0aCBlYWNoIGNvbWJpbmF0aW9uIG9mIG91dGNvbWVzIHVuZGVyZ29pbmcgc2lnbmlmaWNhbmNlIHRlc3RpbmcuDQpZb3UgbWF5IG5lZWQgdG8gc2Nyb2xsIHJpZ2h0IGluIHRoZSB0YWJsZSAodXNpbmcgdGhlIGFycm93IGluIHRoZSB0b3AgcmlnaHQgb2YgdGhlIHRhYmxlKSB0byB2aXN1YWxpc2UgYWxsIDcgY29sdW1ucy4NCg0KYGBge3J9DQpjb2xzLmRvbnQud2FudCA8LSBjKCJERiIsICJ0LXZhbHVlIiwgIkxvd2VyIENJIiwiVXBwZXIgQ0kiKQ0KZGZfb3V0cHV0X3BhaXJ3aXNlXzIgPC0gZGZfb3V0cHV0X3BhaXJ3aXNlXzJbLCAhIG5hbWVzKGRmX291dHB1dF9wYWlyd2lzZV8yKSAlaW4lIGNvbHMuZG9udC53YW50LCBkcm9wID0gRl0gI1JlbW92ZSB0aGUgdW53YW50ZWQgaGVhZGluZ3MgZnJvbSB0aGUgdGFibGUNCmRmX291dHB1dF9wYWlyd2lzZV8yJGBwLXZhbHVlYCA8LSBkZl9vdXRwdXRfcGFpcndpc2VfMiRgUHIoPnx0fClgICogKCBucm93KGRmX291dHB1dF9wYWlyd2lzZV8yKSAvIGxlbmd0aChuYW1lcyhpbnB1dF9kYXRhWyw0Om5jb2woaW5wdXRfZGF0YSldKSkgKSAjTXVsdGlwbHkgdGhlIHAgdmFsdWVzIGJ5IHRoZSBudW1iZXIgb2YgY29tcGFyaXNvbnMgcGVyIGRlcGVuZGVudCB2YXJpYWJsZQ0KbmFtZXMoZGZfb3V0cHV0X3BhaXJ3aXNlXzIpW25hbWVzKGRmX291dHB1dF9wYWlyd2lzZV8yKT09InAtdmFsdWUiXSA8LSAiQm9uZmVycm9uaSBwLXZhbHVlIiAjQ2hhbmdlIHRoZSBjb2x1bW4gaGVhZGluZw0KZGZfb3V0cHV0X3BhaXJ3aXNlXzJbZGZfb3V0cHV0X3BhaXJ3aXNlXzIkYEJvbmZlcnJvbmkgcC12YWx1ZWAgPiAxLCJCb25mZXJyb25pIHAtdmFsdWUiXSA8LSAxICNDaGFuZ2UgYW55IHAgdmFsdWUgYWJvdmUgMSB0byAxDQpwcmludChkZl9vdXRwdXRfcGFpcndpc2VfMikgI091dHB1dCB0aGUgcmVzdWx0cyB0YWJsZSBmb3IgdGhlIHBhaXJ3aXNlIGNvbXBhcmlzb25zICAoZ3JvdXAgbGV2ZWwgYW5hbHlzaXMgb25seSkNCmBgYA==
