# Supplementary material for: Hierarchical statistical techniques are necessary to draw reliable conclusions from analysis of isolated cardiomyocyte studies
Source: Cardiovasc Res. 2017 Aug 30;113(14):1743–52. doi: 10.1093/cvr/cvx151 (PMC5852514; doi:10.1093/cvr/cvx151)
Supplement: Supplementary Data [file cvx151_suppl_data.zip › ratandcell.pdf]

R Notebook - Rat and Cell-level hierarchical analysis

This is an R Markdown Notebook,

The source code has been separated into 'chunks' which can be run step-wise

---

How to use this script (Note green text indicates a comment rather than code)

1. Ensure the lmerTest package is installed; if unsure, type install.packages('lmerTest'); if using excel input files, you will also need to run install.packages('readxl')
2. Set your working directory in your R program to the location of your input file (which does not necessarily need to be the location of this script) - In R studio this is done by going into 'Session -> Set Working Directory'
3. Either ensure your input file is named "Hierarchical Transient analysis with Rat-Level Clustering.xlsx", or replace the code on line 50 with the appropriate filename
4. Run this file using 'Run -> Run all'

If readers would like other pragmatic examples in using mixed effect models for Hierarchical statistics, we would recommend https://www.jaredknowles.com/journal/2013/11/25/getting-started-with-mixed-effect-models-in-r

This is a PATCHED version of the original source code as of 07/08/2018 which works on the latest version of R and lmerTest.

Please contact james@jph.am for any questions

THE PROGRAM STARTS HERE

Here we load the packages we require for the analysis:

```
library(lmerTest) #If told 'there is no package called 'lmerTest', run 'install.packages("lmerTest")'
```

```
## Warning: package 'lmerTest' was built under R version 3.4.4

## Loading required package: lme4

## Warning: package 'lme4' was built under R version 3.4.4

## Loading required package: Matrix

##
## Attaching package: 'lmerTest'

## The following object is masked from 'package:lme4':
##
##     lmer

## The following object is masked from 'package:stats':
##
##     step
```

```
require(readxl) #If told 'there is no package called 'readxl', run 'install.packages("readxl")'
```

```
## Loading required package: readxl
```

This prevents scientific notation for p values unless they are very small.

```
options(scipen=999)
```

Now we load the excel spreadsheet into the variable 'input data'.

```
input_data <- read_excel("Hierarchical Spark analysis with Cell and Rat-Level Clustering.xlsx")
```

Here we rename the column of our condition (e.g. heart failure or not) to 'Condition', and the groups (e.g. cell and rat) to 'Group' and 'ParentGroup'.

We then ensure that these are treated as categorical variables (factors) rather than continuous numerical values.

Finally, we create an empty results table.

```
names(input_data)[1] <- "Condition"
names(input_data)[2] <- "Group"
names(input_data)[3] <- "ParentGroup"

input_data[1] <- as.factor(unlist(input_data[1])) #Ensure the Condition group is treated as a categoric
input_data$ParentGroup <- factor(input_data$ParentGroup) #Ensure 'Parentgroup' (Rat) is treated as a fa
input_data$Group <- factor(input_data$Group) #Ensure 'Group' (Cell) is treated as a factor

df_output <- data.frame() #Create an empty results table
df_output_lsmeans <- data.frame() #Create an empty results table for least squared means (grouo-level a
df_output_pairwise <- data.frame() #Create an empty results table for the pairway comparisons
df_output_lsmeans_2 <- data.frame() #Create an empty results table for least squared means (group- and p
df_output_pairwise_2 <- data.frame() #Create an empty results table for the pairway comparisons
```

This is the main 'loop' of the code, which runs once for each dependent variable in our spreadsheet.

```
for(dependent_variable in names(input_data[,4:ncol(input_data)])) { #This for loop ensures the indented
  # STEP 1. Fit a NON-Hierarchical model (equivalent to a t-test)
  #Fit a model, calculate the standard error, p value, and -2 log Likelihood
  NON_hierarchical_model <- glm(get(dependent_variable) ~ Condition, data=input_data) #This fits a gene
  se_NON_hierarchical_model <- summary(NON_hierarchical_model)$coefficients[, 2][2] #This extracts the s
  p_NON_hierarchical_model <- summary(NON_hierarchical_model)$coefficients[, 4][2] #This extracts the s
  p_NON_hierarchical_model <- ifelse(p_NON_hierarchical_model<0.0001,"< 0.0001",round(p_NON_hierarchical
  goodness_of_fit_NON_hierarchical_model <- logLik(NON_hierarchical_model)*-2 #We calculate a goodness

  # STEP 2. Fit a Hierachical model at the CELL level
  # Step 2a. Fit a model, calculate the standard error, p value and -2 log Likelihood
  hierarchical_model <- lmer(get(dependent_variable) ~ Condition + (1|Group), REML=FALSE ,data=input_da
  se_hierarchical_model <- summary(hierarchical_model)$coefficients[, 2][2] #As previously, extract the
  p_hierarchical_model <- summary(hierarchical_model)$coefficients[ ,5][2] #As previously, extract the p
  p_hierarchical_model <- ifelse(p_hierarchical_model<0.0001,"< 0.0001",round(p_hierarchical_model,digi
  goodness_of_fit_hierarchical_model <- logLik(hierarchical_model)*-2 #As previously, calculate a goodn

  # STEP 2b. Calculate amount of clustering, defined as the intraclass correlation, a value between 0 a
  table_of_variances <- as.data.frame(VarCorr(hierarchical_model)) #Create a dataframe (table) of the c
  variance_of_means <- table_of_variances$vcov[1] #Extract the variance of the mean from the 'table_of_
  variance_of_individual_datapoints <- (table_of_variances$vcov[1] + table_of_variances$vcov[2]) #The t
  icc <- variance_of_means / variance_of_individual_datapoints #The intra-class correlation (ICC) is ca

  # STEP 2c. Calculate goodness of fit and see if higher for hierarchical model
  improvement_in_goodness_of_fit <- goodness_of_fit_NON_hierarchical_model - goodness_of_fit_hierarchica
  betterfit <- 1-pchisq(improvement_in_goodness_of_fit[1],df=1) #The p value for whether the hierarchic
```

```
p_betterfit <- ifelse(betterfit<0.0001,"<0.0001",round(betterfit,digits=4)) #If the p value calculate
superiorp <- ifelse(improvement_in_goodness_of_fit>0 & betterfit < 0.05,paste("Y (", p_betterfit ,")"


# STEP 3. Fit a Hierachical model at the CELL and RAT levels - this is merely a repeat of STEPS 2a, 2
# Step 3a. Fit a model, calculate the standard error, p value and -2 log Likelihood
hierarchical_model_2 <- lmer(get(dependent_variable) ~ Condition + (1|ParentGroup/Group), REML=FALSE
se_hierarchical_model_2 <- summary(hierarchical_model_2)$coefficients[, 2][2]
p_hierarchical_model_2 <- summary(hierarchical_model_2)$coefficients[ ,5][2]
p_hierarchical_model_2 <- ifelse(p_hierarchical_model_2<0.0001,"< 0.0001",round(p_hierarchical_model_2
goodness_of_fit_hierarchical_model_2 <- logLik(hierarchical_model_2)*-2

# STEP 3b. Calculate amount of clustering, defined as the intraclass correlation, a value between 0 a
table_of_variances_2 <- as.data.frame(VarCorr(hierarchical_model_2))
variance_of_means_2 <- (table_of_variances_2$vcov[1] + table_of_variances_2$vcov[2])
variance_of_individual_datapoints_2 <- (table_of_variances_2$vcov[1] + table_of_variances_2$vcov[2] +
icc_2 <- variance_of_means_2 / variance_of_individual_datapoints_2

# STEP 3c. Calculate goodness of fit and see if higher for hierarchical model
improvement_in_goodness_of_fit_2_vs_1 <- goodness_of_fit_hierarchical_model - goodness_of_fit_hierarch
betterfit_2_vs_1 <- 1-pchisq(improvement_in_goodness_of_fit_2_vs_1[1],df=1)
p_betterfit_2 <- ifelse(betterfit_2_vs_1<0.0001,"<0.0001",round(betterfit_2_vs_1,digits=4))
superiorp_2_vs_1 <- ifelse(improvement_in_goodness_of_fit>0 & betterfit_2_vs_1 < 0.05,paste("Y (", p_b

# STEP 4a Calculate least squares means for each group of the outcome variables, and the pairwise com
lsmean <- lsmeansLT(hierarchical_model) #We calculate the least squares means, standard errors and co
row.names(lsmean) <- paste(dependent_variable,row.names(lsmean),sep=" - ") #Add the current variable
difflsmean <- difflsmeans(hierarchical_model) #We test for significance between the different outcome
row.names(difflsmean) <- paste(dependent_variable,row.names(difflsmean),sep=" - ") #Add the current v

# STEP 4b Do the same again, but with group and parentgroup-level analysis
lsmean_2 <- lsmeansLT(hierarchical_model_2) #We calculate the least squares means, standard errors an
row.names(lsmean_2) <- paste(dependent_variable,row.names(lsmean_2),sep=" - ") #Add the current varia
difflsmean_2 <- difflsmeans(hierarchical_model_2) #We test for significance between the different out
row.names(difflsmean_2) <- paste(dependent_variable,row.names(difflsmean_2),sep=" - ") #Add the curre

df_output <- rbind( #We add the results to our results table in this function
  df_output,
  data.frame(
    CommonSE=round(se_NON_hierarchical_model,digits=3), #Add the standard error of the non-hierarchic
    Commonp=ifelse(p_NON_hierarchical_model<0.0001,"< 0.0001",toString(round(p_NON_hierarchical_model
    ICC_cell=paste(round(icc*100,digits=1),"%",sep=""), #Add the intraclass correlation of the first
    MixedSE_cell=round(se_hierarchical_model,digits=3), #Add the standard error of the first hierarch
    Mixedp_cell=ifelse(p_hierarchical_model<0.0001,"< 0.0001",toString(round(p_hierarchical_model,dig
    Superioryn=superiorp, #Add a column indicating if the first hierarchical model (cell-level groupi
    ICC_ratcell=paste(round(icc_2*100,digits=1),"%",sep=""), #Add the intraclass correlation of the s
    MixedSE_ratcell=round(se_hierarchical_model_2,digits=3), #Add the standard error of the second hi
    Mixedp_ratcell=ifelse(p_hierarchical_model_2<0.0001,"< 0.0001",toString(round(p_hierarchical_model
    Superioryn_2=superiorp_2_vs_1 #Add a column indicating if the second hierarchical model (rat and
  )
)

df_output_lsmeans <- rbind( #We add the results to our ls squares results table in this function
```

```r
    df_output_lsmeans,
    lsmean
  )

  df_output_pairwise <- rbind( #We add the results to our pairwise comparisons results table in this fu
    df_output_pairwise,
    difflsmean
  )

  df_output_lsmeans_2 <- rbind( #We add the results to our ls squares results table in this function
    df_output_lsmeans_2,
    lsmean_2
  )

  df_output_pairwise_2 <- rbind( #We add the results to our pairwise comparisons results table in this 
    df_output_pairwise_2,
    difflsmean_2
  )

  row.names(df_output)[nrow(df_output)] <- dependent_variable #Set the name of the row to the dependent
}
```

Finally, we specify the column headings here and print the table. You may need to scroll right in the table (using the arrow in the top right of the table) to visualise all 10 columns

```r
names(df_output) = c("Common test (SE)","(p)","Group-level clustering (ICC)","Group-level (SE)","(p)","S
print(df_output) #Output the summary table
```

```
##         Common test (SE)      (p) Group-level clustering (ICC)
## LogAmp             0.009 < 0.0001                        47.1%
## LogFWHM            0.011   0.3811                         7.1%
## LogFDHM            0.017   0.0231                         8.5%
##         Group-level (SE)    (p) Superior fit (p)
## LogAmp             0.032  0.001      Y (<0.0001)
## LogFWHM            0.018 0.7818      Y (<0.0001)
## LogFDHM            0.029 0.7781      Y (<0.0001)
##         Parentgroup-Group clustering (ICC) Parentground-group (SE)    (p)
## LogAmp                               57.9%                   0.064 0.2395
## LogFWHM                               7.1%                   0.018 0.7818
## LogFDHM                                 8%                   0.036 0.8692
##         Superior fit vs. grou-level clustering (p)
## LogAmp                                Y (0.0009)
## LogFWHM                                    N (1)
## LogFDHM                               N (0.3459)
```

Here we output a table containing the least squares means and confidence intervals for each outcome, grouped by each dependent variable, for the group-level analysis. You may need to scroll right in the table (using the arrow in the top right of the table) to visualise all 8 columns.

```r
cols.dont.want <- c("DF", "t-value", "p-value")
df_output_lsmeans <- df_output_lsmeans[, ! names(df_output_lsmeans) %in% cols.dont.want, drop = F]
print(df_output_lsmeans) #Output the results table for LS means (group level analysis only)
```

```
##                      Estimate Std. Error   df t value     lower     upper
## LogAmp - Condition0  -0.219245   0.023867 33.2 -9.1863 -0.267790 -0.170701
## LogAmp - Condition1  -0.104356   0.021373 34.8 -4.8827 -0.147752 -0.060960
```

```
## LogFWHM - Condition0  0.407808   0.013607 32.6 29.9696  0.380111  0.435506
## LogFWHM - Condition1  0.412964   0.012477 34.7 33.0993  0.387628  0.438300
## LogFDHM - Condition0  1.345823   0.021304 30.7 63.1724  1.302357  1.389289
## LogFDHM - Condition1  1.337614   0.019501 32.5 68.5923  1.297915  1.377312
##                                     Pr(>|t|)
## LogAmp - Condition0        0.0000000001218 ***
## LogAmp - Condition1        0.0000230850611 ***
## LogFWHM - Condition0 < 0.00000000000000022 ***
## LogFWHM - Condition1 < 0.00000000000000022 ***
## LogFDHM - Condition0 < 0.00000000000000022 ***
## LogFDHM - Condition1 < 0.00000000000000022 ***
## ---
## Signif. codes:  0 '***' 0.001 '**' 0.01 '*' 0.05 '.' 0.1 ' ' 1
```

Here we output the pairwise comparisons for the group-level analysis The results are grouped for each output variable (left column), with each combination of outcomes undergoing significance testing. You may need to scroll right in the table (using the arrow in the top right of the table) to visualise all 7 columns.

```
cols.dont.want <- c("DF", "t-value", "Lower CI","Upper CI")
df_output_pairwise <- df_output_pairwise[, ! names(df_output_pairwise) %in% cols.dont.want, drop = F] #
df_output_pairwise$`p-value` <- df_output_pairwise$`Pr(>|t|)` * ( nrow(df_output_pairwise) / length(name
names(df_output_pairwise)[names(df_output_pairwise)=="p-value"] <- "Bonferroni p-value" #Change the col
df_output_pairwise[df_output_pairwise$`Bonferroni p-value` > 1,"Bonferroni p-value"] <- 1 #Change any p
print(df_output_pairwise) #Output the results table for the pairwise comparisons
```

```
##                                    Estimate Std. Error   df t value
## LogAmp - Condition0 - Condition1  -0.1148895  0.0320375 33.9 -3.5861
## LogFWHM - Condition0 - Condition1 -0.0051554  0.0184614 33.5 -0.2792
## LogFDHM - Condition0 - Condition1  0.0082091  0.0288816 31.5  0.2842
##                                        lower      upper  Pr(>|t|)
## LogAmp - Condition0 - Condition1  -0.1800026 -0.0497764 0.0010434
## LogFWHM - Condition0 - Condition1 -0.0426925  0.0323817 0.7817678
## LogFDHM - Condition0 - Condition1 -0.0506567  0.0670749 0.7780916
##                                   Bonferroni p-value
## LogAmp - Condition0 - Condition1            0.001043 **
## LogFWHM - Condition0 - Condition1           0.781768
## LogFDHM - Condition0 - Condition1           0.778092
## ---
## Signif. codes:  0 '***' 0.001 '**' 0.01 '*' 0.05 '.' 0.1 ' ' 1
```

Here we output a table containing the least squares means and confidence intervals for each outcome, grouped by each dependent variable, for the parentgroup-group-level analysis. You may need to scroll right in the table (using the arrow in the top right of the table) to visualise all 8 columns.

```
cols.dont.want <- c("DF", "t-value", "p-value")
df_output_lsmeans_2 <- df_output_lsmeans_2[, ! names(df_output_lsmeans_2) %in% cols.dont.want, drop = F
print(df_output_lsmeans_2) #Output the results table for LS means (group level analysis only)
```

```
##                        Estimate Std. Error   df t value      lower
## LogAmp - Condition0  -0.1952993  0.0428872  9.1 -4.5538 -0.2921650
## LogAmp - Condition1  -0.1137883  0.0476393  7.1 -2.3885 -0.2259750
## LogFWHM - Condition0  0.4078084  0.0136074 32.6 29.9696  0.3801108
## LogFWHM - Condition1  0.4129638  0.0124765 34.7 33.0993  0.3876278
## LogFDHM - Condition0  1.3389152  0.0259005  8.7 51.6946  1.2799794
## LogFDHM - Condition1  1.3328168  0.0245965  5.9 54.1873  1.2723322
##                           upper             Pr(>|t|)
```

```
## LogAmp - Condition0  -0.0984336             0.001342 **
## LogAmp - Condition1  -0.0016015             0.047566 *
## LogFWHM - Condition0  0.4355060 < 0.00000000000000022 ***
## LogFWHM - Condition1  0.4382997 < 0.00000000000000022 ***
## LogFDHM - Condition0  1.3978510     0.000000000004253 ***
## LogFDHM - Condition1  1.3933015     0.000000003659326 ***
## ---
## Signif. codes:  0 '***' 0.001 '**' 0.01 '*' 0.05 '.' 0.1 ' ' 1
```

Here we output the pairwise comparisons for the parentgroup-group-level analysis The results are grouped for each output variable (left column), with each combination of outcomes undergoing significance testing. You may need to scroll right in the table (using the arrow in the top right of the table) to visualise all 7 columns.

```
cols.dont.want <- c("DF", "t-value", "Lower CI","Upper CI")
df_output_pairwise_2 <- df_output_pairwise_2[, ! names(df_output_pairwise_2) %in% cols.dont.want, drop =
df_output_pairwise_2$`p-value` <- df_output_pairwise_2$`Pr(>|t|)` * ( nrow(df_output_pairwise_2) / leng
names(df_output_pairwise_2)[names(df_output_pairwise_2)=="p-value"] <- "Bonferroni p-value" #Change the
df_output_pairwise_2[df_output_pairwise_2$`Bonferroni p-value` > 1,"Bonferroni p-value"] <- 1 #Change a
print(df_output_pairwise_2) #Output the results table for the pairwise comparisons  (group level analys
```

```
##                                     Estimate Std. Error   df t value
## LogAmp - Condition0 - Condition1  -0.0815110  0.0641000  7.9 -1.2716
## LogFWHM - Condition0 - Condition1 -0.0051554  0.0184614 33.5 -0.2792
## LogFDHM - Condition0 - Condition1  0.0060984  0.0357186  7.2  0.1707
##                                        lower      upper Pr(>|t|)
## LogAmp - Condition0 - Condition1  -0.2295304  0.0665083  0.23950
## LogFWHM - Condition0 - Condition1 -0.0426924  0.0323817  0.78177
## LogFDHM - Condition0 - Condition1 -0.0779909  0.0901876  0.86916
##                                   Bonferroni p-value
## LogAmp - Condition0 - Condition1              0.2395
## LogFWHM - Condition0 - Condition1             0.7818
## LogFDHM - Condition0 - Condition1             0.8692
```
