# Supplementary material for: Hierarchical statistical techniques are necessary to draw reliable conclusions from analysis of isolated cardiomyocyte studies
Source: Cardiovasc Res. 2017 Aug 30;113(14):1743–52. doi: 10.1093/cvr/cvx151 (PMC5852514; doi:10.1093/cvr/cvx151)
Supplement: Supplementary Data [file cvx151_suppl_data.zip › ratonly.nb.html]

R Notebook - Rat-level hierarchical analysis


Code 

- Show All Code
- Hide All Code
- Download Rmd

# R Notebook - Rat-level hierarchical analysis

This is an R Markdown Notebook,

The source code has been separated into ‘chunks’ which can be run step-wise

---

How to use this script (Note green text indicates a comment rather than code)

1. Ensure the lmerTest package is installed; if unsure, type install.packages(‘lmerTest’); if using excel input files, you will also need to run install.packages(‘readxl’)
2. Set your working directory in your R program to the location of your input file (which does not necessarily need to be the location of this script) - In R studio this is done by going into ‘Session -> Set Working Directory’
3. Either ensure your input file is named “Hierarchical Transient analysis with Rat-Level Clustering.xlsx”, or replace the code on line 50 with the appropriate filename
4. Run this file using ‘Run -> Run all’

If readers would like other pragmatic examples in using mixed effect models for Hierarchical statistics, we would recommend https://www.jaredknowles.com/journal/2013/11/25/getting-started-with-mixed-effect-models-in-r

This is a PATCHED version of the original source code as of 07/08/2018 which works on the latest version of R and lmerTest.

Please contact james@jph.am for any questions

---

THE PROGRAM STARTS HERE

---

Here we load the packages we require for the analysis:


```
library(lmerTest) #If told 'there is no package called 'lmerTest', run 'install.packages("lmerTest")'
require(readxl) #If told 'there is no package called 'readxl', run 'install.packages("readxl")'
```


This prevents scientific notation for p values unless they are very small.


```
options(scipen=999)
```


Now we load the excel spreadsheet into the variable ‘input data’.


```
input_data <- read_excel("Hierarchical Transient analysis with Rat-Level Clustering.xlsx") #This loads the excel spreadsheet into the variable 'input data'
```


Here we rename the column of our condition (e.g. heart failure or not) to ‘Condition’, and the grouping variable (e.g. rat) to ‘Group’.

We then ensure that the condition and group columns are treated as categorical variables (factors) rather than a continuous numerical value.

Finally, we create an empty results table.


```
names(input_data)[1] <- "Condition"
names(input_data)[2] <- "Group"
input_data[1] <- as.factor(unlist(input_data[1])) #Ensure the Condition group is treated as a categorical variable (distrinct groups) rather than continuous numerical values
input_data$Group <- factor(input_data$Group) #Ensure the Rat group (Group) is treated as a categorical variable (distrinct groups) rather than continuous numerical values
df_output <- data.frame() #Create an empty main results table
df_output_lsmeans <- data.frame() #Create an empty results table for least squared means
df_output_pairwise <- data.frame() #Create an empty results table for the pairway comparisons
```


This is the main ‘loop’ of the code, which runs once for each dependent variable in our spreadsheet.


```
for(dependent_variable in names(input_data[,3:ncol(input_data)])) { #This for loop ensures the indented code between the curly braces runs once for each column (dependent variable) from the 3rd column onwards
  # STEP 1. Fit a NON-Hierarchical model (equivalent to a t-test)
  #Fit a model, calculate the standard error, p value, and -2 log Likelihood
  NON_hierarchical_model <- glm(get(dependent_variable) ~ Condition, data=input_data) #This fits a generlised linear model for each dependent variable and the output column (HF_or_control; the presence or not of heart failure) and stores it in the variable named NON_hierarchical_model
  se_NON_hierarchical_model <- summary(NON_hierarchical_model)$coefficients[, 2][2] #This extracts the standard errors from the model and stores it in a new variable named se_NON_hierarchical_model
  p_NON_hierarchical_model <- summary(NON_hierarchical_model)$coefficients[, 4][2] #This extracts the standard errors from the model and stores it in a new variable named p_NON_hierarchical_model
  goodness_of_fit_NON_hierarchical_model <- logLik(NON_hierarchical_model)*-2 #We calculate a goodness of fit using the -2 logLikelihood of the model and save it in a new variable named goodness_of_fit_NON_hierarchical_model
  
  # STEP 2. Fit a Hierachical model
  # Step 2a. Fit a model, calculate the standard error, p value and -2 log Likelihood
  hierarchical_model <- lmer(get(dependent_variable) ~ Condition + (1|Group), REML=FALSE ,data=input_data) #We now fit a linear mixed effects (hierarchical) model and store that in the variable named hierarchical_model. We fit each dependent variable against the Output column, with 'Rat' as a group effect
  se_hierarchical_model <- summary(hierarchical_model)$coefficients[, 2][2] #As previously, extract the standard errors and store them in a variable
  p_hierarchical_model <- summary(hierarchical_model)$coefficients[ ,5][2] #As previously, extract the p values and store them in a variable
  goodness_of_fit_hierarchical_model <- logLik(hierarchical_model)*-2 #As previously, calculate a goodness of fit and store it in a variable
  
  # STEP 2b. Calculate amount of clustering, defined as the intraclass correlation, a value between 0 and 1 
  table_of_variances <- as.data.frame(VarCorr(hierarchical_model)) #Create a dataframe (table) of the covariance parameters called 'table_of_variances'
  variance_of_means <- table_of_variances$vcov[1] #Extract the variance of the mean from the 'table_of_variances' and store it in a variable names 'variance_of_means'
  variance_of_individual_datapoints <- (table_of_variances$vcov[1] + table_of_variances$vcov[2]) #The total variance is also extracted, as defined as the variance of the rat model plus the residual variance
  icc <- variance_of_means / variance_of_individual_datapoints #The intra-class correlation (ICC) is calculated and defined by the variance of the model divided by the total variance
  
  # STEP 2c. Calculate goodness of fit and see if higher for hierarchical model
  improvement_in_goodness_of_fit <- goodness_of_fit_NON_hierarchical_model - goodness_of_fit_hierarchical_model #We calculate the difference between thepreviously-calculated goodness of fit for both the non-hierarchical and hierrachical models; this difference is stored in the variable improvement_in_goodness_of_fit
  betterfit <- 1-pchisq(improvement_in_goodness_of_fit[1],df=1) #The p value for whether the hierarchical model is a significant improvement is calculated using the Chi-squared distribution with 1 degree of freedom
  p_betterfit <- ifelse(betterfit<0.0001,"<0.0001",round(betterfit,digits=4)) #If the p value calculated above is very small, we just replace it with "<0.0001"
  superiorp <- ifelse(improvement_in_goodness_of_fit>0 & betterfit < 0.05,paste("Y (", p_betterfit ,")",sep=""),paste("N (", betterfit, ")",sep="")) #If there is an improvement in the goodness of fit and the p value is less than <0.05, we set the variable 'superiorp' to "Y", otherwise "N"
  
  # STEP 3 Calculate least squares means for each group of the outcome variables, and the pairwise comparisons
  lsmean <- lsmeansLT(hierarchical_model) #We calculate the least squares means, standard errors and confidence intervals for the different outcomes (e.g. presence and absense of heart failure)
  row.names(lsmean) <- paste(dependent_variable,row.names(lsmean),sep=" - ") #Add the current variable in question to the row name
  difflsmean <- difflsmeans(hierarchical_model) #We test for significance between the different outcome levels
  row.names(difflsmean) <- paste(dependent_variable,row.names(difflsmean),sep=" - ") #Add the current variable in question to the row name
  
  df_output <- rbind( #We add the results to our main results table in this function
    df_output,
    data.frame(
      CommonSE=round(se_NON_hierarchical_model,digits=3), #Add the standard error of the non-hierarchical model
      Commonp=ifelse(p_NON_hierarchical_model<0.0001,"< 0.0001",toString(round(p_NON_hierarchical_model,digits=4))), #Add the p value of the non-hierarchical model; if it's very small, replace it with "< 0.0001""
      ICC=paste(round(icc*100,digits=1),"%",sep=""), #Add the intraclass correlation of the hierarchical model
      MixedSE=round(se_hierarchical_model,digits=3), #Add the standard error of the hierarchical model
      Mixedp=ifelse(p_hierarchical_model<0.0001,"< 0.0001",toString(round(p_hierarchical_model,digits=4))), #Add the p value of the hierarchical model; if it's very small, replace it with "< 0.0001""
      Superioryn=superiorp #Add a column indicating if the hierarchical model is a statistically significantly better fit (Y or N) and the actual p value
    )
  )
  df_output_lsmeans <- rbind( #We add the results to our ls squares results table in this function
    df_output_lsmeans,
    lsmean
  )
  
  df_output_pairwise <- rbind( #We add the results to our pairwise comparisons results table in this function
    df_output_pairwise,
    difflsmean
  )
  
  row.names(df_output)[nrow(df_output)] <- dependent_variable #Set the name of the row to the dependent variable in questions
}
```


Finally, we specify the column headings here and print the table.


```
names(df_output) = c("Common test (SE)","Common test (p)","Group-level clustering (ICC)","Group-level (SE)","Group-level (p)","Superior fit (p)") #Set the column headings
print(df_output) #Output the summary table
```


Here we output a table containing the least squares means and confidence intervals for each outcome, grouped by each dependent variable.


```
cols.dont.want <- c("DF", "t-value", "p-value")
df_output_lsmeans <- df_output_lsmeans[, ! names(df_output_lsmeans) %in% cols.dont.want, drop = F]
print(df_output_lsmeans) #Output the results table for LS means
```


```
                                   Estimate Std. Error   df t value      lower      upper              Pr(>|t|)    
Transient Amplitude - Condition0 1.38580799 0.03484998 14.6  39.765 1.31134537 1.46027061 0.0000000000000002749 ***
Transient Amplitude - Condition1 1.53158208 0.03480867 14.3  44.000 1.45708901 1.60607515 < 0.00000000000000022 ***
Baseline - Condition0            1.06298741 0.01436812 20.5  73.982 1.03306339 1.09291143 < 0.00000000000000022 ***
Baseline - Condition1            1.05285216 0.01436224 20.3  73.307 1.02292228 1.08278203 < 0.00000000000000022 ***
Peak - Condition0                1.47458245 0.04620646 15.8  31.913 1.37650689 1.57265801 0.0000000000000009597 ***
Peak - Condition1                1.61588544 0.04616393 15.5  35.003 1.51777773 1.71399315 0.0000000000000003415 ***
Tto50%Peak - Condition0          0.02650272 0.00050711 18.3  52.262 0.02543855 0.02756688 < 0.00000000000000022 ***
Tto50%Peak - Condition1          0.02770918 0.00050527 17.6  54.840 0.02664602 0.02877235 < 0.00000000000000022 ***
Tto50%decay - Condition0         0.09760852 0.00694241 19.6  14.060 0.08311023 0.11210681 0.0000000000103350884 ***
Tto50%decay - Condition1         0.08915714 0.00694445 19.6  12.839 0.07465316 0.10366112 0.0000000000532026194 ***
Tau - Condition0                 0.14715677 0.01248907 19.6  11.783 0.12107243 0.17324112 0.0000000002381886041 ***
Tau - Condition1                 0.13272588 0.01249307 19.6  10.624 0.10663147 0.15882029 0.0000000014110491745 ***
---
Signif. codes:  0 *** 0.001 ** 0.01 * 0.05 . 0.1   1
```


Here we output the pairwise comparisons. P values are multiplied by the number of comparisons to maintain a type 1 error rate of 5% (Bonferroni correction) The results are grouped for each output variable (left column), with each combination of outcomes undergoing significance testing.


```
cols.dont.want <- c("DF", "t-value", "Lower CI","Upper CI")
df_output_pairwise <- df_output_pairwise[, ! names(df_output_pairwise) %in% cols.dont.want, drop = F] #Remove the unwanted headings from the table
df_output_pairwise$`p-value` <- df_output_pairwise$`Pr(>|t|)` * ( nrow(df_output_pairwise) / length(names(input_data[,3:ncol(input_data)])) ) #Multiply the p values by the number of comparisons per dependent variable
names(df_output_pairwise)[names(df_output_pairwise)=="p-value"] <- "Bonferroni p-value" #Change the column heading
df_output_pairwise[df_output_pairwise$`Bonferroni p-value` > 1,"Bonferroni p-value"] <- 1 #Change any p value above 1 to 1
print(df_output_pairwise) #Output the results table for the pairwise comparisons
```


```
                                                 Estimate  Std. Error   df t value       lower       upper Pr(>|t|) Bonferroni p-value  
Transient Amplitude - Condition0 - Condition1 -0.14577409  0.04925611 14.5 -2.9595 -0.25110137 -0.04044681 0.010056            0.01006 *
Baseline - Condition0 - Condition1             0.01013526  0.02031543 20.4  0.4989 -0.03218782  0.05245833 0.623189            0.62319  
Peak - Condition0 - Condition1                -0.14130299  0.06531574 15.6 -2.1634 -0.28002526 -0.00258072 0.046360            0.04636 *
Tto50%Peak - Condition0 - Condition1          -0.00120647  0.00071586 18.0 -1.6853 -0.00271070  0.00029777 0.109225            0.10922  
Tto50%decay - Condition0 - Condition1          0.00845138  0.00981949 19.6  0.8607 -0.01205633  0.02895909 0.399807            0.39981  
Tau - Condition0 - Condition1                  0.01443089  0.01766504 19.6  0.8169 -0.02246506  0.05132684 0.423790            0.42379  
---
Signif. codes:  0 *** 0.001 ** 0.01 * 0.05 . 0.1   1
```


LS0tDQp0aXRsZTogIlIgTm90ZWJvb2sgLSBSYXQtbGV2ZWwgaGllcmFyY2hpY2FsIGFuYWx5c2lzIg0Kb3V0cHV0OiBodG1sX25vdGVib29rDQotLS0NCg0KVGhpcyBpcyBhbiBbUiBNYXJrZG93bl0oaHR0cDovL3JtYXJrZG93bi5yc3R1ZGlvLmNvbSkgTm90ZWJvb2ssIA0KDQpUaGUgc291cmNlIGNvZGUgaGFzIGJlZW4gc2VwYXJhdGVkIGludG8gJ2NodW5rcycgd2hpY2ggY2FuIGJlIHJ1biBzdGVwLXdpc2UNCg0KLS0tDQoNCkhvdyB0byB1c2UgdGhpcyBzY3JpcHQgKE5vdGUgZ3JlZW4gdGV4dCBpbmRpY2F0ZXMgYSBjb21tZW50IHJhdGhlciB0aGFuIGNvZGUpDQogDQoxLiBFbnN1cmUgdGhlIGxtZXJUZXN0IHBhY2thZ2UgaXMgaW5zdGFsbGVkOyBpZiB1bnN1cmUsIHR5cGUgaW5zdGFsbC5wYWNrYWdlcygnbG1lclRlc3QnKTsgaWYgdXNpbmcgZXhjZWwgaW5wdXQgZmlsZXMsIHlvdSB3aWxsIGFsc28gbmVlZCB0byBydW4gaW5zdGFsbC5wYWNrYWdlcygncmVhZHhsJykNCjIuIFNldCB5b3VyIHdvcmtpbmcgZGlyZWN0b3J5IGluIHlvdXIgUiBwcm9ncmFtIHRvIHRoZSBsb2NhdGlvbiBvZiB5b3VyIGlucHV0IGZpbGUgKHdoaWNoIGRvZXMgbm90IG5lY2Vzc2FyaWx5IG5lZWQgdG8gYmUgdGhlIGxvY2F0aW9uIG9mIHRoaXMgc2NyaXB0KSAtIEluIFIgc3R1ZGlvIHRoaXMgaXMgZG9uZSBieSBnb2luZyBpbnRvICdTZXNzaW9uIC0+IFNldCBXb3JraW5nIERpcmVjdG9yeScNCjMuIEVpdGhlciBlbnN1cmUgeW91ciBpbnB1dCBmaWxlIGlzIG5hbWVkICJIaWVyYXJjaGljYWwgVHJhbnNpZW50IGFuYWx5c2lzIHdpdGggUmF0LUxldmVsIENsdXN0ZXJpbmcueGxzeCIsIG9yIHJlcGxhY2UgdGhlIGNvZGUgb24gbGluZSA1MCB3aXRoIHRoZSBhcHByb3ByaWF0ZSBmaWxlbmFtZQ0KNC4gUnVuIHRoaXMgZmlsZSB1c2luZyAnUnVuIC0+IFJ1biBhbGwnDQoNCklmIHJlYWRlcnMgd291bGQgbGlrZSBvdGhlciBwcmFnbWF0aWMgZXhhbXBsZXMgaW4gdXNpbmcgbWl4ZWQgZWZmZWN0IG1vZGVscyBmb3IgSGllcmFyY2hpY2FsIHN0YXRpc3RpY3MsIHdlIHdvdWxkIHJlY29tbWVuZCBodHRwczovL3d3dy5qYXJlZGtub3dsZXMuY29tL2pvdXJuYWwvMjAxMy8xMS8yNS9nZXR0aW5nLXN0YXJ0ZWQtd2l0aC1taXhlZC1lZmZlY3QtbW9kZWxzLWluLXINCg0KVGhpcyBpcyBhIFBBVENIRUQgdmVyc2lvbiBvZiB0aGUgb3JpZ2luYWwgc291cmNlIGNvZGUgYXMgb2YgMDcvMDgvMjAxOCB3aGljaCB3b3JrcyBvbiB0aGUgbGF0ZXN0IHZlcnNpb24gb2YgUiBhbmQgbG1lclRlc3QuDQogDQpQbGVhc2UgY29udGFjdCBqYW1lc0BqcGguYW0gZm9yIGFueSBxdWVzdGlvbnMNCg0KLS0tDQoNClRIRSBQUk9HUkFNIFNUQVJUUyBIRVJFDQoNCi0tLQ0KDQpIZXJlIHdlIGxvYWQgdGhlIHBhY2thZ2VzIHdlIHJlcXVpcmUgZm9yIHRoZSBhbmFseXNpczoNCmBgYHtyfQ0KbGlicmFyeShsbWVyVGVzdCkgI0lmIHRvbGQgJ3RoZXJlIGlzIG5vIHBhY2thZ2UgY2FsbGVkICdsbWVyVGVzdCcsIHJ1biAnaW5zdGFsbC5wYWNrYWdlcygibG1lclRlc3QiKScNCnJlcXVpcmUocmVhZHhsKSAjSWYgdG9sZCAndGhlcmUgaXMgbm8gcGFja2FnZSBjYWxsZWQgJ3JlYWR4bCcsIHJ1biAnaW5zdGFsbC5wYWNrYWdlcygicmVhZHhsIiknDQpgYGANCg0KVGhpcyBwcmV2ZW50cyBzY2llbnRpZmljIG5vdGF0aW9uIGZvciBwIHZhbHVlcyB1bmxlc3MgdGhleSBhcmUgdmVyeSBzbWFsbC4NCg0KYGBge3J9DQpvcHRpb25zKHNjaXBlbj05OTkpDQpgYGANCg0KTm93IHdlIGxvYWQgdGhlIGV4Y2VsIHNwcmVhZHNoZWV0IGludG8gdGhlIHZhcmlhYmxlICdpbnB1dCBkYXRhJy4NCg0KYGBge3J9DQppbnB1dF9kYXRhIDwtIHJlYWRfZXhjZWwoIkhpZXJhcmNoaWNhbCBUcmFuc2llbnQgYW5hbHlzaXMgd2l0aCBSYXQtTGV2ZWwgQ2x1c3RlcmluZy54bHN4IikgI1RoaXMgbG9hZHMgdGhlIGV4Y2VsIHNwcmVhZHNoZWV0IGludG8gdGhlIHZhcmlhYmxlICdpbnB1dCBkYXRhJw0KYGBgDQoNCkhlcmUgd2UgcmVuYW1lIHRoZSBjb2x1bW4gb2Ygb3VyIGNvbmRpdGlvbiAoZS5nLiBoZWFydCBmYWlsdXJlIG9yIG5vdCkgdG8gJ0NvbmRpdGlvbicsIGFuZCB0aGUgZ3JvdXBpbmcgdmFyaWFibGUgKGUuZy4gcmF0KSB0byAnR3JvdXAnLg0KDQpXZSB0aGVuIGVuc3VyZSB0aGF0IHRoZSBjb25kaXRpb24gYW5kIGdyb3VwIGNvbHVtbnMgYXJlIHRyZWF0ZWQgYXMgY2F0ZWdvcmljYWwgdmFyaWFibGVzIChmYWN0b3JzKSByYXRoZXIgdGhhbiBhIGNvbnRpbnVvdXMgbnVtZXJpY2FsIHZhbHVlLg0KDQpGaW5hbGx5LCB3ZSBjcmVhdGUgYW4gZW1wdHkgcmVzdWx0cyB0YWJsZS4NCg0KYGBge3J9DQpuYW1lcyhpbnB1dF9kYXRhKVsxXSA8LSAiQ29uZGl0aW9uIg0KbmFtZXMoaW5wdXRfZGF0YSlbMl0gPC0gIkdyb3VwIg0KDQppbnB1dF9kYXRhWzFdIDwtIGFzLmZhY3Rvcih1bmxpc3QoaW5wdXRfZGF0YVsxXSkpICNFbnN1cmUgdGhlIENvbmRpdGlvbiBncm91cCBpcyB0cmVhdGVkIGFzIGEgY2F0ZWdvcmljYWwgdmFyaWFibGUgKGRpc3RyaW5jdCBncm91cHMpIHJhdGhlciB0aGFuIGNvbnRpbnVvdXMgbnVtZXJpY2FsIHZhbHVlcw0KaW5wdXRfZGF0YSRHcm91cCA8LSBmYWN0b3IoaW5wdXRfZGF0YSRHcm91cCkgI0Vuc3VyZSB0aGUgUmF0IGdyb3VwIChHcm91cCkgaXMgdHJlYXRlZCBhcyBhIGNhdGVnb3JpY2FsIHZhcmlhYmxlIChkaXN0cmluY3QgZ3JvdXBzKSByYXRoZXIgdGhhbiBjb250aW51b3VzIG51bWVyaWNhbCB2YWx1ZXMNCg0KZGZfb3V0cHV0IDwtIGRhdGEuZnJhbWUoKSAjQ3JlYXRlIGFuIGVtcHR5IG1haW4gcmVzdWx0cyB0YWJsZQ0KZGZfb3V0cHV0X2xzbWVhbnMgPC0gZGF0YS5mcmFtZSgpICNDcmVhdGUgYW4gZW1wdHkgcmVzdWx0cyB0YWJsZSBmb3IgbGVhc3Qgc3F1YXJlZCBtZWFucw0KZGZfb3V0cHV0X3BhaXJ3aXNlIDwtIGRhdGEuZnJhbWUoKSAjQ3JlYXRlIGFuIGVtcHR5IHJlc3VsdHMgdGFibGUgZm9yIHRoZSBwYWlyd2F5IGNvbXBhcmlzb25zDQpgYGANCg0KVGhpcyBpcyB0aGUgbWFpbiAnbG9vcCcgb2YgdGhlIGNvZGUsIHdoaWNoIHJ1bnMgb25jZSBmb3IgZWFjaCBkZXBlbmRlbnQgdmFyaWFibGUgaW4gb3VyIHNwcmVhZHNoZWV0Lg0KDQpgYGB7cn0NCmZvcihkZXBlbmRlbnRfdmFyaWFibGUgaW4gbmFtZXMoaW5wdXRfZGF0YVssMzpuY29sKGlucHV0X2RhdGEpXSkpIHsgI1RoaXMgZm9yIGxvb3AgZW5zdXJlcyB0aGUgaW5kZW50ZWQgY29kZSBiZXR3ZWVuIHRoZSBjdXJseSBicmFjZXMgcnVucyBvbmNlIGZvciBlYWNoIGNvbHVtbiAoZGVwZW5kZW50IHZhcmlhYmxlKSBmcm9tIHRoZSAzcmQgY29sdW1uIG9ud2FyZHMNCiAgIyBTVEVQIDEuIEZpdCBhIE5PTi1IaWVyYXJjaGljYWwgbW9kZWwgKGVxdWl2YWxlbnQgdG8gYSB0LXRlc3QpDQogICNGaXQgYSBtb2RlbCwgY2FsY3VsYXRlIHRoZSBzdGFuZGFyZCBlcnJvciwgcCB2YWx1ZSwgYW5kIC0yIGxvZyBMaWtlbGlob29kDQogIE5PTl9oaWVyYXJjaGljYWxfbW9kZWwgPC0gZ2xtKGdldChkZXBlbmRlbnRfdmFyaWFibGUpIH4gQ29uZGl0aW9uLCBkYXRhPWlucHV0X2RhdGEpICNUaGlzIGZpdHMgYSBnZW5lcmxpc2VkIGxpbmVhciBtb2RlbCBmb3IgZWFjaCBkZXBlbmRlbnQgdmFyaWFibGUgYW5kIHRoZSBvdXRwdXQgY29sdW1uIChIRl9vcl9jb250cm9sOyB0aGUgcHJlc2VuY2Ugb3Igbm90IG9mIGhlYXJ0IGZhaWx1cmUpIGFuZCBzdG9yZXMgaXQgaW4gdGhlIHZhcmlhYmxlIG5hbWVkIE5PTl9oaWVyYXJjaGljYWxfbW9kZWwNCiAgc2VfTk9OX2hpZXJhcmNoaWNhbF9tb2RlbCA8LSBzdW1tYXJ5KE5PTl9oaWVyYXJjaGljYWxfbW9kZWwpJGNvZWZmaWNpZW50c1ssIDJdWzJdICNUaGlzIGV4dHJhY3RzIHRoZSBzdGFuZGFyZCBlcnJvcnMgZnJvbSB0aGUgbW9kZWwgYW5kIHN0b3JlcyBpdCBpbiBhIG5ldyB2YXJpYWJsZSBuYW1lZCBzZV9OT05faGllcmFyY2hpY2FsX21vZGVsDQogIHBfTk9OX2hpZXJhcmNoaWNhbF9tb2RlbCA8LSBzdW1tYXJ5KE5PTl9oaWVyYXJjaGljYWxfbW9kZWwpJGNvZWZmaWNpZW50c1ssIDRdWzJdICNUaGlzIGV4dHJhY3RzIHRoZSBzdGFuZGFyZCBlcnJvcnMgZnJvbSB0aGUgbW9kZWwgYW5kIHN0b3JlcyBpdCBpbiBhIG5ldyB2YXJpYWJsZSBuYW1lZCBwX05PTl9oaWVyYXJjaGljYWxfbW9kZWwNCiAgZ29vZG5lc3Nfb2ZfZml0X05PTl9oaWVyYXJjaGljYWxfbW9kZWwgPC0gbG9nTGlrKE5PTl9oaWVyYXJjaGljYWxfbW9kZWwpKi0yICNXZSBjYWxjdWxhdGUgYSBnb29kbmVzcyBvZiBmaXQgdXNpbmcgdGhlIC0yIGxvZ0xpa2VsaWhvb2Qgb2YgdGhlIG1vZGVsIGFuZCBzYXZlIGl0IGluIGEgbmV3IHZhcmlhYmxlIG5hbWVkIGdvb2RuZXNzX29mX2ZpdF9OT05faGllcmFyY2hpY2FsX21vZGVsDQogIA0KICAjIFNURVAgMi4gRml0IGEgSGllcmFjaGljYWwgbW9kZWwNCiAgIyBTdGVwIDJhLiBGaXQgYSBtb2RlbCwgY2FsY3VsYXRlIHRoZSBzdGFuZGFyZCBlcnJvciwgcCB2YWx1ZSBhbmQgLTIgbG9nIExpa2VsaWhvb2QNCiAgaGllcmFyY2hpY2FsX21vZGVsIDwtIGxtZXIoZ2V0KGRlcGVuZGVudF92YXJpYWJsZSkgfiBDb25kaXRpb24gKyAoMXxHcm91cCksIFJFTUw9RkFMU0UgLGRhdGE9aW5wdXRfZGF0YSkgI1dlIG5vdyBmaXQgYSBsaW5lYXIgbWl4ZWQgZWZmZWN0cyAoaGllcmFyY2hpY2FsKSBtb2RlbCBhbmQgc3RvcmUgdGhhdCBpbiB0aGUgdmFyaWFibGUgbmFtZWQgaGllcmFyY2hpY2FsX21vZGVsLiBXZSBmaXQgZWFjaCBkZXBlbmRlbnQgdmFyaWFibGUgYWdhaW5zdCB0aGUgT3V0cHV0IGNvbHVtbiwgd2l0aCAnUmF0JyBhcyBhIGdyb3VwIGVmZmVjdA0KICBzZV9oaWVyYXJjaGljYWxfbW9kZWwgPC0gc3VtbWFyeShoaWVyYXJjaGljYWxfbW9kZWwpJGNvZWZmaWNpZW50c1ssIDJdWzJdICNBcyBwcmV2aW91c2x5LCBleHRyYWN0IHRoZSBzdGFuZGFyZCBlcnJvcnMgYW5kIHN0b3JlIHRoZW0gaW4gYSB2YXJpYWJsZQ0KICBwX2hpZXJhcmNoaWNhbF9tb2RlbCA8LSBzdW1tYXJ5KGhpZXJhcmNoaWNhbF9tb2RlbCkkY29lZmZpY2llbnRzWyAsNV1bMl0gI0FzIHByZXZpb3VzbHksIGV4dHJhY3QgdGhlIHAgdmFsdWVzIGFuZCBzdG9yZSB0aGVtIGluIGEgdmFyaWFibGUNCiAgZ29vZG5lc3Nfb2ZfZml0X2hpZXJhcmNoaWNhbF9tb2RlbCA8LSBsb2dMaWsoaGllcmFyY2hpY2FsX21vZGVsKSotMiAjQXMgcHJldmlvdXNseSwgY2FsY3VsYXRlIGEgZ29vZG5lc3Mgb2YgZml0IGFuZCBzdG9yZSBpdCBpbiBhIHZhcmlhYmxlDQogIA0KICAjIFNURVAgMmIuIENhbGN1bGF0ZSBhbW91bnQgb2YgY2x1c3RlcmluZywgZGVmaW5lZCBhcyB0aGUgaW50cmFjbGFzcyBjb3JyZWxhdGlvbiwgYSB2YWx1ZSBiZXR3ZWVuIDAgYW5kIDEgDQogIHRhYmxlX29mX3ZhcmlhbmNlcyA8LSBhcy5kYXRhLmZyYW1lKFZhckNvcnIoaGllcmFyY2hpY2FsX21vZGVsKSkgI0NyZWF0ZSBhIGRhdGFmcmFtZSAodGFibGUpIG9mIHRoZSBjb3ZhcmlhbmNlIHBhcmFtZXRlcnMgY2FsbGVkICd0YWJsZV9vZl92YXJpYW5jZXMnDQogIHZhcmlhbmNlX29mX21lYW5zIDwtIHRhYmxlX29mX3ZhcmlhbmNlcyR2Y292WzFdICNFeHRyYWN0IHRoZSB2YXJpYW5jZSBvZiB0aGUgbWVhbiBmcm9tIHRoZSAndGFibGVfb2ZfdmFyaWFuY2VzJyBhbmQgc3RvcmUgaXQgaW4gYSB2YXJpYWJsZSBuYW1lcyAndmFyaWFuY2Vfb2ZfbWVhbnMnDQogIHZhcmlhbmNlX29mX2luZGl2aWR1YWxfZGF0YXBvaW50cyA8LSAodGFibGVfb2ZfdmFyaWFuY2VzJHZjb3ZbMV0gKyB0YWJsZV9vZl92YXJpYW5jZXMkdmNvdlsyXSkgI1RoZSB0b3RhbCB2YXJpYW5jZSBpcyBhbHNvIGV4dHJhY3RlZCwgYXMgZGVmaW5lZCBhcyB0aGUgdmFyaWFuY2Ugb2YgdGhlIHJhdCBtb2RlbCBwbHVzIHRoZSByZXNpZHVhbCB2YXJpYW5jZQ0KICBpY2MgPC0gdmFyaWFuY2Vfb2ZfbWVhbnMgLyB2YXJpYW5jZV9vZl9pbmRpdmlkdWFsX2RhdGFwb2ludHMgI1RoZSBpbnRyYS1jbGFzcyBjb3JyZWxhdGlvbiAoSUNDKSBpcyBjYWxjdWxhdGVkIGFuZCBkZWZpbmVkIGJ5IHRoZSB2YXJpYW5jZSBvZiB0aGUgbW9kZWwgZGl2aWRlZCBieSB0aGUgdG90YWwgdmFyaWFuY2UNCiAgDQogICMgU1RFUCAyYy4gQ2FsY3VsYXRlIGdvb2RuZXNzIG9mIGZpdCBhbmQgc2VlIGlmIGhpZ2hlciBmb3IgaGllcmFyY2hpY2FsIG1vZGVsDQogIGltcHJvdmVtZW50X2luX2dvb2RuZXNzX29mX2ZpdCA8LSBnb29kbmVzc19vZl9maXRfTk9OX2hpZXJhcmNoaWNhbF9tb2RlbCAtIGdvb2RuZXNzX29mX2ZpdF9oaWVyYXJjaGljYWxfbW9kZWwgI1dlIGNhbGN1bGF0ZSB0aGUgZGlmZmVyZW5jZSBiZXR3ZWVuIHRoZXByZXZpb3VzbHktY2FsY3VsYXRlZCBnb29kbmVzcyBvZiBmaXQgZm9yIGJvdGggdGhlIG5vbi1oaWVyYXJjaGljYWwgYW5kIGhpZXJyYWNoaWNhbCBtb2RlbHM7IHRoaXMgZGlmZmVyZW5jZSBpcyBzdG9yZWQgaW4gdGhlIHZhcmlhYmxlIGltcHJvdmVtZW50X2luX2dvb2RuZXNzX29mX2ZpdA0KICBiZXR0ZXJmaXQgPC0gMS1wY2hpc3EoaW1wcm92ZW1lbnRfaW5fZ29vZG5lc3Nfb2ZfZml0WzFdLGRmPTEpICNUaGUgcCB2YWx1ZSBmb3Igd2hldGhlciB0aGUgaGllcmFyY2hpY2FsIG1vZGVsIGlzIGEgc2lnbmlmaWNhbnQgaW1wcm92ZW1lbnQgaXMgY2FsY3VsYXRlZCB1c2luZyB0aGUgQ2hpLXNxdWFyZWQgZGlzdHJpYnV0aW9uIHdpdGggMSBkZWdyZWUgb2YgZnJlZWRvbQ0KICBwX2JldHRlcmZpdCA8LSBpZmVsc2UoYmV0dGVyZml0PDAuMDAwMSwiPDAuMDAwMSIscm91bmQoYmV0dGVyZml0LGRpZ2l0cz00KSkgI0lmIHRoZSBwIHZhbHVlIGNhbGN1bGF0ZWQgYWJvdmUgaXMgdmVyeSBzbWFsbCwgd2UganVzdCByZXBsYWNlIGl0IHdpdGggIjwwLjAwMDEiDQogIHN1cGVyaW9ycCA8LSBpZmVsc2UoaW1wcm92ZW1lbnRfaW5fZ29vZG5lc3Nfb2ZfZml0PjAgJiBiZXR0ZXJmaXQgPCAwLjA1LHBhc3RlKCJZICgiLCBwX2JldHRlcmZpdCAsIikiLHNlcD0iIikscGFzdGUoIk4gKCIsIGJldHRlcmZpdCwgIikiLHNlcD0iIikpICNJZiB0aGVyZSBpcyBhbiBpbXByb3ZlbWVudCBpbiB0aGUgZ29vZG5lc3Mgb2YgZml0IGFuZCB0aGUgcCB2YWx1ZSBpcyBsZXNzIHRoYW4gPDAuMDUsIHdlIHNldCB0aGUgdmFyaWFibGUgJ3N1cGVyaW9ycCcgdG8gIlkiLCBvdGhlcndpc2UgIk4iDQogIA0KICAjIFNURVAgMyBDYWxjdWxhdGUgbGVhc3Qgc3F1YXJlcyBtZWFucyBmb3IgZWFjaCBncm91cCBvZiB0aGUgb3V0Y29tZSB2YXJpYWJsZXMsIGFuZCB0aGUgcGFpcndpc2UgY29tcGFyaXNvbnMNCiAgbHNtZWFuIDwtIGxzbWVhbnNMVChoaWVyYXJjaGljYWxfbW9kZWwpICNXZSBjYWxjdWxhdGUgdGhlIGxlYXN0IHNxdWFyZXMgbWVhbnMsIHN0YW5kYXJkIGVycm9ycyBhbmQgY29uZmlkZW5jZSBpbnRlcnZhbHMgZm9yIHRoZSBkaWZmZXJlbnQgb3V0Y29tZXMgKGUuZy4gcHJlc2VuY2UgYW5kIGFic2Vuc2Ugb2YgaGVhcnQgZmFpbHVyZSkNCiAgcm93Lm5hbWVzKGxzbWVhbikgPC0gcGFzdGUoZGVwZW5kZW50X3ZhcmlhYmxlLHJvdy5uYW1lcyhsc21lYW4pLHNlcD0iIC0gIikgI0FkZCB0aGUgY3VycmVudCB2YXJpYWJsZSBpbiBxdWVzdGlvbiB0byB0aGUgcm93IG5hbWUNCiAgZGlmZmxzbWVhbiA8LSBkaWZmbHNtZWFucyhoaWVyYXJjaGljYWxfbW9kZWwpICNXZSB0ZXN0IGZvciBzaWduaWZpY2FuY2UgYmV0d2VlbiB0aGUgZGlmZmVyZW50IG91dGNvbWUgbGV2ZWxzDQogIHJvdy5uYW1lcyhkaWZmbHNtZWFuKSA8LSBwYXN0ZShkZXBlbmRlbnRfdmFyaWFibGUscm93Lm5hbWVzKGRpZmZsc21lYW4pLHNlcD0iIC0gIikgI0FkZCB0aGUgY3VycmVudCB2YXJpYWJsZSBpbiBxdWVzdGlvbiB0byB0aGUgcm93IG5hbWUNCiAgDQogIGRmX291dHB1dCA8LSByYmluZCggI1dlIGFkZCB0aGUgcmVzdWx0cyB0byBvdXIgbWFpbiByZXN1bHRzIHRhYmxlIGluIHRoaXMgZnVuY3Rpb24NCiAgICBkZl9vdXRwdXQsDQogICAgZGF0YS5mcmFtZSgNCiAgICAgIENvbW1vblNFPXJvdW5kKHNlX05PTl9oaWVyYXJjaGljYWxfbW9kZWwsZGlnaXRzPTMpLCAjQWRkIHRoZSBzdGFuZGFyZCBlcnJvciBvZiB0aGUgbm9uLWhpZXJhcmNoaWNhbCBtb2RlbA0KICAgICAgQ29tbW9ucD1pZmVsc2UocF9OT05faGllcmFyY2hpY2FsX21vZGVsPDAuMDAwMSwiPCAwLjAwMDEiLHRvU3RyaW5nKHJvdW5kKHBfTk9OX2hpZXJhcmNoaWNhbF9tb2RlbCxkaWdpdHM9NCkpKSwgI0FkZCB0aGUgcCB2YWx1ZSBvZiB0aGUgbm9uLWhpZXJhcmNoaWNhbCBtb2RlbDsgaWYgaXQncyB2ZXJ5IHNtYWxsLCByZXBsYWNlIGl0IHdpdGggIjwgMC4wMDAxIiINCiAgICAgIElDQz1wYXN0ZShyb3VuZChpY2MqMTAwLGRpZ2l0cz0xKSwiJSIsc2VwPSIiKSwgI0FkZCB0aGUgaW50cmFjbGFzcyBjb3JyZWxhdGlvbiBvZiB0aGUgaGllcmFyY2hpY2FsIG1vZGVsDQogICAgICBNaXhlZFNFPXJvdW5kKHNlX2hpZXJhcmNoaWNhbF9tb2RlbCxkaWdpdHM9MyksICNBZGQgdGhlIHN0YW5kYXJkIGVycm9yIG9mIHRoZSBoaWVyYXJjaGljYWwgbW9kZWwNCiAgICAgIE1peGVkcD1pZmVsc2UocF9oaWVyYXJjaGljYWxfbW9kZWw8MC4wMDAxLCI8IDAuMDAwMSIsdG9TdHJpbmcocm91bmQocF9oaWVyYXJjaGljYWxfbW9kZWwsZGlnaXRzPTQpKSksICNBZGQgdGhlIHAgdmFsdWUgb2YgdGhlIGhpZXJhcmNoaWNhbCBtb2RlbDsgaWYgaXQncyB2ZXJ5IHNtYWxsLCByZXBsYWNlIGl0IHdpdGggIjwgMC4wMDAxIiINCiAgICAgIFN1cGVyaW9yeW49c3VwZXJpb3JwICNBZGQgYSBjb2x1bW4gaW5kaWNhdGluZyBpZiB0aGUgaGllcmFyY2hpY2FsIG1vZGVsIGlzIGEgc3RhdGlzdGljYWxseSBzaWduaWZpY2FudGx5IGJldHRlciBmaXQgKFkgb3IgTikgYW5kIHRoZSBhY3R1YWwgcCB2YWx1ZQ0KICAgICkNCiAgKQ0KDQogIGRmX291dHB1dF9sc21lYW5zIDwtIHJiaW5kKCAjV2UgYWRkIHRoZSByZXN1bHRzIHRvIG91ciBscyBzcXVhcmVzIHJlc3VsdHMgdGFibGUgaW4gdGhpcyBmdW5jdGlvbg0KICAgIGRmX291dHB1dF9sc21lYW5zLA0KICAgIGxzbWVhbg0KICApDQogIA0KICBkZl9vdXRwdXRfcGFpcndpc2UgPC0gcmJpbmQoICNXZSBhZGQgdGhlIHJlc3VsdHMgdG8gb3VyIHBhaXJ3aXNlIGNvbXBhcmlzb25zIHJlc3VsdHMgdGFibGUgaW4gdGhpcyBmdW5jdGlvbg0KICAgIGRmX291dHB1dF9wYWlyd2lzZSwNCiAgICBkaWZmbHNtZWFuDQogICkNCiAgDQogIHJvdy5uYW1lcyhkZl9vdXRwdXQpW25yb3coZGZfb3V0cHV0KV0gPC0gZGVwZW5kZW50X3ZhcmlhYmxlICNTZXQgdGhlIG5hbWUgb2YgdGhlIHJvdyB0byB0aGUgZGVwZW5kZW50IHZhcmlhYmxlIGluIHF1ZXN0aW9ucw0KfQ0KYGBgDQoNCkZpbmFsbHksIHdlIHNwZWNpZnkgdGhlIGNvbHVtbiBoZWFkaW5ncyBoZXJlIGFuZCBwcmludCB0aGUgdGFibGUuDQoNCmBgYHtyfQ0KbmFtZXMoZGZfb3V0cHV0KSA9IGMoIkNvbW1vbiB0ZXN0IChTRSkiLCJDb21tb24gdGVzdCAocCkiLCJHcm91cC1sZXZlbCBjbHVzdGVyaW5nIChJQ0MpIiwiR3JvdXAtbGV2ZWwgKFNFKSIsIkdyb3VwLWxldmVsIChwKSIsIlN1cGVyaW9yIGZpdCAocCkiKSAjU2V0IHRoZSBjb2x1bW4gaGVhZGluZ3MNCnByaW50KGRmX291dHB1dCkgI091dHB1dCB0aGUgc3VtbWFyeSB0YWJsZQ0KYGBgDQoNCkhlcmUgd2Ugb3V0cHV0IGEgdGFibGUgY29udGFpbmluZyB0aGUgbGVhc3Qgc3F1YXJlcyBtZWFucyBhbmQgY29uZmlkZW5jZSBpbnRlcnZhbHMgZm9yIGVhY2ggb3V0Y29tZSwgZ3JvdXBlZCBieSBlYWNoIGRlcGVuZGVudCB2YXJpYWJsZS4NCg0KYGBge3J9DQpjb2xzLmRvbnQud2FudCA8LSBjKCJERiIsICJ0LXZhbHVlIiwgInAtdmFsdWUiKQ0KZGZfb3V0cHV0X2xzbWVhbnMgPC0gZGZfb3V0cHV0X2xzbWVhbnNbLCAhIG5hbWVzKGRmX291dHB1dF9sc21lYW5zKSAlaW4lIGNvbHMuZG9udC53YW50LCBkcm9wID0gRl0NCnByaW50KGRmX291dHB1dF9sc21lYW5zKSAjT3V0cHV0IHRoZSByZXN1bHRzIHRhYmxlIGZvciBMUyBtZWFucw0KYGBgDQoNCkhlcmUgd2Ugb3V0cHV0IHRoZSBwYWlyd2lzZSBjb21wYXJpc29ucy4NClAgdmFsdWVzIGFyZSBtdWx0aXBsaWVkIGJ5IHRoZSBudW1iZXIgb2YgY29tcGFyaXNvbnMgdG8gbWFpbnRhaW4gYSB0eXBlIDEgZXJyb3IgcmF0ZSBvZiA1JSAoQm9uZmVycm9uaSBjb3JyZWN0aW9uKQ0KVGhlIHJlc3VsdHMgYXJlIGdyb3VwZWQgZm9yIGVhY2ggb3V0cHV0IHZhcmlhYmxlIChsZWZ0IGNvbHVtbiksIHdpdGggZWFjaCBjb21iaW5hdGlvbiBvZiBvdXRjb21lcyB1bmRlcmdvaW5nIHNpZ25pZmljYW5jZSB0ZXN0aW5nLg0KDQpgYGB7cn0NCmNvbHMuZG9udC53YW50IDwtIGMoIkRGIiwgInQtdmFsdWUiLCAiTG93ZXIgQ0kiLCJVcHBlciBDSSIpDQpkZl9vdXRwdXRfcGFpcndpc2UgPC0gZGZfb3V0cHV0X3BhaXJ3aXNlWywgISBuYW1lcyhkZl9vdXRwdXRfcGFpcndpc2UpICVpbiUgY29scy5kb250LndhbnQsIGRyb3AgPSBGXSAjUmVtb3ZlIHRoZSB1bndhbnRlZCBoZWFkaW5ncyBmcm9tIHRoZSB0YWJsZQ0KZGZfb3V0cHV0X3BhaXJ3aXNlJGBwLXZhbHVlYCA8LSBkZl9vdXRwdXRfcGFpcndpc2UkYFByKD58dHwpYCAqICggbnJvdyhkZl9vdXRwdXRfcGFpcndpc2UpIC8gbGVuZ3RoKG5hbWVzKGlucHV0X2RhdGFbLDM6bmNvbChpbnB1dF9kYXRhKV0pKSApICNNdWx0aXBseSB0aGUgcCB2YWx1ZXMgYnkgdGhlIG51bWJlciBvZiBjb21wYXJpc29ucyBwZXIgZGVwZW5kZW50IHZhcmlhYmxlDQpuYW1lcyhkZl9vdXRwdXRfcGFpcndpc2UpW25hbWVzKGRmX291dHB1dF9wYWlyd2lzZSk9PSJwLXZhbHVlIl0gPC0gIkJvbmZlcnJvbmkgcC12YWx1ZSIgI0NoYW5nZSB0aGUgY29sdW1uIGhlYWRpbmcNCmRmX291dHB1dF9wYWlyd2lzZVtkZl9vdXRwdXRfcGFpcndpc2UkYEJvbmZlcnJvbmkgcC12YWx1ZWAgPiAxLCJCb25mZXJyb25pIHAtdmFsdWUiXSA8LSAxICNDaGFuZ2UgYW55IHAgdmFsdWUgYWJvdmUgMSB0byAxDQpwcmludChkZl9vdXRwdXRfcGFpcndpc2UpICNPdXRwdXQgdGhlIHJlc3VsdHMgdGFibGUgZm9yIHRoZSBwYWlyd2lzZSBjb21wYXJpc29ucw0KYGBg
