# Supplementary material for: Hierarchical statistical techniques are necessary to draw reliable conclusions from analysis of isolated cardiomyocyte studies
Source: Cardiovasc Res. 2017 Aug 30;113(14):1743–52. doi: 10.1093/cvr/cvx151 (PMC5852514; doi:10.1093/cvr/cvx151)
Supplement: Supplementary Data [file cvx151_suppl_data.zip › ratonly.pdf]

R Notebook - Rat-level hierarchical analysis

This is an R Markdown Notebook,

The source code has been separated into 'chunks' which can be run step-wise

---

How to use this script (Note green text indicates a comment rather than code)

1. Ensure the lmerTest package is installed; if unsure, type install.packages('lmerTest'); if using excel input files, you will also need to run install.packages('readxl')
2. Set your working directory in your R program to the location of your input file (which does not necessarily need to be the location of this script) - In R studio this is done by going into 'Session -> Set Working Directory'
3. Either ensure your input file is named "Hierarchical Transient analysis with Rat-Level Clustering.xlsx", or replace the code on line 50 with the appropriate filename
4. Run this file using 'Run -> Run all'

If readers would like other pragmatic examples in using mixed effect models for Hierarchical statistics, we would recommend https://www.jaredknowles.com/journal/2013/11/25/getting-started-with-mixed-effect-models-in-r

This is a PATCHED version of the original source code as of 07/08/2018 which works on the latest version of R and lmerTest.

Please contact james@jph.am for any questions

THE PROGRAM STARTS HERE

Here we load the packages we require for the analysis:

```
library(lmerTest) #If told 'there is no package called 'lmerTest', run 'install.packages("lmerTest")'
```

```
## Warning: package 'lmerTest' was built under R version 3.4.4

## Loading required package: lme4

## Warning: package 'lme4' was built under R version 3.4.4

## Loading required package: Matrix

##
## Attaching package: 'lmerTest'

## The following object is masked from 'package:lme4':
##
##     lmer

## The following object is masked from 'package:stats':
##
##     step
```

```
require(readxl) #If told 'there is no package called 'readxl', run 'install.packages("readxl")'
```

```
## Loading required package: readxl
```

This prevents scientific notation for p values unless they are very small.

```
options(scipen=999)
```

Now we load the excel spreadsheet into the variable 'input data'.

```
input_data <- read_excel("Hierarchical Transient analysis with Rat-Level Clustering.xlsx") #This loads
```

Here we rename the column of our condition (e.g. heart failure or not) to 'Condition', and the grouping variable (e.g. rat) to 'Group'.

We then ensure that the condition and group columns are treated as categorical variables (factors) rather than a continuous numerical value.

Finally, we create an empty results table.

```
names(input_data)[1] <- "Condition"
names(input_data)[2] <- "Group"

input_data[1] <- as.factor(unlist(input_data[1])) #Ensure the Condition group is treated as a categoric
input_data$Group <- factor(input_data$Group) #Ensure the Rat group (Group) is treated as a categorical

df_output <- data.frame() #Create an empty main results table
df_output_lsmeans <- data.frame() #Create an empty results table for least squared means
df_output_pairwise <- data.frame() #Create an empty results table for the pairway comparisons
```

This is the main 'loop' of the code, which runs once for each dependent variable in our spreadsheet.

```
for(dependent_variable in names(input_data[,3:ncol(input_data)])) { #This for loop ensures the indented
  # STEP 1. Fit a NON-Hierarchical model (equivalent to a t-test)
  #Fit a model, calculate the standard error, p value, and -2 log Likelihood
  NON_hierarchical_model <- glm(get(dependent_variable) ~ Condition, data=input_data) #This fits a gene
  se_NON_hierarchical_model <- summary(NON_hierarchical_model)$coefficients[, 2][2] #This extracts the
  p_NON_hierarchical_model <- summary(NON_hierarchical_model)$coefficients[, 4][2] #This extracts the s
  goodness_of_fit_NON_hierarchical_model <- logLik(NON_hierarchical_model)*-2 #We calculate a goodness

  # STEP 2. Fit a Hierachical model
  # Step 2a. Fit a model, calculate the standard error, p value and -2 log Likelihood
  hierarchical_model <- lmer(get(dependent_variable) ~ Condition + (1|Group), REML=FALSE ,data=input_da
  se_hierarchical_model <- summary(hierarchical_model)$coefficients[, 2][2] #As previously, extract the
  p_hierarchical_model <- summary(hierarchical_model)$coefficients[ ,5][2] #As previously, extract the
  goodness_of_fit_hierarchical_model <- logLik(hierarchical_model)*-2 #As previously, calculate a goodn

  # STEP 2b. Calculate amount of clustering, defined as the intraclass correlation, a value between 0 a
  table_of_variances <- as.data.frame(VarCorr(hierarchical_model)) #Create a dataframe (table) of the c
  variance_of_means <- table_of_variances$vcov[1] #Extract the variance of the mean from the 'table_of_
  variance_of_individual_datapoints <- (table_of_variances$vcov[1] + table_of_variances$vcov[2]) #The t
  icc <- variance_of_means / variance_of_individual_datapoints #The intra-class correlation (ICC) is ca

  # STEP 2c. Calculate goodness of fit and see if higher for hierarchical model
  improvement_in_goodness_of_fit <- goodness_of_fit_NON_hierarchical_model - goodness_of_fit_hierarchic
  betterfit <- 1-pchisq(improvement_in_goodness_of_fit[1],df=1) #The p value for whether the hierarchic
  p_betterfit <- ifelse(betterfit<0.0001,"<0.0001",round(betterfit,digits=4)) #If the p value calculate
  superiorp <- ifelse(improvement_in_goodness_of_fit>0 & betterfit < 0.05,paste("Y (", p_betterfit ,")"

  # STEP 3 Calculate least squares means for each group of the outcome variables, and the pairwise comp
  lsmean <- lsmeansLT(hierarchical_model) #We calculate the least squares means, standard errors and co
  row.names(lsmean) <- paste(dependent_variable,row.names(lsmean),sep=" - ") #Add the current variable
```

```
  difflsmean <- difflsmeans(hierarchical_model) #We test for significance between the different outcome
  row.names(difflsmean) <- paste(dependent_variable,row.names(difflsmean),sep=" - ") #Add the current v

  df_output <- rbind( #We add the results to our main results table in this function
    df_output,
    data.frame(
      CommonSE=round(se_NON_hierarchical_model,digits=3), #Add the standard error of the non-hierarchic
      Commonp=ifelse(p_NON_hierarchical_model<0.0001,"< 0.0001",toString(round(p_NON_hierarchical_model
      ICC=paste(round(icc*100,digits=1),"%",sep=""), #Add the intraclass correlation of the hierarchica
      MixedSE=round(se_hierarchical_model,digits=3), #Add the standard error of the hierarchical model
      Mixedp=ifelse(p_hierarchical_model<0.0001,"< 0.0001",toString(round(p_hierarchical_model,digits=4
      Superioryn=superiorp #Add a column indicating if the hierarchical model is a statistically signif
    )
  )

  df_output_lsmeans <- rbind( #We add the results to our ls squares results table in this function
    df_output_lsmeans,
    lsmean
  )

  df_output_pairwise <- rbind( #We add the results to our pairwise comparisons results table in this fu
    df_output_pairwise,
    difflsmean
  )

  row.names(df_output)[nrow(df_output)] <- dependent_variable #Set the name of the row to the dependent
}
```

Finally, we specify the column headings here and print the table.

```
names(df_output) = c("Common test (SE)","Common test (p)","Group-level clustering (ICC)","Group-level (
print(df_output) #Output the summary table
```

```
##                      Common test (SE) Common test (p)
## Transient Amplitude             0.031        < 0.0001
## Baseline                        0.012          0.2476
## Peak                            0.040          0.0042
## Tto50%Peak                      0.001          0.0178
## Tto50%decay                     0.005          0.4433
## Tau                             0.009          0.5351
##                      Group-level clustering (ICC) Group-level (SE)
## Transient Amplitude                         20.9%            0.049
## Baseline                                    27.1%            0.020
## Peak                                        22.6%            0.065
## Tto50%Peak                                  12.3%            0.001
## Tto50%decay                                   44%            0.010
## Tau                                         46.5%            0.018
##                      Group-level (p) Superior fit (p)
## Transient Amplitude           0.0101       Y (0.0057)
## Baseline                      0.6232      Y (<0.0001)
## Peak                          0.0464       Y (0.0017)
## Tto50%Peak                    0.1092       Y (0.0207)
## Tto50%decay                   0.3998      Y (<0.0001)
## Tau                           0.4238      Y (<0.0001)
```

Here we output a table containing the least squares means and confidence intervals for each outcome, grouped by each dependent variable.

```
cols.dont.want <- c("DF", "t-value", "p-value")
df_output_lsmeans <- df_output_lsmeans[, ! names(df_output_lsmeans) %in% cols.dont.want, drop = F]
print(df_output_lsmeans) #Output the results table for LS means
```

```
##                                    Estimate Std. Error   df  t value
## Transient Amplitude - Condition0 1.38580799 0.03484998 14.6  39.765
## Transient Amplitude - Condition1 1.53158208 0.03480867 14.3  44.000
## Baseline - Condition0            1.06298741 0.01436812 20.5  73.982
## Baseline - Condition1            1.05285216 0.01436224 20.3  73.307
## Peak - Condition0                1.47458245 0.04620646 15.8  31.913
## Peak - Condition1                1.61588544 0.04616393 15.5  35.003
## Tto50%Peak - Condition0          0.02650272 0.00050711 18.3  52.262
## Tto50%Peak - Condition1          0.02770918 0.00050527 17.6  54.840
## Tto50%decay - Condition0         0.09760852 0.00694241 19.6  14.060
## Tto50%decay - Condition1         0.08915714 0.00694445 19.6  12.839
## Tau - Condition0                 0.14715677 0.01248907 19.6  11.783
## Tau - Condition1                 0.13272588 0.01249307 19.6  10.624
##                                       lower      upper
## Transient Amplitude - Condition0 1.31134537 1.46027061
## Transient Amplitude - Condition1 1.45708901 1.60607515
## Baseline - Condition0            1.03306339 1.09291143
## Baseline - Condition1            1.02292228 1.08278203
## Peak - Condition0                1.37650689 1.57265801
## Peak - Condition1                1.51777773 1.71399315
## Tto50%Peak - Condition0          0.02543855 0.02756688
## Tto50%Peak - Condition1          0.02664602 0.02877235
## Tto50%decay - Condition0         0.08311023 0.11210681
## Tto50%decay - Condition1         0.07465316 0.10366112
## Tau - Condition0                 0.12107243 0.17324112
## Tau - Condition1                 0.10663147 0.15882029
##                                                Pr(>|t|)
## Transient Amplitude - Condition0 0.0000000000000002749 ***
## Transient Amplitude - Condition1 < 0.00000000000000022 ***
## Baseline - Condition0            < 0.00000000000000022 ***
## Baseline - Condition1            < 0.00000000000000022 ***
## Peak - Condition0                0.0000000000000009597 ***
## Peak - Condition1                0.0000000000000003415 ***
## Tto50%Peak - Condition0          < 0.00000000000000022 ***
## Tto50%Peak - Condition1          < 0.00000000000000022 ***
## Tto50%decay - Condition0         0.0000000000103350884 ***
## Tto50%decay - Condition1         0.0000000000532026194 ***
## Tau - Condition0                 0.0000000002381886041 ***
## Tau - Condition1                 0.0000000014110491745 ***
## ---
## Signif. codes:  0 '***' 0.001 '**' 0.01 '*' 0.05 '.' 0.1 ' ' 1
```

Here we output the pairwise comparisons. P values are multiplied by the number of comparisons to maintain a type 1 error rate of 5% (Bonferroni correction) The results are grouped for each output variable (left column), with each combination of outcomes undergoing significance testing.

```
cols.dont.want <- c("DF", "t-value", "Lower CI","Upper CI")
df_output_pairwise <- df_output_pairwise[, ! names(df_output_pairwise) %in% cols.dont.want, drop = F] #
df_output_pairwise$`p-value` <- df_output_pairwise$`Pr(>|t|)` * ( nrow(df_output_pairwise) / length(nam
```

```r
names(df_output_pairwise)[names(df_output_pairwise)=="p-value"] <- "Bonferroni p-value" #Change the col
df_output_pairwise[df_output_pairwise$`Bonferroni p-value` > 1,"Bonferroni p-value"] <- 1 #Change any p
print(df_output_pairwise) #Output the results table for the pairwise comparisons
```

```
##                                                Estimate  Std. Error   df
## Transient Amplitude - Condition0 - Condition1 -0.14577409  0.04925611 14.5
## Baseline - Condition0 - Condition1             0.01013526  0.02031543 20.4
## Peak - Condition0 - Condition1                -0.14130299  0.06531574 15.6
## Tto50%Peak - Condition0 - Condition1          -0.00120647  0.00071586 18.0
## Tto50%decay - Condition0 - Condition1          0.00845138  0.00981949 19.6
## Tau - Condition0 - Condition1                  0.01443089  0.01766504 19.6
##                                               t value       lower
## Transient Amplitude - Condition0 - Condition1 -2.9595 -0.25110137
## Baseline - Condition0 - Condition1             0.4989 -0.03218782
## Peak - Condition0 - Condition1                -2.1634 -0.28002526
## Tto50%Peak - Condition0 - Condition1          -1.6853 -0.00271070
## Tto50%decay - Condition0 - Condition1          0.8607 -0.01205633
## Tau - Condition0 - Condition1                  0.8169 -0.02246506
##                                                     upper Pr(>|t|)
## Transient Amplitude - Condition0 - Condition1 -0.04044681 0.010056
## Baseline - Condition0 - Condition1             0.05245833 0.623189
## Peak - Condition0 - Condition1                -0.00258072 0.046360
## Tto50%Peak - Condition0 - Condition1           0.00029777 0.109225
## Tto50%decay - Condition0 - Condition1          0.02895909 0.399807
## Tau - Condition0 - Condition1                  0.05132684 0.423790
##                                               Bonferroni p-value
## Transient Amplitude - Condition0 - Condition1           0.01006 *
## Baseline - Condition0 - Condition1                      0.62319
## Peak - Condition0 - Condition1                          0.04636 *
## Tto50%Peak - Condition0 - Condition1                    0.10922
## Tto50%decay - Condition0 - Condition1                   0.39981
## Tau - Condition0 - Condition1                           0.42379
## ---
## Signif. codes:  0 '***' 0.001 '**' 0.01 '*' 0.05 '.' 0.1 ' ' 1
```
