# Supplementary material for: Hierarchical statistical techniques are necessary to draw reliable conclusions from analysis of isolated cardiomyocyte studies
Source: Cardiovasc Res. 2017 Aug 30;113(14):1743–52. doi: 10.1093/cvr/cvx151 (PMC5852514; doi:10.1093/cvr/cvx151)
Supplement: Supplementary Data [file cvx151_suppl_data.zip › Stats Manuscript Supplement.docx]

#### **Supplementary Methods - Further Detail on Hierarchical Statistical Techniques Used**

Hierarchical statistical methodologies have been reviewed extensively^1^. In our data set multiple cells come from each individual rat heart.

The statistical model most appropriate for the data analysed in this study is the random intercept model. This is the simplest form of hierarchical model, but because the data structure is also very simple with only a single predictor (i.e. absence or presence of HF) and data within a single isolation having HF state in common (i.e. we do not have data from the same rat before and after HF), it is also the most appropriate in this setting. The random intercept model is a modification of the conventional equation for linear regression:

$$Y_{i}=b_{0}+b_{1}X_{i}+\varepsilon_{i}$$

Equation 1. Conventional linear regression

Where Y is the outcome measure, b_0_ is the intercept , b_1_ is a coefficient, X is the predictor variable and ε is the error term. Y, X and ε all vary as a function of i which represents an individual case of data. In our data Y is an outcome measure in terms of SR Ca^2+^ release quantification (e.g. transient amplitude). For the current study X represents the absence or presence of HF and is thus classified as a “dummy-coded” variable^2^, in other words, despite this being a linear regression, HF is a predictor variable which can only hold the values of 0 or 1. Since HF is always the predictor in this study we will place it within the linear regression equation:

$$Y_{i}=b_{0}+b_{1}{HF}_{i}+\varepsilon_{i}$$

Equation 2. Linear regression including HF as a parameter

A random intercept model means that for each subject at the highest level of the hierarchy, there can be a different intercept. In the current data set that means that we do not necessarily expect cells from each heart to have the same mean transient amplitude. Graphically this is shown in Figure S2. Including a random intercept requires a modification to the equation for standard linear regression as follows:

$$Y_{ij}=b_{0}+u_{0j}+b_{1}{HF}_{ij}+\varepsilon_{ij}$$

Equation 3. The basic random intercept model

Each outcome measure now varies with both specific case (i) and which isolation that case originates from (j). The additional term u_0j_ is the term which models the variation of the intercept for each isolation from the average intercept (b_0_).

The levels of hierarchy differed for transient and spark analysis. For transient analysis there was a single data point collected per cell (averaged transient amplitude or morphological parameters since each evoked Ca^2+^ amplitude has very similar characteristics). As such this is a two-level hierarchy, with the upper level being the isolation and the lower level the cells (represented in Equation 3). For spark morphological parameters there was a three-level hierarchy with isolations at the highest level, cells at the intermediate level and spark parameters at the lowest level of the hierarchy. This requires a modification to the equation:

$$Y_{ijk}=b_{0}+u_{0j}+v_{0jk}+b_{1}{HF}_{ijk}+\varepsilon_{ijk}$$

Equation 4. Three level random intercept model

In Equation 4 *u_0j_* represents the random intercept for spark parameters for that isolation and *v_0jk_* represents the additional adjustment in intercept made for the specific cell. The other measures have subscript “ijk” because they represent the value of the parameter for the individual spark (i) which is within its individual isolation (j) and cell (k).

Method for SPSS analysis

A syntax for 2-level hierarchical model was written in SPSS as follows. This produces a 2 level random intercept model nested in isolations. In this example Transient amplitude (TransAmp) is the parameter analysed. To compare other transient parameters in the same way, change “TransAmp” to the name of the other parameter (e.g. “Tau”) – everything else can remain as it is:

MIXED TransAmp BY HF

/CRITERIA=CIN(95) MXITER(100) MXSTEP(10) SCORING(1) SINGULAR(0.000000000001) HCONVERGE(0,

ABSOLUTE) LCONVERGE(0, ABSOLUTE) PCONVERGE(0.000001, ABSOLUTE)

/FIXED=HF | SSTYPE(3)

/METHOD=ML

/PRINT=SOLUTION TESTCOV

/RANDOM=INTERCEPT | SUBJECT(Isolation) COVTYPE(UN)

/EMMEANS=TABLES(OVERALL)

/EMMEANS=TABLES(HF) COMPARE ADJ(LSD).

In the same way a syntax for 3-level hierarchical model was written in SPSS as follows. This produces a 3 level random intercept model nested in cells nested within isolations. In this example LogAmp is the parameter analysed:

MIXED LogAmp BY HF

/CRITERIA=CIN(95) MXITER(100) MXSTEP(10) SCORING(1) SINGULAR(0.000000000001) HCONVERGE(0, ABSOLUTE) LCONVERGE(0, ABSOLUTE) PCONVERGE(0.000001, ABSOLUTE)

/FIXED=HF | SSTYPE(3)

/METHOD=ML

/PRINT=R SOLUTION TESTCOV

/RANDOM=INTERCEPT | SUBJECT(Isolation) COVTYPE(ID)

/RANDOM=INTERCEPT | SUBJECT(Cell*Isolation) COVTYPE(ID)

/SAVE=PRED RESID

/EMMEANS=TABLES(HF) COMPARE ADJ(LSD).

Method for Calculating Intraclass Correlation (ICC)

As part of the outputs from a mixed model, estimates of Covariance parameters are given e.g.


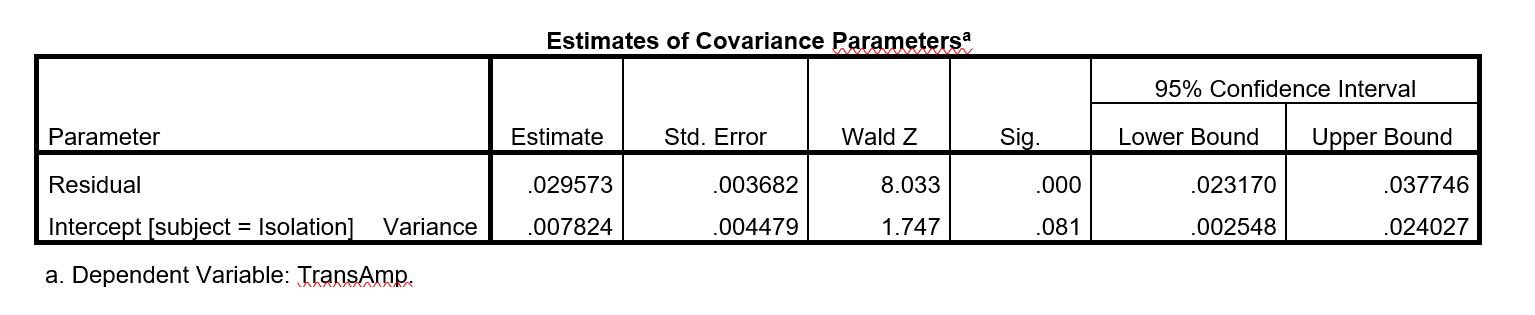


To calculate ICC we have taken the Estimate of the covariance parameter (ECP) for the intercept and then divide by the ECP for intercept+ECP for residual. In the case above this 0.0078/(0.0078+0.0295) =21%.s

| Publication | Wks Post MI | Spontaneous Ca^2+^ Spark Frequency | | | Ca^2+^ Spark Amplitude | | | Ca^2+^ Spark Width | | | Ca2+ Spark Duration | | |
| --- | --- | --- | --- | --- | --- | --- | --- | --- | --- | --- | --- | --- | --- |
|  |  | ↓ | ↔ | ↑ | ↓ | ↔ | ↑ | ↓ | ↔ | ↑ | ↓ | ↔ | ↑ |
| Huang *et al.*, 2008 | 6 |  |  |  | ✓ |  |  |  |  |  |  |  |  |
| Lee *et al.*, 2009 | 7 |  |  | ✓ |  | ✓ |  |  |  | ✓ |  | ✓ |  |
| Ibrahim *et al.*, 2012 | 12 |  |  | ✓ |  |  | ✓ |  |  | ✓ |  |  | ✓ |
| Lyon *et al.*, 2009 | 16 |  |  | ✓ |  |  | ✓ |  |  |  |  |  |  |
| Lyon *et al.*, 2011 | 16 |  |  | ✓ |  |  |  |  |  |  |  |  |  |
| Gomez *et al.*, 2001 | 24 |  |  |  |  | ✓ |  |  |  |  |  | ✓ |  |
| TOTAL |  | 0 | 0 | 4 | 1 | 2 | 2 | 0 | 0 | 2 | 0 | 2 | 1 |
| TOTAL (%) |  | 0 | 0 | 100 | 20 | 40 | 40 | 0 | 0 | 100 | 0 | 67 | 33 |

Table S1. Summary of changes in spark parameters in rat models of post MI HF.

###### Ticks refer to whether spark parameters are significantly reduced (↓), the same (↔) or increased (↑) in myocytes isolated from post MI animals compared with control animals.

| Publication | Wks Post MI | Ca^2+^ Transient Amplitude | | | Transient Decay Time | | | SR Ca^2+^ content | | | Diastolic [Ca^2+^]_i_ | | |
| --- | --- | --- | --- | --- | --- | --- | --- | --- | --- | --- | --- | --- | --- |
|  |  | ↓ | ↔ | ↑ | ↓ | ↔ | ↑ | ↓ | ↔ | ↑ | ↓ | ↔ | ↑ |
| Mørk *et al.*, 2007 | 1 |  |  | ✓ | ✓ |  |  |  |  | ✓ |  |  |  |
| Louch *et al.*, 2006 | 3 |  |  | ✓ |  |  |  |  |  |  |  |  |  |
| Zalvidea *et al.*, 2012 | 6 | ✓ |  |  |  |  | ✓ | ✓ |  |  |  |  | ✓ |
| Bito *et al.*, 2013 | 7 |  | ✓ |  |  |  |  |  |  |  |  | ✓ |  |
| Bito *et al.*, 2010 | 8 |  | ✓ |  |  |  | ✓ |  | ✓ |  |  | ✓ |  |
| Louch *et al.*, 2010 | 10 |  |  | ✓ |  |  |  |  |  |  |  |  |  |
| Mørk *et al.*, 2009 | 10 |  |  | ✓ |  | ✓ |  |  |  | ✓ |  |  |  |
| TOTAL |  | 1 | 2 | 4 | 1 | 1 | 2 | 1 | 1 | 2 | 0 | 2 | 1 |
| TOTAL (%) |  | 14 | 29 | 57 | 25 | 25 | 50 | 25 | 25 | 50 | 0 | 67 | 33 |

Table S2. Summary of changes in electrically evoked Ca^2+^ transients and SR load assessment in mouse models of post MI HF.

###### Ticks refer to whether parameters (evoked Ca^2+^ transient amplitude and decay time, SR Ca^2+^ content and diastolic [Ca^2+^]_i_) are significantly reduced (↓), the same (↔) or increased (↑) in myocytes isolated from post MI animals compared with control animals.

| Publication | Wks Post MI | Spontaneous Ca^2+^ Spark Frequency | | | Ca^2+^ Spark Amplitude | | | Ca^2+^ Spark Width | | | Ca2+ Spark Duration | | |
| --- | --- | --- | --- | --- | --- | --- | --- | --- | --- | --- | --- | --- | --- |
|  |  | ↓ | ↔ | ↑ | ↓ | ↔ | ↑ | ↓ | ↔ | ↑ | ↓ | ↔ | ↑ |
| Thireau *et al.*, 2012 | 4 |  |  | ✓ |  | ✓ |  |  |  |  |  |  |  |
| Bito *et al.*, 2010 | 8 |  | ✓ |  |  |  |  |  |  |  |  |  |  |
| Louch *et al.*, 2013 | 10 |  |  | ✓ |  |  | ✓ |  | ✓ |  |  |  | ✓ |
| TOTAL |  | 0 | 1 | 2 | 0 | 1 | 1 | 0 | 1 | 0 | 0 | 0 | 1 |

Table S3. Summary of changes in spark parameters in rat models of post MI HF.

###### Ticks refer to whether spark parameters are significantly reduced (↓), the same (↔) or increased (↑) in myocytes isolated from post MI animals compared with control animals.

|  | | | | | | |
| --- | --- | --- | --- | --- | --- | --- |
| Parameter | t | df | Sig. (2-tailed) | Mean Difference | Std. Error Difference |  |
| Diastolic Ca^2+^ (ratio 360/380nm) |  |  |  |  |  |  |
|  | .371 | 17.996 | 0.715 | 0.00781 | 0.0210 |  |
| Systolic Ca^2+^ (ratio 360/380nm) | -2.11 | 18 | *** 0.049** | -0.161 | 0.0763 |  |
|  |  |  |  |  |  |  |
| Trasient Amplitude (Systolic/Diastolic Ratio) | -2.70 | 18 | ***0.015** | -0.160 | 0.0593 |  |
|  |  |  |  |  |  |  |
| Time to 50% peak amplitude (ms) | -1.36 | 18 | 0.190 | -0.00108 | 0.000790 |  |
|  |  |  |  |  |  |  |
| Time to 50% decay (ms) | .953 | 18 | 0.353 | 0.00997 | 0.0105 |  |
|  |  |  |  |  |  |  |
| Tau (ms) | .903 | 18 | 0.378 | 0.0170 | 0.0188 |  |
|  |  |  |  |  |  |  |

Table S4. Analysis of aggregated data for Ca^2+^ transient parameters using independent samples t-test.

###### Aggregated results show similar findings to the 2 level hierarchical analysis for parameters of Ca^2+^ transient morphology. Both peak systolic Ca^2+^and as a result Ca^2+^ amplitude are significantly increased in HF. There is some overestimation of standard error compared to the hierarchical model leading to higher p values.

| Parameter | t | df | Sig. (2-tailed) | Mean Difference | Std. Error Difference |
| --- | --- | --- | --- | --- | --- |
| LogAmp (ΔF/F_0_) | -.549 | 10 | .595 | -.0432 | .0787 |
| LogFWHM ( | -.044 | 10 | .965 | -.00093 | .0209 |
| LogFDHM_mean | -.205 | 10 | .841 | -.0111 | .0542 |
| SparkFreq | -1.21 | 10 | .256 | -.704 | .584 |

Table S5. Analysis of aggregated data for Ca^2+^ spark parameters using independent samples t-test.

###### Aggregated results show similar findings to the 2 and 3 level hierarchical analyses for parameters of Ca^2+^ spark frequency and morphological parameters although similar to the transient parameters there is some overestimation of standard error compared to the hierarchical models leading to higher p values.

|  | Clustering of data (ICC) | Common Test  of HF vs Control | |  | Hierarchical Test  of HF vs Control | |  | Comparison of goodness of fit (common vs hierarchical) |
| --- | --- | --- | --- | --- | --- | --- | --- | --- |
|  |  | Estimated Mean and 95% CI Control | Estimated Mean and 95% CI HF | | Estimated Mean and 95% CI Control | Estimated Mean and 95% CI HF |  |  |
| Diastolic ratio | 27% | 1.056  (1.039-1.073) | 1.042  (1.026-1.059) | | 1.062  (1.033-1.093) | 1.053  (1.023-1.083) | | <0.001 *** |
| Peak systolic ratio | 23% | 1.468  (1.412-1.523) | 1.583  (1.529-1.638) | | 1.475  (1.377-1.573) | 1.616  (1.518-1.714) | | 0.002 ** |
| Transient amplitude (F/F_0_) | 21% | 1.388  (1.345-1.431) | 1.517  (1.475-1.559) | | 1.386  (1.311-1.460) | 1.532  (1.457-1.606) | | 0.006 ** |
| Time to 50% Peak (ms) | 12% | 26.61  (25.88-27.34) | 27.85  (27.14-28.57) | | 26.50  (25.43-27.56) | 27.70  (26.65-28.77) | | 0.021 * |
| Time to 50% Decay | 44% | 94.79  (87.98-101.6) | 91.06  (84.38-97.73) | | 97.61  (83.11-112.1) | 89.16  (74.65-103.7) | | <0.001 *** |
| Tau | 47% | 141.6  (129.7-153.6) | 136.3  (124.6-148.1) | | 147.1  (121.1-173.2) | 132.7  (106.6-158.8) | | <0.001 *** |

Table S6. Estimated means and confidence intervals for Ca^2+^ transient morphology variables using standard and hierarchical statistical tests.

###### The independent-samples t-test is shown as the common test used to compare cellular data. The clustering of data measured by calculating the intraclass correlation (ICC) is shown for each variable. The hierarchical technique is more appropriate with each variable as indicated by better goodness of fit (as measured by χ^2^-2LL test). When using the more appropriate hierarchical test the mean (point estimate) changes little but the estimate is appropriately less precise (wider 95% confidence interval). CI = Confidence Interval.

|  | Clus-tering of Data (ICC) | Common Method | | 2-level Hierarchy | | 3-level Hierarchy | | Comparison of goodness of fit (common vs hierarchical) |
| --- | --- | --- | --- | --- | --- | --- | --- | --- |
|  |  | Estimated Mean and 95% CI Control | Estimated Mean and 95% CI HF | Estimated Mean and 95% CI Control | Estimated Mean and 95% CI HF | Estimated Mean and 95% CI Control | Estimated Mean and 95% CI HF |  |
| Variables that have a single value per *cell*, and have clustering within *animal*. | | | | | | | | |
| Spark Freq  (Sp/100µm/s) | 24% | 1.971 (1.18-2.77) | 2.049 (1.35-2.75) | 1.805 (0.81-2.82) | 2.221 (1.20-3.24) | N/A | N/A | 0.048 * |
| Variables that have a single value per *spark,* and show clustering within *cell* and within *animal.* | | | | | | | | |
| LogAmp  (ΔF/F_0_) | 58% | -0.218 (-0.23 - -0.21) | -0.134 (-0.15 - -0.12) | -0.219 (-0.27 - -0.17) | 0.104 (-0.15 - -0.06) | -0.195  (-0.29 - -0.10) | -0.114  (-0.22 - -0.00) | <0.001 *** |
| Variables that have a single value per *spark,* and show clustering within *cell.* There is minimal additional variability between rats such that analysis at cell level hierarchy is most appropriate*.* Here a comparison of goodness of fit between common and 2-level test is significant (p<0.05) but the same comparison between the 2-level hierarchy and 3-level hierarchy is not significant (p>0.05). | | | | | | | | |
| LogFDHM  (ms) | 8% | 1.372  (1.35-1.40) | 1.334  (1.31-1.36) | 1.346  (1.30-1.38) | 1.338  (1.30-1.38) | N/A | N/A | <0.001 *** |
| LogFWHM  (µm) | 7% | 0.420  (0.40-0.44) | 0.410  (0.39-0.43) | 0.408  (0.38-0.44) | 0.413 (0.39-0.44) | N/A | N/A | <0.001 *** |

Table S7. Estimated means and confidence intervals for Ca^2+^ spark variables using standard and hierarchical statistical tests.

###### The independent-samples t-test is shown as the common test used to compare spark data. The clustering of data measured by calculating the intraclass correlation (ICC) is shown for each variable. The hierarchical technique is more appropriate with each variable as indicated by better goodness of fit (as measured by χ^2^-2LL test). When using the more appropriate hierarchical test the mean (point estimate) changes little but the estimate is appropriately less precise (wider 95% confidence interval). CI = Confidence Interval.


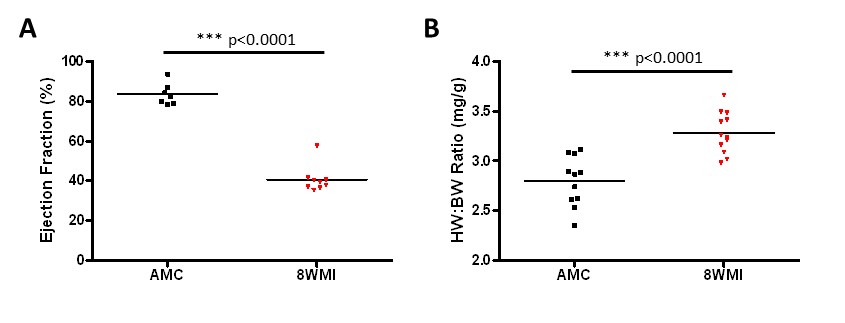


Figure S1. Confirmation of heart failure phenotype in 8W post MI rats.

1. *Echocardiographic data confirmed a significant reduction in ejection fraction in 8 week post myocardial infarction (8WMI) rats compared to age matched controls (AMC). (B) Biometric data showed an increase in heart weight to body weight (HW:BW) ratio. n=7-12 rats in each group.*


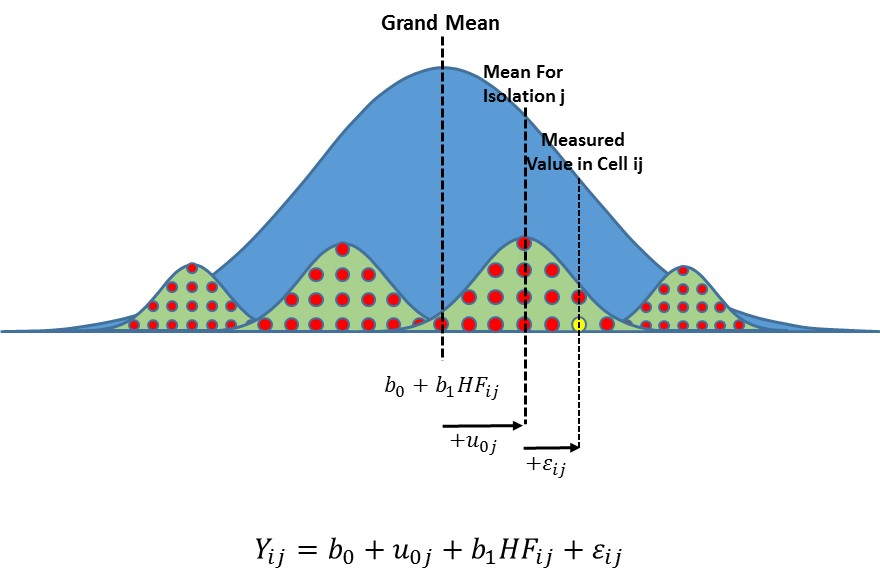


**Figure S2.** Data structure found in hierarchical data in a theoretical set of HF rats with reference to how the random intercept model equation relates to an individual data point (yellow dot). Large blue-coloured Gaussian distribution represents the distribution of measurements of a particular outcome measure in all HF rats tested. A sample of 4 smaller distributions (green) represents the distribution of the outcome measure found in each rat with each cell from which an individual measurement is taken being represented by a red dot. The model varies from standard linear regression by the inclusion of a random intercept term (u_0j_) which enables each animal to have its own distribution resulting in a more accurate representation of the true data structure.

**Figure S3.** Initial exploration of the data showed a marked skew for variables relating to spark morphology (left panels)**.** A logarithmic transformation enhanced the symmetry of the data (right panels). This is ecessary to ensure validity of the hierarchical models

**References**

1. Hox JJ. Multilevel Analysis: Techniques and Applications. 2nd ed. Hove, United Kingdom: Routeledge Academic; 2010.

2. Field A. Discovering Statistics Using IBM SPSS Statistics. 3rd ed. London: Sage; 2009.

3. Huang K, Huang D, Shengquan F, Yang C, Liao Y. Abnormal Calcium ‘Sparks’ in Cardiomyocytes of Post-myocardial Infarction Heart. *J Huazhong Univ Sci Technol* 2008;**28**:401–408.

4. Lee J, Stagg M a, Fukushima S, Soppa GKR, Siedlecka U, Youssef SJ, Suzuki K, Yacoub MH, Terracciano CMN. Adult progenitor cell transplantation influences contractile performance and calcium handling of recipient cardiomyocytes. *Am J Physiol Heart Circ Physiol* 2009;**296**:H927-36.

5. Ibrahim M, Navaratnarajah M, Siedlecka U, Rao C, Dias P, Moshkov A V, Gorelik J, Yacoub MH, Terracciano CM. Mechanical unloading reverses transverse tubule remodelling and normalizes local Ca(2+)-induced Ca(2+)release in a rodent model of heart failure. *Eur J Heart Fail* 2012;**14**:571–580.

6. Lyon AR, MacLeod KT, Zhang Y, Garcia E, Kanda GK, Lab MJ, Korchev YE, Harding SE, Gorelik J. Loss of T-tubules and other changes to surface topography in ventricular myocytes from failing human and rat heart. *Proc Natl Acad Sci U S A* National Academy of Sciences; 2009;**106**:6854–6859.

7. Lyon AR, Bannister ML, Collins T, Pearce E, Sepehripour AH, Dubb SS, Garcia E, O’Gara P, Liang L, Kohlbrenner E, Hajjar RJ, Peters NS, Poole-Wilson P a, Macleod KT, Harding SE. SERCA2a gene transfer decreases sarcoplasmic reticulum calcium leak and reduces ventricular arrhythmias in a model of chronic heart failure. *Circ Arrhythm Electrophysiol* 2011;**4**:362–372.

8. Gomez a. M, Guatimosim S, Dilly KW, Vassort G, Lederer WJ. Heart Failure After Myocardial Infarction: Altered Excitation-Contraction Coupling. *Circulation* 2001;**104**:688–693.

9. Mørk HK, Sjaastad I, Sande JB, Periasamy M, Sejersted OM, Louch WE. Increased cardiomyocyte function and Ca2+ transients in mice during early congestive heart failure. *J Mol Cell Cardiol* 2007;**43**:177–186.

10. Louch WE, Mørk HK, Sexton J, Strømme T a, Laake P, Sjaastad I, Sejersted OM. T-tubule disorganization and reduced synchrony of Ca2+ release in murine cardiomyocytes following myocardial infarction. *J Physiol* 2006;**574**:519–533.

11. Zalvidea S, André L, Loyer X, Cassan C, Sainte-Marie Y, Thireau J, Sjaastad I, Heymes C, Pasquié J-L, Cazorla O, Aimond F, Richard S. ACE inhibition prevents diastolic Ca2+ overload and loss of myofilament Ca2+ sensitivity after myocardial infarction. *Curr Mol Med* 2012;**12**:206–217.

12. Bito V, Biesmans L, Gellen B, Antoons G, Macquaide N, Rouet-Benzineb P, Pezet M, Mercadier J-J, Sipido KR. FKBP12.6 overexpression does not protect against remodelling after myocardial infarction. *Exp Physiol* 2013;**98**:134–148.

13. Bito V, Waard MC de, Biesmans L, Lenaerts I, Ozdemir S, Deel E van, Abdel-Mottaleb Y, Driesen R, Holemans P, Duncker DJ, Sipido KR. Early exercise training after myocardial infarction prevents contractile but not electrical remodelling or hypertrophy. *Cardiovasc Res* 2010;**86**:72–81.

14. Louch WE, Hake J, Jølle GF, Mørk HK, Sjaastad I, Lines GT, Sejersted OM. Control of Ca2+ release by action potential configuration in normal and failing murine cardiomyocytes. *Biophys J* Biophysical Society; 2010;**99**:1377–1386.

15. Mørk H, Sjaastad I, Sejersted OM, Louch WE. Slowing of cardiomyocyte Ca2+ release and contraction during heart failure progression in postinfarction mice. *Am J Physiol - Hear Circ Physiol* 2009;**296**:H1069–H1079.

16. Thireau J, Karam S, Fauconnier J, Roberge S, Cassan C, Cazorla O, Aimond F, Lacampagne A, Babuty D, Richard S. Functional evidence for an active role of B-type natriuretic peptide in cardiac remodelling and pro-arrhythmogenicity. *Cardiovasc Res* 2012;**95**:59–68.

17. Louch WE, Hake J, Mørk HK, Hougen K, Skrbic B, Ursu D, Tønnessen T, Sjaastad I, Sejersted OM. Slow Ca(2+) sparks de-synchronize Ca(2+) release in failing cardiomyocytes: Evidence for altered configuration of Ca(2+) release units? *J Mol Cell Cardiol* Elsevier Ltd; 2013;**58**:41–52.
